# Producing chemically accurate atomic Gaussian process regression models by active learning for molecular simulation

**DOI:** 10.1002/jcc.27006

**Published:** 2022-09-27

**Authors:** Matthew J. Burn, Paul L. A. Popelier

**Affiliations:** ^1^ Manchester Institute of Biotechnology The University of Manchester Manchester UK; ^2^ Department of Chemistry The University of Manchester Manchester UK

**Keywords:** FFLUX, Gaussian process regression, IQA, kriging, machine learning, particle swarm optimization, QTAIM, quantum chemical topology

## Abstract

Machine learning is becoming increasingly more important in the field of force field development. Never has it been more vital to have chemically accurate machine learning potentials because force fields become more sophisticated and their applications expand. In this study a method for developing chemically accurate Gaussian process regression models is demonstrated for an increasingly complex set of molecules. This work is an extension to previous work showing the progression of the active learning technique in producing more accurate models in much less CPU time than ever before. The per‐atom active learning approach has unlocked the potential to generate chemically accurate models for molecules such as peptide‐capped glycine.

## 1 INTRODUCTION

In spite of sustained progress in accelerating ab initio calculations, force fields continue to be the only practical way forward to compute energies and forces for systems of many thousands of atoms over multi‐nanosecond trajectories. The price paid for the enormous speedup that force fields offer is that their equations involve parameters. Determining the best values for these parameters, such that the force field becomes truly predictive, continues to be a challenge. For instance, in the area of peptide and protein modeling, recommendations on which force field (or parameterization thereof) to use heavily influence the outcome of the molecular dynamics (MD) runs. For example, using AMBER99SB^1^ on a decapeptide (that is part of the amyloid β protein) yields elongated conformations in aqueous solution while OPLS‐AA returns folded conformations. Looking at more case studies reveals that the state‐of‐the‐art is actually more concerning because the best set of parameters for one simulated system is not necessarily the best for another system. For example, the study of Rauscher et al.^2^ on for a highly charged Arg‐Ser hexadecapeptide found that CHARMM22*/mod TIP3P gave results closest to experiment. This force field was then used by Shaw et al.,^3^ alongside five others, on 21 test systems not containing the previous hexadecapeptide. It turned out that the new preferred force field was now different to the previous one, namely aa99SD‐disp. This recommendation is still not the end point because, soon after, Demerdash et al.^4^ set out to systematically improve the parameters of this force field against experimental SAXS and SANS intensities. It is clear that any motivation to improve force fields is justified, whether by reparameterization or by alternative designs.

The latter route of improvement has been fuelled by the introduction of machine learning (ML) to the construction of atomistic potentials. A trickle of early examples^5^, ^6^, ^7^, ^8^, ^9^ was based on neural networks^10^ and eventually led to an explosion of publications.^11^, ^12^ Similarly, the innovative introduction^13^, ^14^ of Gaussian process regression (GPR) (also known as kriging) to atomistic potential design culminated in another explosion of activity.^15^, ^16^, ^17^ ML, which is much more developed for potentials^18^ in material science than in biomolecular modeling,^19^ enables novel force field architectures^20^ in which classical bonded potentials do not appear. Moreover, polarization can in principle be captured without reverting to polarizability tensors but by directly learning what a given atom's electron density looks like in a sufficient number of possible environments. However, the computational cost of ML at production (i.e., prediction) stage positions this methodology in between classical force field and ab initio molecular dynamics (AIMD). ML force fields allow a more complete adoption of the potential energy surface compared to classical force fields by replacing predetermined equations with a method that learns from real world data and adapts to the system at hand. For this reason, ML force fields can be used to bridge a gap between the accuracy of ab initio methods and the speed of force fields.^21^ However, ML is no magic bullet because the accuracy of the force field is dependent on how the ML technique is employed.

Previous work has shown the promise of using GPR models for predicting atomic properties of a system.^22^, ^23^, ^24^ GPR models rely on the provided data, which are used not only to fit hyperparameters but also to make predictions. For this reason, a high‐quality training set is vital in order to obtain a good GPR model. Defining high‐quality is difficult and depends on the problem the GPR model is designed to tackle. In the case of atomistic simulation, the quality of the model is determined by multiple factors: (i) accuracy of the predictions, (ii) the flexibility of the system, and (iii) the size of the training set. The final factor may seem like less of a concern than the other two because ML models are generally trained once, and used many times afterwards in a production setting. It is important to reduce the training set size, not only for the training itself but also for the time it takes to generate predictions. This is because the training set is used while making predictions (which is not the case with neural nets).

Producing a high‐quality atomistic GPR model is therefore a trade‐off between accuracy, flexibility, and training set size because, generally speaking, increased accuracy and flexibility comes at the cost of increasing the number of points. It is possible that multiple similar points occur within a training set resulting in redundant information. This issue calls for a smart approach to add training points to a training set. One can avoid wasting CPU time of computing the atomic properties of redundant training points by considering the number of training points while constructing a model. Moreover, there is the added benefit that fewer training points reduce prediction times when using the models in atomistic simulations.

Active learning is the process of iteratively improving the training set of a model by adding points that will improve the model in the subsequent iteration. Active learning should produce a training set that can produce a model with a desired accuracy with a minimum number of points. Once a desired model accuracy is achieved the active learning process can stop. Our previous work has shown the promise of active learning for producing GPR models for atomistic simulations.^24^

Several other groups have also investigated active learning with a wide range of applications. Although not explicitly mentioned in their work,^25^ Artrith and Behler implemented an actual active learning protocol where the same database was fitted by two neural network potentials for metal surfaces. If their prediction differs for a given new structure, then it needs to be added to the database. Many years later this type of strategy was perfected^26^ by a committee of neural networks potentials for water in the condensed phase. Kernel based methods such as GPR are well suited toward active learning methods due to the ability to calculate the variance of the prediction. As a result, active learning has been used to improve the predictions of a GPR model, both on‐the‐fly and not‐on‐the‐fly, during atomistic simulations. An active learning model^27^ based on Bayesian optimization of DFT‐calculated oxidation potentials of 1400 homobenzylic ethers demonstrated a more than fivefold improvement in computational efficiency compared to random selection. Jinnouchi et al. showed how AIMD can be accelerated by active learning of the computed forces using the predicted error of the Gaussian process to select new data points. This work benefitted simulations of phase transitions of hybrid perovskites^28^ and generated on‐the‐fly force fields for melting point applications,^29^ culminating in on‐the‐fly active learning of interatomic potentials for large‐scale atomistic simulations.^30^ Ulissi et al. demonstrated a Δ‐ML approach that enables stable convergence in offline active learning strategies by avoiding unphysical configurations with very small initial datasets, with application to structural relaxation, transition state calculation, and MD simulation. Further success in active learning research include work on the drug discovery process using support vector machines,^31^ on the acceleration of AIMD on reactive surfaces,^32^ on force fields for atomistic rare events,^33^ on the efficient and accurate prediction of molecular properties such as atomization energies and polarizabilities,^34^ on training across intermetallics to guide the discovery of electrocatalysts for CO_2_ reduction and H_2_ evolution,^35^ on de novo exploration and self‐guided learning of potential energy surfaces^36^ with application to materials of diverse chemical nature and coordination environments, on improved training over databases of diverse organic molecules,^37^ on a deep potential generator for Al, Mg, and Al‐Mg alloys,^38^ and on accelerating crystal structure prediction.^39^

This paper demonstrates progress in the active learning method that we use, which is called the maximum expected prediction error (MEPE).^40^ The main advance will be the per‐atom approach as opposed to the per‐system approach of before. We showed in our previous work that active learning increases model accuracy while decreasing model size. Here we build on this success and improve the results further. We will show that the per‐atom approach decreases the time it takes to produce a model, without any loss of accuracy.

## 2 METHODS

Active learning is used to iteratively improve a GPR model, which means that we must have an initial training set and a set of sample points (called a sample set) that are selectively added to the training set by the active learning method. The goal is to improve the prediction errors of the model, which is measured using a validation set. The latter is a set that is independent to both the training set and sample set. Our method produces a model for any atomic property but the current work is confined to the production of atomic models for predicting IQA^41^ energies. The acronym IQA stands for interacting quantum atoms and is the most used quantum topological energy partitioning method to date.

### 2.1 Point generation

An AIMD simulation generates the initial data points (i.e., molecular configurations) to put into the initial training set, sample set and validation set. Using a physically based sampling approach ensures that we sample chemical space in accordance with our desired use of the model in the force field. Moreover, this approach ensures that we can vary the extent of molecular distortions by varying the temperature of the simulation. In order to sample enough chemical space to create a flexible model that is useful for atomistic simulations the AIMD simulation must be run for a significant amount of time. For larger systems, this quickly becomes infeasible in a reasonable amount of time and other sampling techniques must be used. Previous work has demonstrated the ability of generating molecular configurations by normal mode sampling using the in‐house program TYCHE.^20^ Sampling chemical space using normal modes is a much faster alternative to AIMD sampling.

In terms of sampling molecular configurations, it is vitally important that a large amount of chemical space is sampled to prevent multiple points in the same region of configuration space. For this reason, MD sampling (be that classical or ab initio) needs to be carried out for long enough to allow for a wide spread of sample points and to prevent missing any important regions of chemical space. TYCHE prevents a point being sampled too close to any previously sampled point by rejecting points that are within a cut‐off of the rest of the trajectory.

Once the trajectory has been attained the points need to be split into the training, sample, and validation sets. The initial training set should ideally be a space‐filling sampling method such as a Latin hypercube sampling or maximin distance sampling, but these methods do not scale well to higher dimensions. In this study the dimensionality of the largest system is 51 (glycine) and so space‐filling designs quickly become infeasible. Instead, a better scaling method was chosen that takes the minimum, maximum, and mean of each dimension of the trajectory to initialize the training set. The features of each point in the initial trajectory are calculated and the minimum and maximum features can be chosen directly, the mean of each feature is then calculated and the closest point to the mean is then added to the training set. This procedure results in an initial training set that scales linearly with respect to the number of dimensions. The size of this set has an upper limit of three times the number of dimensions (because repeat points are removed). The rationale behind this method of initialization is to sample the extremities of each feature alongside filling out the center of the sample space while avoiding a combinatorial explosion caused by sampling combinations of features. The sample set (10,000 points) and the validation set (500 points) are then initialized randomly from the remaining points in the trajectory.

### 2.2 Computational details

All AIMD simulations were carried out using CP2K^42^ with the BLYP functional with Grimme's D3^43^ dispersion correction and the 6‐31G* basis set. All systems were single‐molecule simulations in a vacuum in a 30 × 30 × 30 Å^3^ box. The wavefunctions of the selected molecular geometries were obtained by the ab initio program Gaussian 09^44^ (G09) with the B3LYP functional and the 6‐31+G(d,p) basis set. All quantum chemical topology calculations were performed using AIMAll^45^ 17.11.14. All active learning and ML pipelining is performed by the in‐house software ICHOR.^24^ ICHOR is a ML pipeline suite designed from the ground up for producing GPR models for atomistic MD simulations in the DL_FFLUX program. Further details on ICHOR can be found in Supporting Information S1 such as a flowchart of ICHOR's pipeline in Figure S1. Note that only the IQA energies are used for the training outputs, not their gradients.

### 2.3 Gaussian process regression

GPR^46^ is a non‐linear regression technique that allows for the interpolation between points of an arbitrary function. In this study we use atomic GPR models to model the potential energy surface of various systems. GPR uses a covariance kernel to calculate the covariance between two points. Starting from the standard RBF kernel (also known as the squared exponential kernel),

(1) $$k\left(x,x^{*}\right)=\exp\left(-\sum_{d=1}^{\text{ndim}}\theta_{d}{\left(x_{d}-x_{d}^{*}\right)}^{2}\right)$$

where x and $x^{*}$ denote two feature vectors. In this study, the features are calculated using the atomic local frame (ALF), which is explained in detail in section 2 of the Supporting Information S1 where Figure S2 shows an example of an ALF. We modify this kernel by calculating the distance taking into account that some features are not linear but cyclic. Indeed, every third feature is an angular feature that can range from −π to π. Hence a linear distance calculation in the standard RBF kernel of Equation (1) would calculate an incorrect distance between two given values of a feature of this type. To fix this, a cyclic feature correction can be applied to the RBF kernel,

(2) $$\begin{array}{cc} k\left(x,x^{*}\right) & \;=\exp\left(-\sum_{d=1}^{\text{ndim}}\theta_{d}r_{d}{\left(x_{d},x_{d}^{*}\right)}^{2}\right)\\ r_{d}\left(x_{d},x_{d}^{*}\right) & =\left\{\begin{array}{cc} x_{d}-x_{d}^{*}, & \;d\;\mathrm{mod}\;3\neq 0\\ \left[\left(x_{d}-x_{d}^{*}+\pi \right)\;\mathrm{mod}\;2\pi \right]-\pi , & \;d\;\mathrm{mod}\;3=0 \end{array}\right. \end{array}$$

The hyperparameter $\theta_{d}$ scales the distance between $x_{d}$ and $x_{d}^{*}$, and is optimized for the given training set. In previous work^24^ this optimization was carried out by maximizing the concentrated log likelihood but here we maximize the marginal log likelihood estimation,

(3) $$\mathrm{LL}\left(y|x,\theta \right)=-\frac{1}{2}{\left(y-\mu \right)}^{T}R^{-1}\left(y-\mu \right)-\frac{1}{2}\log\left|R\right|-\frac{n}{2}\log\;2\pi$$

where x is the training input of size ntrain×nfeatures, y is the training outputs of size ntrain×1, θ is the hyperparameter vector and *n* is the number of training points. μ is the mean function, which is here a constant mean given by Equation (4) and R is the covariance matrix given by Equation (5),

(4) $$\mu =\bar{y}$$

(5) $$R=k\left(x,x\right)=\left[\begin{array}{ccc} k\left(x_{1},x_{1}\right) & \cdots & k\left(x_{1},x_{n}\right)\\ \;\vdots & \ddots & \;\vdots \\ k\left(x_{n},x_{1}\right) & \cdots & k\left(x_{n},x_{n}\right) \end{array}\right]$$

Training is limited by inverting the square matrix R governed by an $Ο\left(n^{3}\right)$ operation, which can become expensive at large values of *n*. Predictions using the GPR model are made by calculating the covariance vector r of an unknown point $x^{*}$ with the training set x in order to calculate the deviation of this unknown point from the mean μ,

(6) $$r=k\left(x^{*},x\right)=\left[\begin{array}{c} k\left(x^{*},x_{1}\right)\\ \;\vdots \\ k\left(x^{*},x_{n}\right) \end{array}\right]$$

(7) $$\hat{f}\left(x^{*}\right)=\mu +r^{\prime }R^{-1}\left(y-\mu \right)$$

where $r^{\prime }$ denotes the transpose of column vector r into a row vector. The expression $R^{-1}\left(y-\mu \right)$ can be precomputed because no term depends on the unknown point $x^{*}$. This simplification produces vector α, which is referred to as the GPR weights,

(8) $$\alpha =R^{-1}\left(y-\mu \right)$$

(9) $$\hat{f}\left(x^{*}\right)=\mu +r^{\prime }\alpha$$

The simplified Equation (9) scales linearly with the number of training points. The fewer training points, the faster both training and predictions will be.

### 2.4 Optimizing hyperparameters

A GPR model is defined by both the training set and the hyperparameters, and both contribute to the quality of the model produced. As mentioned in Section 2.3, the hyperparameters are optimized by maximizing the marginal log likelihood of the GP. This likelihood function is a non‐convex function with many local maxima, which makes it a difficult function to optimize and therefore requires a robust global optimizer. In this study, a particle swarm optimization^47^ (PSO) is used.

PSO iteratively improves the solution found by taking advantage of the swarming behavior of a number of particles. Each particle has a position and velocity, and the velocity is updated each iteration by the following equation,

(10) $$v_{i_{t+1}}=\omega v_{i_{t}}+\varphi_{p}r_{1}\left(p_{i}-x_{i_{t}}\right)+\varphi_{g}r_{2}\left(g-x_{i_{t}}\right)$$

where ω is the inertia weight, $v_{i_{t}}$ is the velocity of particle i at time t, $\varphi_{p}$ is the cognitive learning rate, $p_{i}$ is the previously best‐known position of particle i, $\varphi_{g}$ is the social learning rate, g is the globally best‐known position, $x_{i_{t}}$ is the position of particle i at time t, while $r_{1}$ and $r_{2}$ are random values sampled from a uniform distribution between 0 and 1.

The velocity is made up of three components, each represented by a term on the right‐hand side of Equation (10). From left to right there is (i) the current velocity of the particle, (ii) the pull on the particle to its previously best‐known position, and (iii) the pull on the particle to the globally best‐known position. It is the trade‐off between these three components that allows particles to explore the search space while moving toward a global optimum. Each dimension of the particle position and velocity vectors is initialized using a uniform distribution between $\theta_{\min}$ and $\theta_{\max}$. The particle's best‐known position and the globally best‐found position is updated once per swarm cycle and this process iterates until a stopping criterion is met.

In this study, a relative‐difference stopping criteria is used, shown in Equation (11),

(11) $$\mathrm{rel}.\;\text{diff}.=\frac{g_{t+1}-g_{t}}{g_{t}}$$

If the relative difference falls below a threshold $\sigma_{\text{thresh}}$ for $n_{\text{stall}}$ iterations, the swarm has converged and the optimization is complete. Table 1 shows the values for the parameters used in this study. The GPR and PSO algorithms are implemented using the in‐house program FEREBUS^48^ to allow domain‐specific optimizations of both the GPR kernels and the optimization procedure.

**TABLE 1:** Parameters for the particle swarm optimization

Parameter	Value
ω	0.729
$\varphi_{p}$	1.490
$\varphi_{g}$	1.490
$\theta_{\min}$	0.0
$\theta_{\max}$	3.0
$\sigma_{\text{thresh}}$	$1\times 10^{-7}$
$n_{\text{stall}}$	20

### 2.5 Atomistic GPR models

Each atom in a system has a unique model containing inputs relative to the atom's local frame and as an output a particular atomic property such as IQA energy. This means that the total energy of a system can be calculated by summing over all predicted atomic energies. Although this study uses IQA energy as the output property for the atomic model, this need not be the case: the output can be any atomic property such as a multipole moment^13^, ^49^ (monopole, dipole moment, etc.) or dispersion energy.^50^, ^51^ In this study the inputs and outputs are calculated (for each atom) from the *entire system* and therefore each model can only be used on the system it was trained for. However, it is important to note that this is not a limit of the methodology. Indeed, the inputs and outputs may be calculated from a subsystem, and the subsequent models may be used in a larger system. This generalization invokes the concept of transferability for which the quantum atoms score well.

Features are calculated atomically using the ALF.^52^ All features are geometric features based on a local frame constructed around the atom currently being predicted. Three atoms define the local frame: the origin atom, the atom defining the *x*‐axis and the atom defining the *xy*‐plane. All features are calculated in relation to this local frame whereby the first three features are (i) the distance from the origin to the *x*‐axis atom, (ii) the distance from the origin to the *xy*‐plane atom, and (ii) the angle between the vector from the origin to *x*‐axis atom and that from the origin to *xy*‐plane atom. Every subsequent feature consists of spherical polar coordinates (with respect to the ALF) of each atom outside of the ALF. Having geometric features means that all features are translationally and rotationally invariant, as well as being unique, such that the exact original geometry can be retrieved. Full details of the feature calculation can be found in Supporting Information S1.

### 2.6 Active learning

Active learning is the method of iteratively improving the training set by adding the point that will improve the training set the most for a given domain space. The difficulty arises when the true output values of the points that are being added to the training set are unknown and we can therefore not test beforehand how much the model will improve. In a previous study we used an active learning method known as MEPE,^40^ which estimates the expected prediction error (EPE) of a sample set and then adds the points with the largest EPE.

#### 2.6.1 MEPE method

The MEPE method of active learning allows for the selection of a point from a set of sample points that will improve the training set the most in the subsequent iteration. The simplest active learning method would be to calculate the prediction error (PE) of our sample points and add the points with the largest prediction error using the following equation,

(12) $$\mathrm{PE}\left(x^{*}\right)=\left|f\left(x^{*}\right)-\hat{f}\left(x^{*}\right)\right|$$

where $f\left(x^{*}\right)$ is the true value and $\hat{f}\left(x^{*}\right)$ is the predicted value for a given point $x^{*}$.

This simple method comes with the downside that we must calculate the true value of x, which in practice limits the size of our sample set and consequently our search space by how much computation time can be afforded to these calculations. MEPE allows the estimation of the prediction error of unknown point x using two metrics: cross‐validation (CV) error and variance.

The CV‐error is a measure of how well a part of the training set is understood, which is known as the exploitation term. On the other hand, the variance is a measure of what parts of the search space are currently not well known to the training set, which is known as the exploration term. A balance between exploration and exploitation terms is what provides the EPE of a point for an iteration.

Specifically, for the MEPE method we use leave‐one‐out CV, which produces a model with a single training point removed, after which the prediction error of that training point is compared with the new model,

(13) $${{\mathrm{PE}}_{\mathrm{CV}}}^{2}\left(x_{i}\right)={\left(f\left(x_{i}\right)-{\hat{f}}^{-i}\left(x_{i}\right)\right)}^{2}$$

where ${\hat{f}}^{-i}$ is the GPR model with point $x_{i}$ removed from the training set. Unfortunately, training is expensive especially when moving to larger training sets. Therefore, producing a model for each training point quickly becomes infeasible and an approximation to the CV error is required,

(14) $${{\mathrm{PE}}_{\mathrm{CV}}}^{2}\left(x_{i}\right)\approx {\left(\frac{{\left(R^{-1}\right)}_{i,:}\left(d+H_{:,i}\frac{d_{i}}{1-H_{\mathrm{ii}}}\right)}{{\left(R^{-1}\right)}_{\mathrm{ii}}}\right)}^{2}$$

where $H_{:,i}$ is column i of matrix H, ${\left(R^{-1}\right)}_{i,:}$ is row i of matrix $R^{-1}$ while d and H are calculated using the following set of equations,

(15) $$d=y-F\hat{\beta }$$

(16) $$\hat{\beta \;}={\left(F^{T}R^{-1}F\right)}^{-1}F^{T}R^{-1}y$$

(17) $$H=F{\left(F^{T}F\right)}^{-1}F^{T}$$

(18) $$F={\left[p\left(x_{1}\right),\ldots ,p\left(x_{n}\right)\right]}^{T}$$

(19) $$p\left(x\right)={\left[1,\ldots ,1\right]}^{T}$$

Equations ((15), (16), (17), (18)) show the calculation of d and H for universal GPR, which is a GPR model with mean defined by a set of functions $p\left(x\right)$. As we are using a GPR with constant mean, these equations can be simplified by setting $p\left(x\right)$ to a vector of **1**'s of length n as shown in Equation (19).

Equation (14) provides a relatively quick way of approximating the CV error of a training point. Unfortunately, the CV error of an arbitrary sample point (x) is required. In order to approximate the CV error of a sample point, a Voronoi partition of the training set is used to approximate the CV error of a sample point by using instead the CV error of the closest training point. Formally if the arbitrary point x lies within the Voronoi cell $V_{i}$ defined by training point $x_{i}$ then the CV error of the arbitrary point ${{\mathrm{PE}}_{\mathrm{CV}}}^{2}\left(x\right)$ is approximated to the CV error of the training point ${{\mathrm{PE}}_{\mathrm{CV}}}^{2}\left(x_{i}\right),$

(20) $$x\in V_{i}\to {{\mathrm{PE}}_{\mathrm{CV}}}^{2}\left(x\right)={{\mathrm{PE}}_{\mathrm{CV}}}^{2}\left(x_{i}\right)$$

An advantage of using GPR as a ML method is that the prediction also comes with an error bar as each point is a Gaussian with a mean and a variance. The predictive mean of the Gaussian is calculated using Equation (9) and the variance of a given point is calculated using the following equation,^53^

(21) $$s^{2}\left(x\right)=\sigma^{2}\left[1-r^{T}R^{-1}r+\frac{{\left(1-1^{T}R^{-1}r\right)}^{2}}{1^{T}R^{-1}1}\right]$$

As stated previously, the exploitation (CV error) and exploration (variance) terms need to be balanced so that neither one nor the other dominates the active learning process. This is done using a balance factor α, which varies between active learning iterations using the following equation.

(22) $$\alpha =\left\{\begin{array}{cc} 0.5, & q=1\\ 0.99\times \min\left[0.5\times \frac{{{\mathrm{PE}}_{\text{true}}}^{2}\left(x_{i+q-1}\right)}{{{\mathrm{PE}}_{\mathrm{CV}}}^{2}\left(x_{i+q-1}\right)},1\right], & q>1 \end{array}\begin{array}{c} \;\\ \; \end{array}\right.$$

where $x_{i+q-1}$ is the training point added on the previous iteration. On the first iteration, that is *q* = 1, q, α is initialised to 0.5 then every subsequent iteration, α is calculated by comparing the true prediction error (${{\mathrm{PE}}_{\text{true}}}^{2}$) to the CV error approximation (${{\mathrm{PE}}_{\mathrm{CV}}}^{2}$) producing a balance factor that is between 0 and 0.99. This balance factor is combined with the CV error and variance to calculate the EPE,

(23) $$\mathrm{EPE}\left(x\right)=\alpha {{\mathrm{PE}}_{\mathrm{CV}}}^{2}\left(x\right)+\left(1-\alpha \right)s^{2}\left(x\right)$$

The point with the MEPE from a set of sample points is selected and added to the training set where the true value is calculated, and the model is retrained.

#### 2.6.2 Per‐system approach

Traditionally, the active learning was performed system‐wise. The system‐wise approach (also known as per‐system active learning) is the simplest approach to implementing active learning as each atom in a system shares the same training set. Having the same training set between all atoms in a system and adding a single point to all models means that the CV errors and variances for each model need to be summed up to produce a single value for all models given a sample point. The equation for the CV error for a system is

(24) $${{\mathrm{PE}}_{\mathrm{CV}}}^{2}{\left(x\right)}_{\mathrm{sys}}=\sum_{i=1}^{\text{natoms}}{{\mathrm{PE}}_{\mathrm{CV}}}^{2}{\left(x\right)}_{i}$$

and similarly for variance,

(25) $$s^{2}{\left(x\right)}_{\mathrm{sys}}=\sum_{i=1}^{\text{natoms}}s^{2}{\left(x\right)}_{i}$$

Then the modified (see Equation (23)) EPE equation becomes.

(26) $$\mathrm{EPE}{\left(x\right)}_{\mathrm{sys}}=\alpha_{\mathrm{sys}}{{\mathrm{PE}}_{\mathrm{CV}}}^{2}{\left(x\right)}_{\mathrm{sys}}+\left(1-\alpha_{\mathrm{sys}}\right)s^{2}{\left(x\right)}_{\mathrm{sys}}$$

Because the definition for the CV error has been changed to accommodate multiple models, so must the definition of *α*. Just in the same way as we needed to sum across all atoms of the system for the CV error, we must do the same for the true prediction error for the $\alpha_{\mathrm{sys}}$ calculation.

(27) $$\alpha_{\mathrm{sys}}=\left\{\begin{array}{c} 0.5,\;q=1\\ 0.99\times \min\left[0.5\times \frac{{{\mathrm{PE}}_{\text{true}}}^{2}{\left(x_{i+q-1}\right)}_{\mathrm{sys}}}{{{\mathrm{PE}}_{\mathrm{CV}}}^{2}{\left(x_{i+q-1}\right)}_{\mathrm{sys}}},1\right],q>1 \end{array}\right.$$

where ${{\mathrm{PE}}_{\text{true}}}^{2}{\left(x_{i+q-1}\right)}_{\mathrm{sys}}$ is given by

(28) $${{\mathrm{PE}}_{\text{true}}}^{2}{\left(x\right)}_{\mathrm{sys}}=\sum_{i=1}^{\text{natoms}}{{\mathrm{PE}}_{\text{true}}}^{2}{\left(x\right)}_{i}$$

This provides us with a method of determining the EPE value for each point in a sample set from natom models and selecting a single point to add to each training set.

#### 2.6.3 Per‐atom approach

As models are produced atom‐wise and predictions made atom‐wise, it is more natural for an atom to have its own unique training set and guide the active learning to improve this training set. The change in methodology brings higher complexity in implementation while simplifying the method. This is because each model is now independent and therefore more data needs to be stored. However, this comes with the advantage that we can remove the summations over natoms from Equations (24), (25), and (28), allowing the use of the EPE method for a single model shown in Equation (23).

**FIGURE 1:**
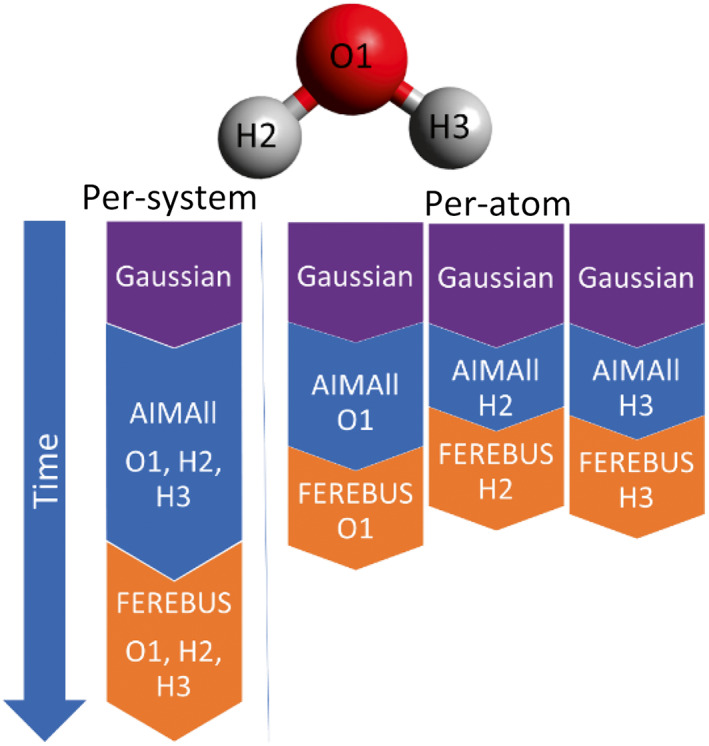
Schematic showing how the per‐atom active learning pipeline is faster than the equivalent per‐system approach. Timings are not to scale and only serve illustrative purposes

The benefit to a per‐atom approach is that each model is completely independent: each has a unique training set and can therefore be created asynchronously. Due to the asynchronous nature of the per‐atom approach, no time is wasted waiting for the computation of the atomic properties for all atoms of a system to complete. Instead, only the atomic properties for the required atomic model are computed, resulting in a more efficient use of computational resources and dramatically reducing the time to produce a model. Because each atom's active learning run is independent it can be executed in parallel, leading to speed gains of up to 70%. The simple diagram in Figure 1 helps making the difference between per‐system and per‐atom more concrete.

#### 2.6.4 Multiple points per‐iteration

Larger systems with higher dimensions demand a higher number of training points in order to obtain an accurate model. Therefore, a greater speedup is required than can be gained from the per‐atom approach alone. A further approach demonstrated in this study that helps toward this goal is that of adding multiple points per‐iteration. For this study, a relatively simple technique for multiple point addition is employed in that the points with the n largest EPE values are added to the training set. The benefit of this approach is that only the calculation of the *α* value needs to be adjusted, which is shown by the following equation,

(29) $$\alpha =\frac{\sum_{i=1}^{n}\alpha_{i}}{n}$$

where $\alpha_{i}$ is the balance factor for the ith point added to the training set in the previous iteration given by Equation (22). The average balance factor (α) is then used directly in Equation (23) to calculate the EPE of each point in the training set. As the true value for each point added to the training set is calculated in parallel, the time taken per iteration of the active learning remains the same. Therefore adding n points per iteration reduces the time taken to complete the active learning process by a factor of n.

The generation of the training data and active learning is performed by the in‐house Python3 application ICHOR. ICHOR interfaces with external programs to run MD simulations, generate ab initio data, construct training sets, and perform GPR model analysis utilizing highly parallel HPC clusters.

## 3 RESULTS AND DISCUSSION

We will discuss the production of GPR models of nine systems (Table 2) comparing various active learning techniques.

**TABLE 2:** Details of the systems discussed in this study along with the number of atoms and features

System	Number of atoms	Number of features
Water	3	3
Ammonia	4	6
Methanol	6	12
Formamide	6	12
Urea	8	18
Imidazole	9	21
*N*‐methylacetamide	12	30
Peptide‐capped glycine	19	51

We employed two methods of distorting systems: (i) an AIMD simulation at a fixed temperature and (ii) normal mode sampling using the in‐house program TYCHE. AIMD simulations give the advantage of allowing temperature control, which gives fine adjustment to the size of the potential energy surface and permitting the selection of a temperature that will cover the domain space required for the model in a production MD simulation. On the other hand, TYCHE generates molecular configurations much quicker than an AIMD simulation and is therefore very useful for larger systems. TYCHE also enables fine control over which normal modes will be sampled, as well as a thermostat for how much distortion can occur in each normal mode. Table 3 outlines of how the geometries for each system were generated.

**TABLE 3:** Details of which sampling methods (program used in parentheses) and concomitant temperatures were used to generate distorted configurations for each of the right systems

System	AIMD (CP2K)			Normal mode (TYCHE)	
	300 K	1000 K	3000 K	450 K	1750 K
Water	x		x		
Ammonia	x	x			
Methanol	x	x			
Formamide	x	x			
Urea	x	x			
Imidazole	x	x			
*N*‐methylacetamide		x		x	x
Peptide‐capped glycine				x	x

*Note*: Their atomic labeling schemes are given in Figures S3–S10.

Abbreviation: AIMD, ab initio molecular dynamics.

### 3.1 Domain space

As discussed in Section 2.1, both AIMD and normal mode sampling was used to generate the initial domain space for each active learning run. Both methods provide a temperature to control the amount of distortion induced in the system, which affects how much chemical space the model will be able to predict. Most systems were run at two different temperatures to demonstrate the effects of temperature on model accuracy and the range at which a model is considered “valid.”

**FIGURE 2:**
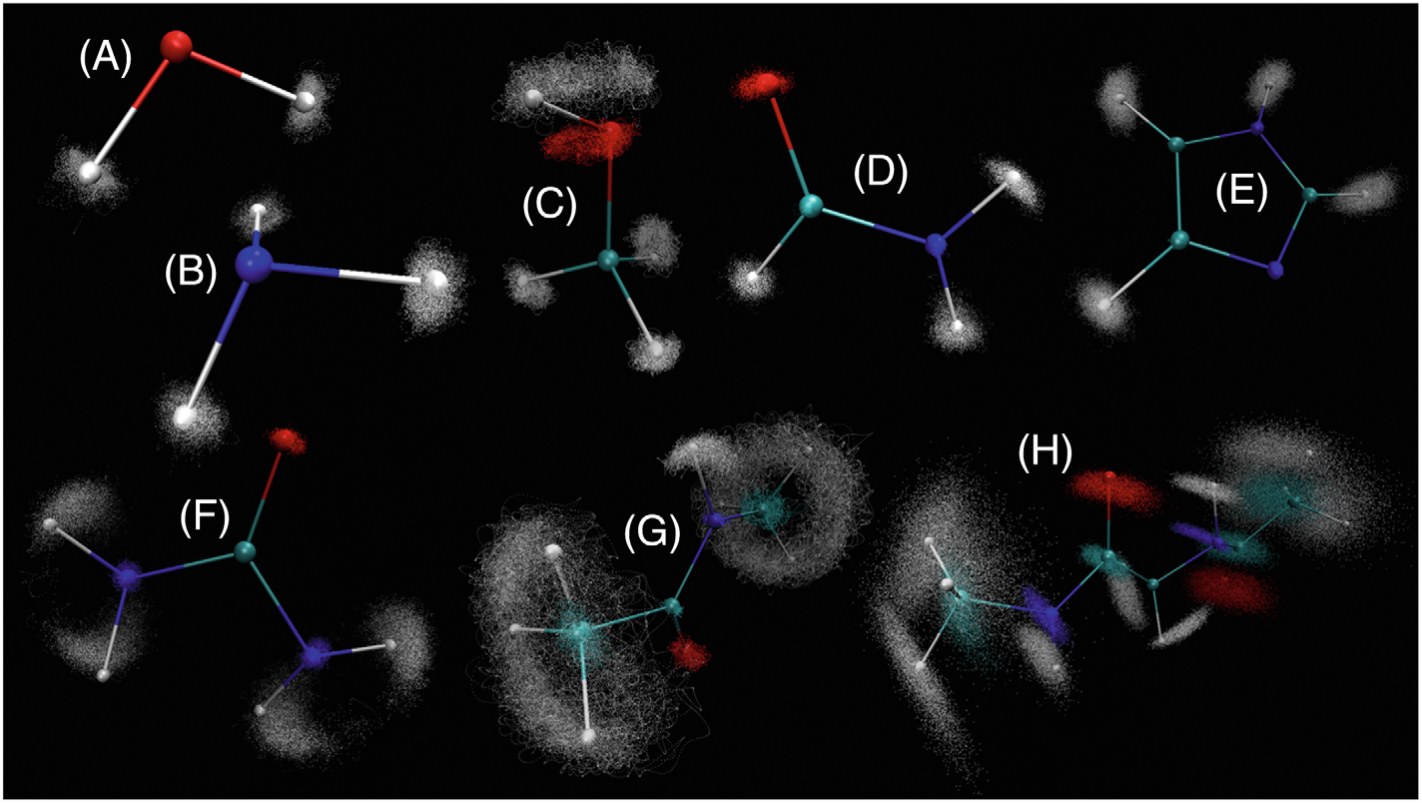
Mist plots for the highest sampled temperature for each system generated by CP2K unless otherwise stated: (A) water at 3000 K, (B) ammonia at 1000 K, (C) methanol at 1000 K, (D) formamide at 1000 K, (E) imidazole at 1000 K, (F) urea at 1000 K, (G) *N*‐methylacetamide at 1000 K, and (H) peptide‐capped glycine at TYCHE 1750 K. Mist plots for every sampled temperature for each system can be found in Figures S11–S18

To observe how much chemical space has been sampled by either of the previously mentioned methods, the trajectory of each sampling method can be plotted as a “mist” as shown in Figure 2. To overlap each geometry of a trajectory in space, the Kabsch algorithm was used to calculate the optimal rotation matrix to minimize the root‐mean‐square deviation between two configurations. In order to display the chemically relevant distortions, only part of the whole system was used for the calculation of the rotation matrix, full details of the treatment can be found in Supporting Information S1.

Once the rotated trajectories are obtained, the external program VMD was used to plot each point in the trajectory with the initial point overlayed using the ball and stick representation.

### 3.2 Prediction accuracy

One of the simplest methods for assessing the accuracy of a model is to calculate the prediction errors of the model for a given validation set. As previously mentioned, when initializing active learning, the initial set of points (known as the domain space) is partitioned into a training set, a sample set and a validation set. The true values for each point in the validation set are calculated so that the prediction error for the point can be calculated using Equation (12). Calculating the true values for the validation set can be an expensive task. Hence, for each system a validation set of 500 points was randomly selected from the initial domain space. From these 500 points the prediction error can be calculated for a given model, after which the error from each atomic prediction is summed to produce a total prediction error. Summing the absolute prediction errors (which are always positive), rather than summing each prediction and then subtracting this sum from the sum of the true values, removes the possibility of cancellation of errors. The equation for total prediction error is

(30) $${\mathrm{PE}}_{\text{total}}\left(x^{*}\right)=\sum_{i=1}^{\text{natoms}}{\mathrm{PE}}_{i}\left(x^{*}\right)$$

**FIGURE 3:**
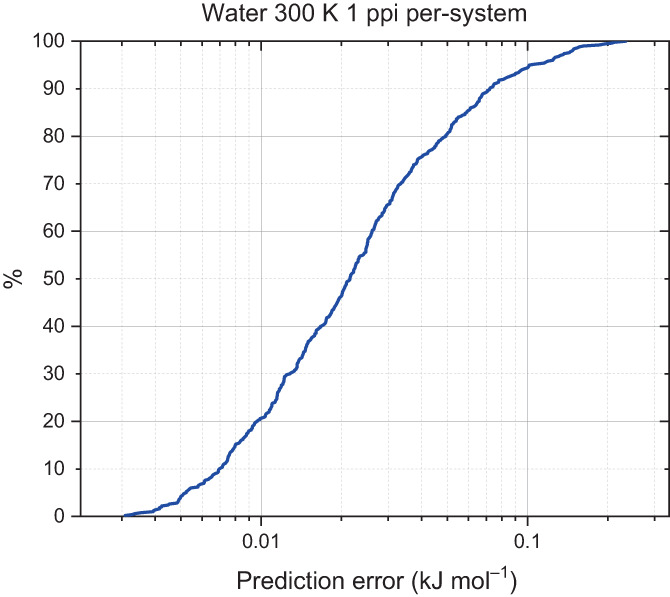
S‐curve for a 59‐point water model produced using per‐system 1‐point‐per‐iteration active learning for a 300 K domain space with a 500‐point validation set. The “%” plotted on the *y*‐axis denotes the percentile of the validation set that returns prediction errors lower than a given value read off on the *x*‐axis

By sorting the total prediction error and plotting against the prediction error percentile summarizes how well a model predicts the validation set. This plot is known as an S‐curve. The further to the left an S‐curve is, the lower the prediction error and the better the model. Another feature of an S‐curve is the “tail,” which contains to the worst predicted points. A steeper S‐curve with a smaller “tail” conveys a better model. Figure 3 shows an S‐curve for water.

**FIGURE 4:**
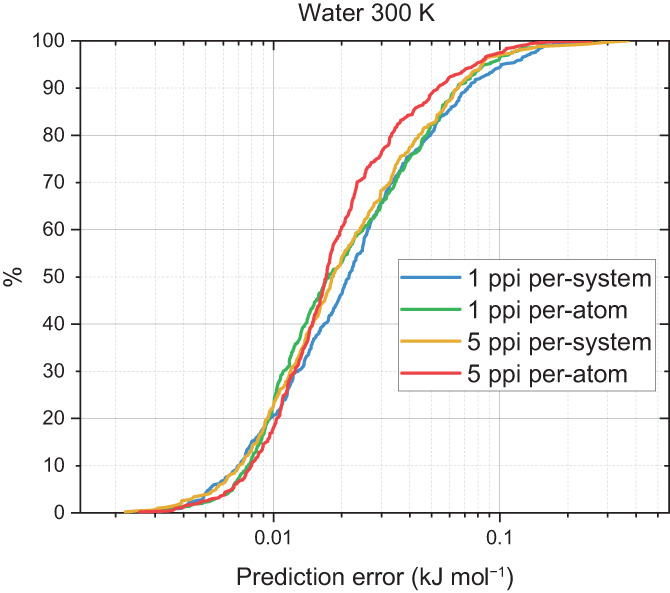
S‐curves for a set of 59‐point water models on a 300 K domain space with a 500‐point validation set

As can be seen from Figure 3, roughly 95% of the validation points are predicted with an accuracy better than 0.1 kJ mol^−1^ error and all predicted points were beneath a prediction error of 1 kJ mol^−1^. S‐curves provide a full and explicit overview of how well a given model is able to predict a set of points. Contrasting S‐curves in a single figure allows for a direct comparison between models. The S‐curves shown in Figure 4 were all tested against the same validation set. The 5‐point‐per‐iteration (5 ppi), per‐atom model outperforms the rest of the models ever so slightly. Although the difference in predictions is slight, the 5 ppi, per‐atom model was the fastest model to produce, taking a total time of 1 h 33 min compared to the slowest 1 ppi per‐system model, which took 4 h 7 min.

**FIGURE 5:**
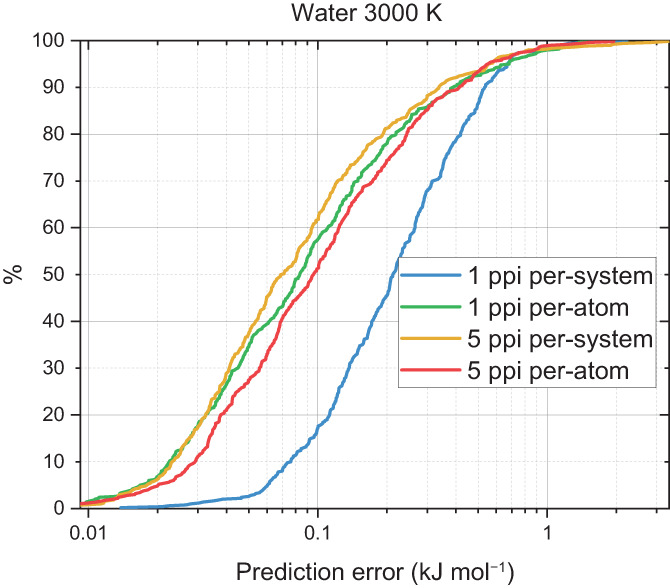
S‐curves for a set of 59‐point water models on a 3000 K domain space with a 500‐point validation set

Increasing the temperature of the initial CP2K simulation spans more of the domain space making it more difficult for the GPR model because there is a larger variation in the geometries observed. For water, this increased temperature was 3000 K and the corresponding S‐curves are shown in Figure 5. A comparison of this figure with Figure 4 shows that the prediction errors are now larger with generally about 60% of the errors being below 0.1 kJ mol^−1^ compared to 95% at 300 K and a maximum error of roughly 2 kJ mol^−1^. This is a general trend across all the systems and is a direct result of searching more configuration space. Therefore, at a set number of training points the prediction error will be worse at a higher temperature.

**FIGURE 6:**
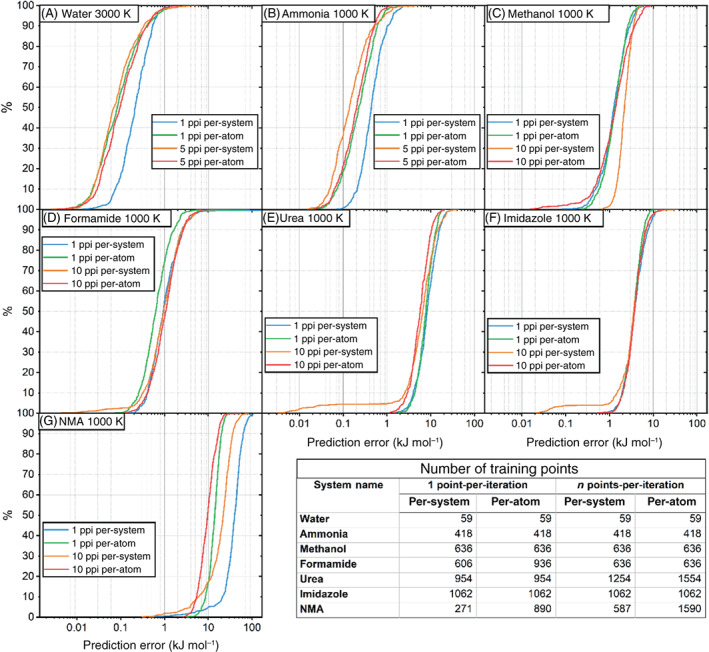
S‐curves for (A) water at 3000 K, (B) ammonia at 1000 K, (C) methanol at 1000 K, (D) formamide at 1000 K, (E) urea at 1000 K, (F) imidazole at 1000 K, and (G) *N*‐methylacetamide (NMA) at 1000 K along with the number of training points for each model. Note that NMA trained with 1‐point‐per‐iteration per‐system has far fewer points than the rest of the active learning runs resulting in a worse S‐curve. The reason for this smaller training set is that NMA is a large system and therefore per‐system 1‐point‐per‐iteration was taking a significant length of time

It is encouraging to observe that per‐atom and multiple‐points‐per‐iteration are for the majority of runs on a par with, or better than, the equivalent per‐system single‐point‐per‐iteration active learning runs. This is an excellent outcome as both per‐atom active learning and multiple‐point‐per‐iteration addition provide large reductions in the amount of time it takes to perform an active learning run. Figure 6 compares the S‐curves for the final model in each system's active learning run, further demonstrating that per‐atom active learning performs similarly to per‐system. S‐curves for the rest of the systems discussed in this study may be found in Figures S19–S26.

S‐curves provide great insight into a model's performance over a validation set. An S‐curve only provides the prediction errors of a single model whereas there are many models created over the course of an active learning run. To display the performance of a model over the course of the active learning run, a new type of plot is required.

### 3.3 Active learning prediction errors

**FIGURE 7:**
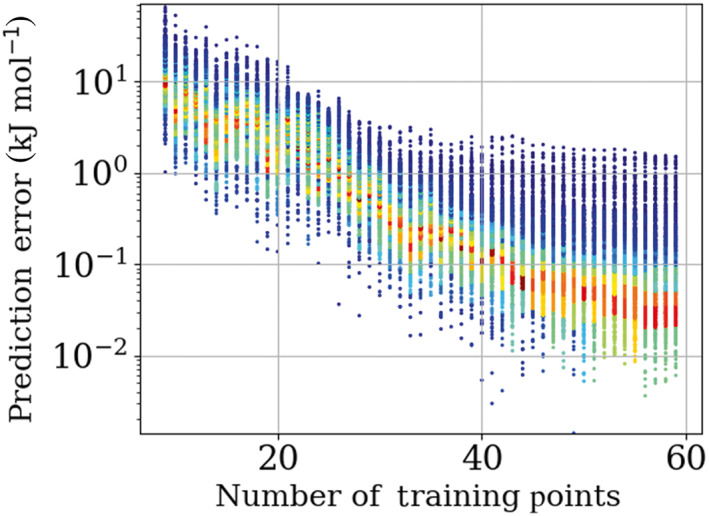
Prediction errors for a 3000 K water model produced using 1‐point‐per‐iteration per‐atom active learning. Blue indicates a low frequency of points for a given prediction error while red indicates a high frequency of points for a given prediction error with the familiar rainbow colors marking intermediate frequencies

The effectiveness of the active learning can be displayed by tracking the predictive performance of the models produced as the training set size increases. Tracking the predictive performance of the model can be achieved by producing a root‐mean‐square error (RMSE) plot where this error (for each model) is plotted against the number of training points for that model. However, an RMSE value is only part of the picture because a model may have a large RMSE value caused by only a few poor predictions. Conversely the model can produce a lower RMSE than is representative if the model predicts a particular area of the validation set quite well. To remedy this issue, plotting the whole spectrum of prediction errors against the number of training points shows the performance of each model against every point in the validation set. Figure 7 shows this type of plot for water trained at 3000 K.

**FIGURE 8:**
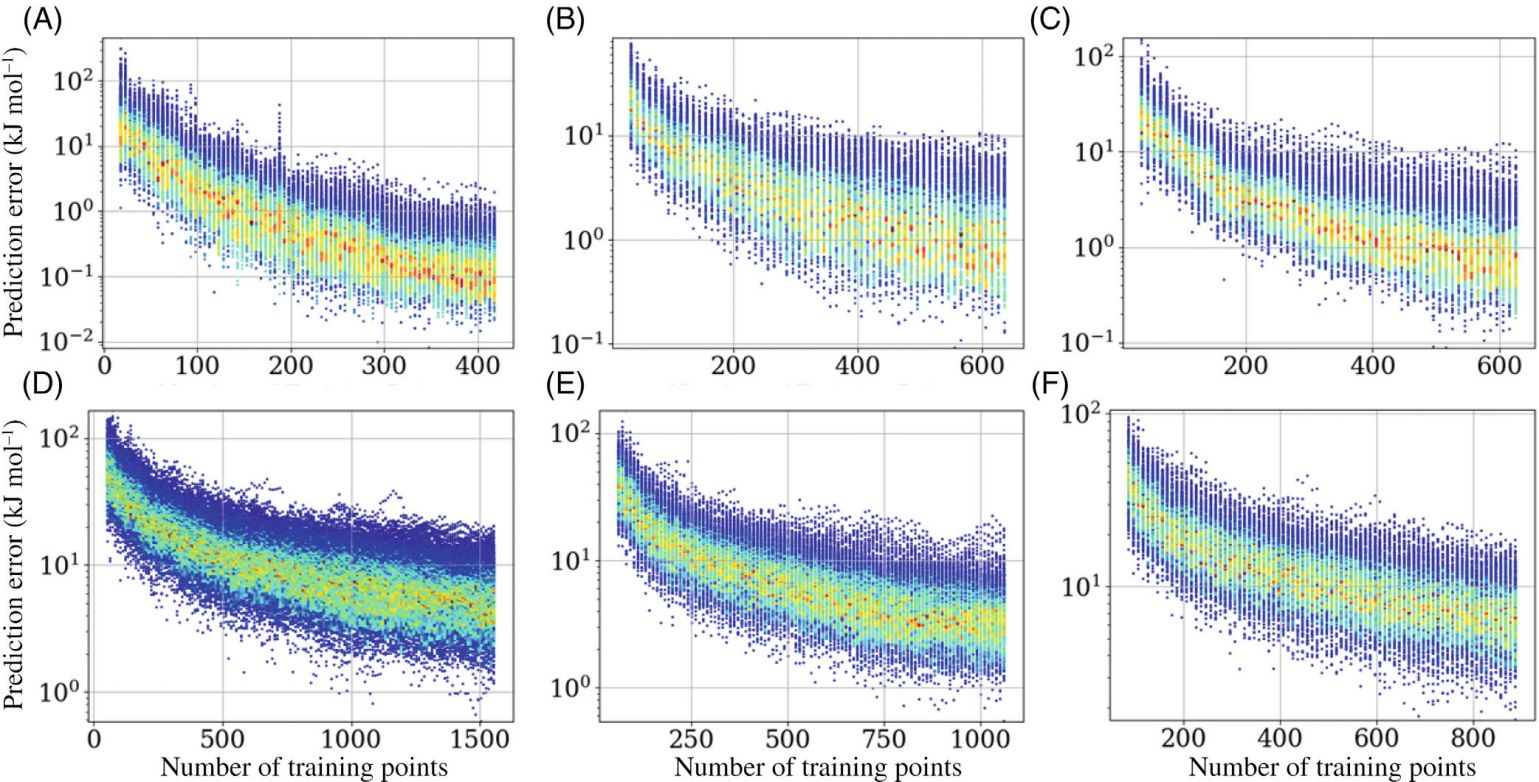
Prediction errors for (A) ammonia at 1000 K and 5‐points‐per‐iteration per‐atom, (B) methanol at 1000 K and 10‐points‐per‐iteration per‐atom, (C) formamide at 1000 K and 10 points‐per‐iteration per‐atom, (D) urea at 1000 K and 10 points‐per‐iteration per‐atom, (E) imidazole at 1000 K 10‐points‐per‐iteration per‐atom, and (F) *N*‐methylacetamide by the program TYCHE at 1750 K and 10‐points‐per‐iteration per‐atom

Increasing the number of training points for a given system decreases the prediction error on average. How well the active learning is able to perform this task depends on both the system and the temperature that the system was sampled at. Figure 8 demonstrates the improvement in both mean and maximum prediction errors across an active learning run for the remaining systems presented in each study.

As seen previously and in line with intuition, as the number of training points increases the prediction error decreases. The colored histogram shows that the average prediction error for each atom in the system consistently falls below the threshold of 1 kJ mol^−1^, with maximum (atomic) prediction errors never exceeding 2.4 kJ mol^−1^ for a given atom. It is important to not only look at the average prediction error but also the maximum and the spread of the prediction error. This is because a good average with a large maximum may be fine for most situations but can cause a simulation to fail if a geometry close to the maximum error is reached.

Prediction error plots for the rest of the systems discussed in this study can be found in Figures S27–S42. Some runs, such as formamide (1000 K) caution against the use of 1 ppi (per‐atom and per‐system, Figure S34A,B) due to occasionally poor maximum prediction errors. We note that the 10‐points‐per‐iteration does not suffer from this problem. On a related note, the 10‐points‐per‐iteration run for glycine (Figure S42) shows a very short error spike around 700 training points. This temporary setback is most likely due to poor models as a result of unsatisfactory hyperparameter optimization. However, the general robustness of many‐points‐per‐iteration active learning ensured a full recovery leading to ever decreasing errors with an increasing number of training points.

### 3.4 True versus predicted

Because the models produced by ICHOR and FEREBUS are designed to be used during a MD simulation the prediction accuracy of the model is only half the story; the other half is over what range of values the model can predict accurately. During a simulation, a large variety of configurations may be observed, and it is important that the model can predict the values over a large enough range for a given simulation.

**FIGURE 9:**
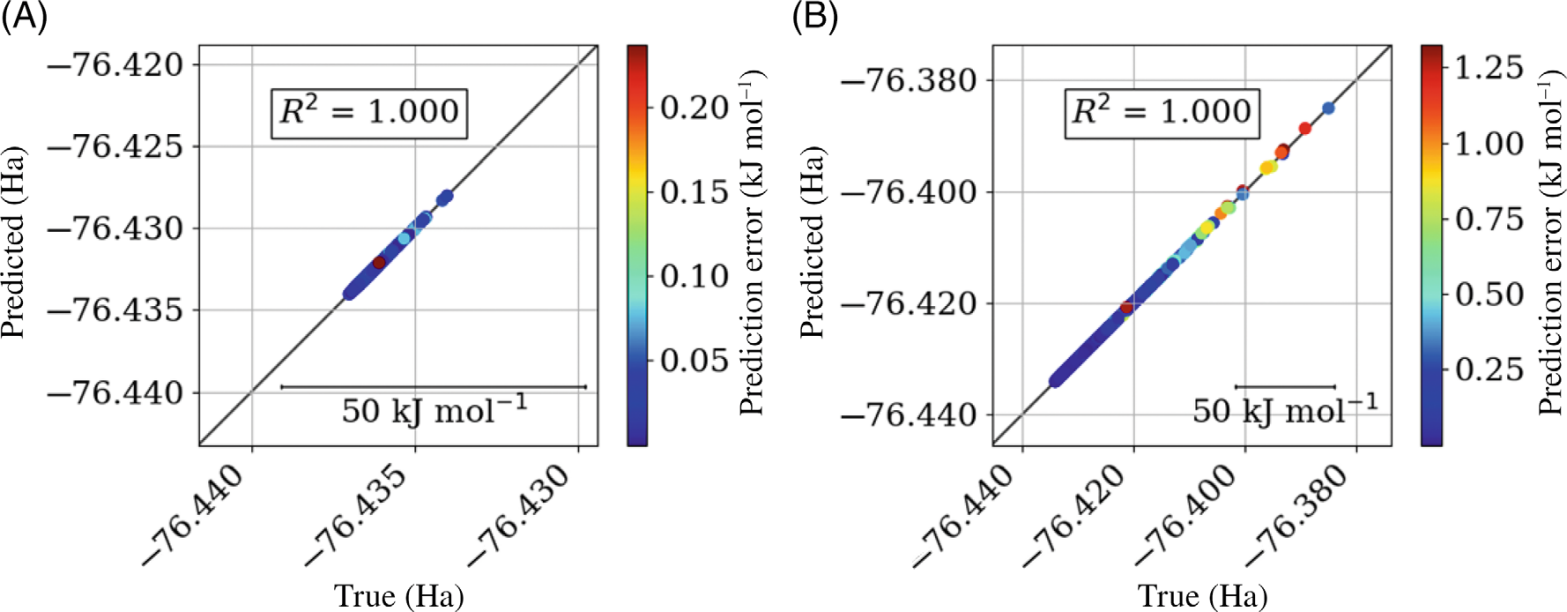
True versus predicted plots for water 1‐point‐per iteration per‐atom models at (A) 300 K and (B) 3000 K

Figure 9 shows that both models display excellent predictions but the 3000 K model does this over a much larger range of values than the 300 K model. The 3000 K model will therefore be much more useful during real world simulations. Overall, the models produced by active learning show excellent predictions across a large range of values.

**FIGURE 10:**
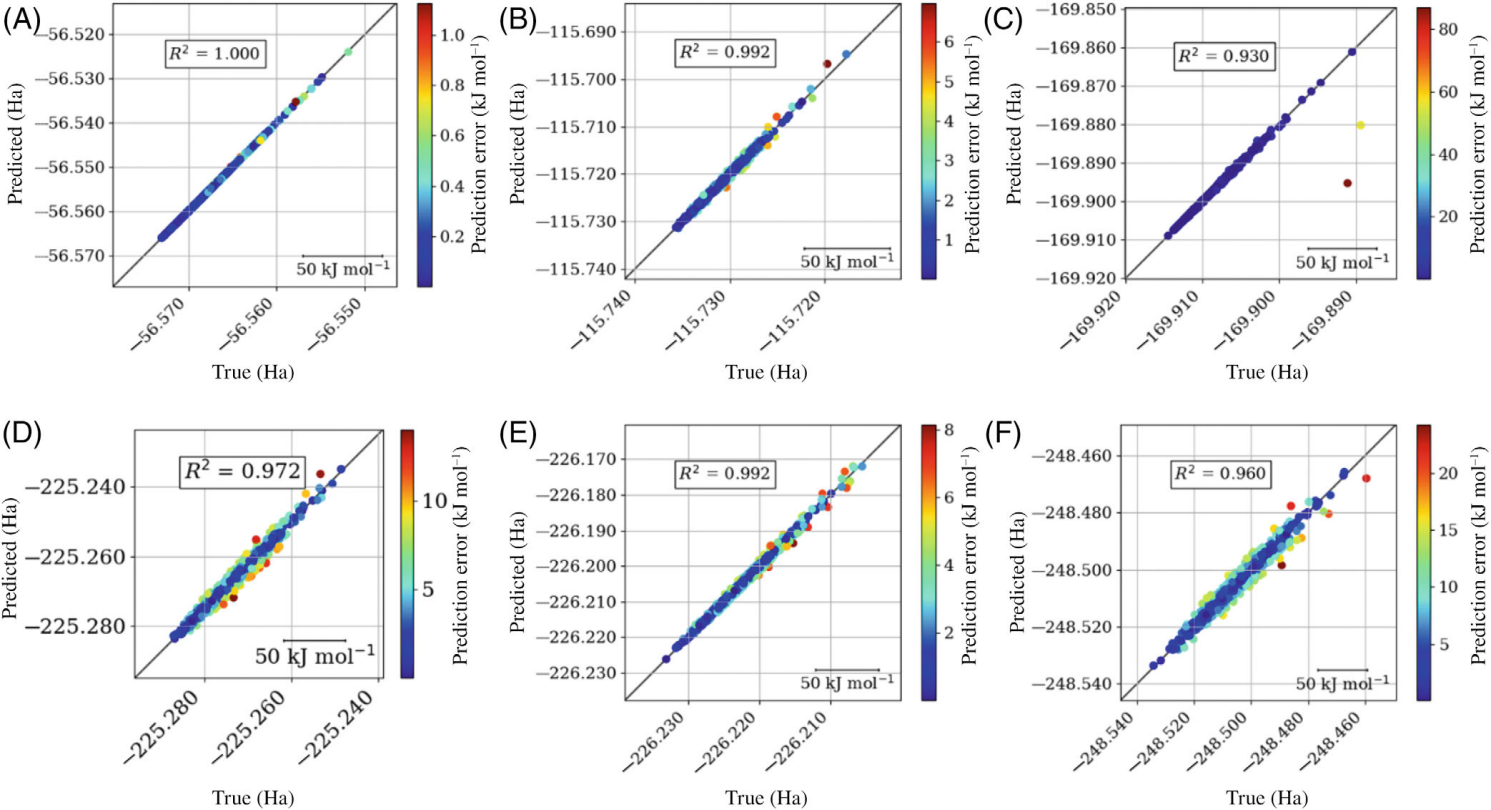
True versus predicted energy plots, all at 1000 K, for (A) ammonia 5‐points‐per‐iteration per‐atom, (B) methanol 10‐points‐per‐iteration per‐atom, (C) formamide 10‐points‐per‐iteration per‐atom, (D) urea 10‐points‐per‐iteration per‐atom, (E) imidazole 10‐points‐per‐iteration per‐atom, and (F) *N*‐methylacetamide 10‐points‐per‐iteration per‐atom

In general, the systems shown predict lower energy conformations better than higher energy conformations, which is demonstrated in Figure 10. This observation was to be expected because the initial sampling was performed using a fixed‐temperature MD simulation. As a result, there are fewer examples of higher energy configurations than there are of lower energy ones. Due to fewer high energy configurations, there will be fewer training points at higher energies and therefore higher prediction errors at these higher energy configurations. This issue is only prominent when the model is to be used during a high temperature simulation. In that case a higher initial temperature AIMD simulation would be required to sample more of the domain space.

### 3.5 Glycine

AIMD simulations are expensive to run and consequently cannot be run for a long simulation. The cost of AIMD simulations directly influences the model because there are not enough geometries to describe the dynamics of the initial system if the simulation has not run for long enough. For the glycine model AIMD was simply too expensive to run, which is why normal mode sampling was invoked, as implemented by the in‐house program TYCHE. Much like with AIMD sampling, TYCHE works off a fixed temperature allowing for fine control of the initial domain space while creating thousands of geometries in a fraction of the time of that associated with AIMD.

It has been observed in smaller systems that using multiple‐point‐per‐iteration active learning shows no significant decrease in the accuracy of the model. Similarly, the use of per‐atom sampling compared to per‐system also shows no decrease in performance. In certain situations, these two methods of active learning show an improvement in the accuracy of the models produced. When moving to larger systems, single‐point‐per‐iteration per‐system active learning becomes infeasible, and the speed of multiple‐point‐per‐iteration combined with per‐atom active learning is necessary to add enough points to the GPR model to achieve chemical accuracy. Therefore, from this point forward, we will only be using 10 ppi and per‐atom active learning.

A TYCHE temperature of 450 and 1750 K was chosen to create the initial domain spaces for the glycine model. We will focus on the results of the higher temperature sampling as this is the model that will be most useful in MD simulations. The results for the 450 K sampling can be found in Supporting Information S1.

**FIGURE 11:**
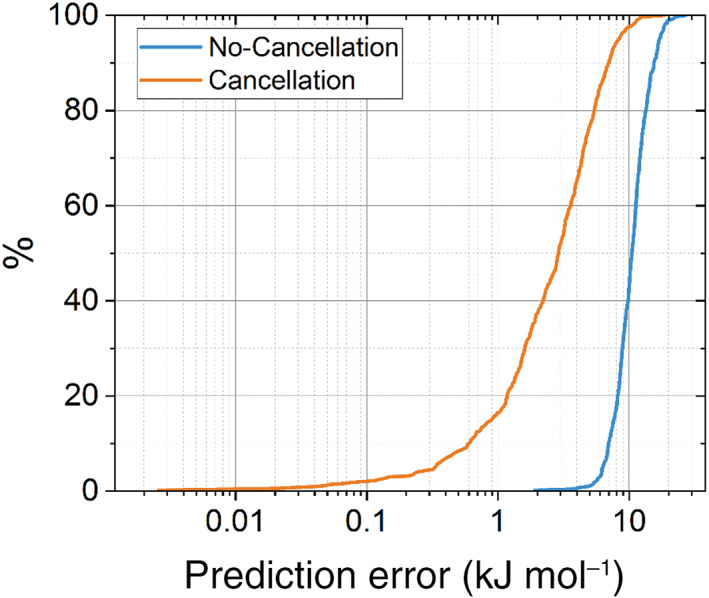
S‐curve for peptide‐capped glycine (1700 training points) for both prediction error calculations with and without cancellation of errors

Figure 11 shows the S‐curve (no‐cancellation) for peptide‐capped glycine where just over 40% of the test configurations have an error of less than 10 kJ mol^−1^ while the maximum error occurs at 27 kJ mol^−1^. The 1700‐point glycine model reaches an average error of only 0.57 kJ mol^−1^ for a given atom, calculated by taking the mean of the prediction errors from the validation set and dividing by the number of atoms in peptide‐capped glycine. As these are absolute prediction errors, the true total prediction error during a simulation will likely be lower than these values due to favorable cancellation of errors. The second S‐curve (“cancellation”) shows this type of error (which is closer to the physics of energy because atomic contributions are indeed added without involving absolute values). Now about two thirds of the errors are below 1 kcal mol^−1^.

Both the prediction error plot (Figure 12) and true versus predicted plot (Figure 13) follow the same trend as seen for the smaller systems. This is encouraging as it paves the way for larger systems with increasing biological interest. Glycine displays excellent predictions with relatively few training points across the reasonable sized energy range of 377 kJ mol^−1^ for TYCHE 1750 K. Figures S43–S58 exhaustively report on true versus predicted relationships for all eight systems.

**FIGURE 12:**
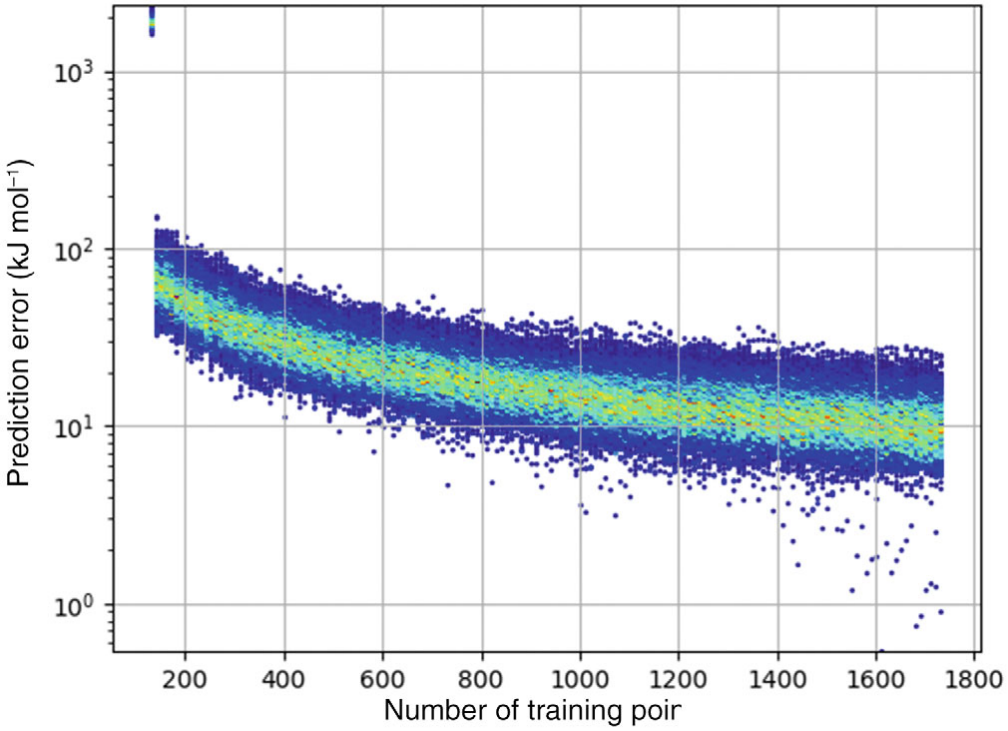
Energy prediction error plots for glycine. It is observed that the first three models of the active learning run produced significantly worse errors hence the discontinuity

**FIGURE 13:**
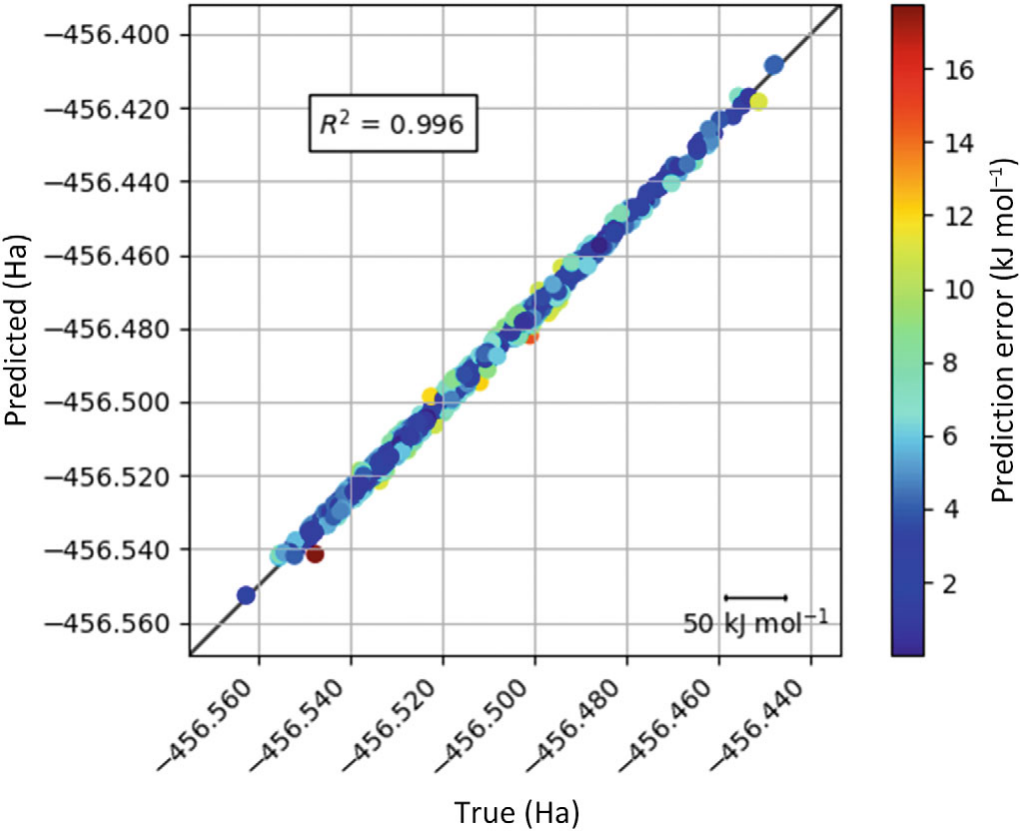
True versus predicted plots for glycine (1700 training points)

## 4 CONCLUSIONS

This work reports on an important step in producing accurate and reliable GPR models for MD simulations in the force field FFLUX. A range of molecules has been presented, each an important stepping stone toward producing accurate models for simulating biomolecules.

Per‐atom and multiple‐point addition active learning are two advancements that have made the production of models for larger systems possible while maintaining the accuracy required for reliable simulations. The per‐atom approach coupled with multiple‐point addition active learning allows for an asynchronous active learning pipeline resulting in models that are as accurate or better than the equivalent per‐system single‐point addition produced in a fraction of the CPU time. Decreasing the amount of time taken to produce a model using active learning allows for more points to be added to the model leading to more accurate models.

Moving toward larger and larger systems results in AIMD sampling becoming infeasible resulting in the necessity to move toward other sampling techniques. Normal mode sampling was demonstrated here as a fast alternative but other sampling techniques such as classical MD simulations could also be used in the future.

Accurately predicting atomic energies is a major stepping stone in producing models for atomistic simulations but the method shown is not limited to a single property and can be extended to be used to produce models for any atomic property such as multipole moments and dispersion energies. Future work will detail the efficacy of this method to atomic multipole moments, which will unlock the ability to perform chemically accurate atomistic simulations with GPR models.

## Supporting information


**APPENDIX S1** Supporting information

## Data Availability

The data that support the findings of this study are available from the corresponding author upon reasonable request.
