# Supplementary material for: Producing chemically accurate atomic Gaussian process regression models by active learning for molecular simulation
Source: J Comput Chem. 2022 Sep 27;43(31):2084–98. doi: 10.1002/jcc.27006 (PMC9828508; doi:10.1002/jcc.27006)
Supplement: Supplementary file 1 — APPENDIX S1 Supporting information [file JCC-43-2084-s001.docx]

**Supporting Information**

**Producing Chemically Accurate Atomic Gaussian Process Regression Models By Active Learning For** **Molecular Simulation**

Matthew Burn^1,2^ and Paul Popelier^1,2^ *

^1^ Manchester Institute of Biotechnology, The University of Manchester, Manchester, M1 7DN, Britain

^2^ Department of Chemistry, The University of Manchester, Manchester, M13 9PL, Britain

*To whom correspondence should be addressed:

Phone: +44 161 3064511. E-mail: [pla@manchester.ac.uk](mailto:pla@manchester.ac.uk)

Table of Contents

[1 ICHOR 3](#_Toc110848630)

[2 Feature Calculation 4](#_Toc110848631)

[3 Atom Numbering 5](#_Toc110848632)

[3.1 Water 5](#_Toc110848633)

[3.2 Ammonia 5](#_Toc110848634)

[3.3 Methanol 6](#_Toc110848635)

[3.4 Formamide 6](#_Toc110848636)

[3.5 Urea 7](#_Toc110848637)

[3.6 Imidazole 7](#_Toc110848638)

[3.7 NMA 8](#_Toc110848639)

[3.8 Glycine 8](#_Toc110848640)

[4 Mist Plots 9](#_Toc110848641)

[4.1 Kabsch Algorithm 9](#_Toc110848642)

[4.2 Water 10](#_Toc110848643)

[4.3 Ammonia 10](#_Toc110848644)

[4.4 Methanol 11](#_Toc110848645)

[4.5 Formamide 11](#_Toc110848646)

[4.6 Urea 12](#_Toc110848647)

[4.7 Imidazole 12](#_Toc110848648)

[4.8 NMA 13](#_Toc110848649)

[4.9 Glycine 13](#_Toc110848650)

[5 S-Curves 14](#_Toc110848651)

[5.1 Water 14](#_Toc110848652)

[5.2 Ammonia 14](#_Toc110848653)

[5.3 Methanol 15](#_Toc110848654)

[5.4 Formamide 15](#_Toc110848655)

[5.5 Urea 16](#_Toc110848656)

[5.6 Imidazole 16](#_Toc110848657)

[5.7 NMA 17](#_Toc110848658)

[5.8 Glycine 17](#_Toc110848659)

[6 Prediction Error Progression 18](#_Toc110848660)

[6.1 Water 18](#_Toc110848661)

[6.2 Ammonia 20](#_Toc110848662)

[6.3 Methanol 22](#_Toc110848663)

[6.4 Formamide 24](#_Toc110848664)

[6.5 Urea 26](#_Toc110848665)

[6.6 Imidazole 28](#_Toc110848666)

[6.7 NMA 30](#_Toc110848667)

[6.8 Glycine 33](#_Toc110848668)

[7 True vs Predicted 34](#_Toc110848669)

[7.1 Water 34](#_Toc110848670)

[7.2 Ammonia 36](#_Toc110848671)

[7.3 Methanol 38](#_Toc110848672)

[7.4 Formamide 40](#_Toc110848673)

[7.5 Urea 42](#_Toc110848674)

[7.6 Imidazole 44](#_Toc110848675)

[7.7 NMA 46](#_Toc110848676)

[7.8 Glycine 49](#_Toc110848677)

# ICHOR

ICHOR is a pipeline application for producing Gaussian process regression (GPR) models for atomistic simulations in the program DL_FFLUX. ICHOR is responsible for interfacing with quantum mechanics programs, machine learning programs and molecular dynamics programs. ICHOR produces training sets and carries out model analysis and active learning. In order to perform these tasks effectively, ICHOR makes full use of modern HPC clusters, which enable running many jobs in parallel. This capacity greatly increases the throughput of model creation. Figure S1 shows a basic ICHOR pipeline.


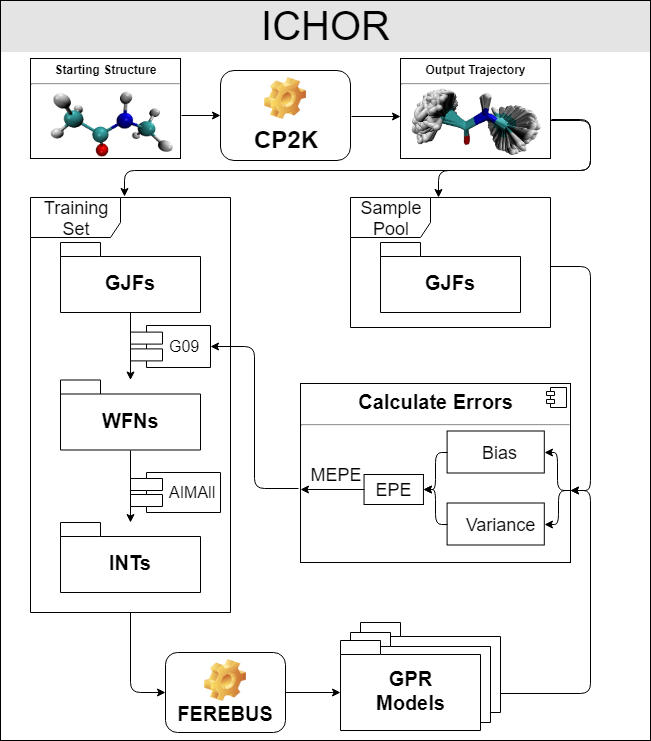


Figure S1. The basic ICHOR pipeline. The names of the programs and most abbreviations can be found in the main text while WFN stands for wavefunction file and GJF for Gaussian job file.

When running ICHOR in per-atom mode, the atomic properties calculated by AIMAll (and therefore the models produced by FEREBUS) are for a single atom only, for each instance (occurrence) of ICHOR. ICHOR is able to run multiple instances of itself in parallel to not only reduce the time penalty for more computation but in fact significantly speed up the production of models due to the asynchronous (i.e. not having to wait for a previous command to finish) nature of the task scheduling in per-atom mode.

# Feature Calculation

The ICHOR pipeline uses geometric features calculated using an atomic local frame (ALF). The ALF is defined using the Cahn-Ingold-Prelog rules for setting the priority of each atom connected to the origin atom $\boldsymbol{A}$**.** Using these priorities, the atom defining the x-axis ($\boldsymbol{A}_{x}$) is set to the atom with highest priority, and the atom defining the xy-plane ($\boldsymbol{A}_{xy}$) is set to the atom with the second highest priority.


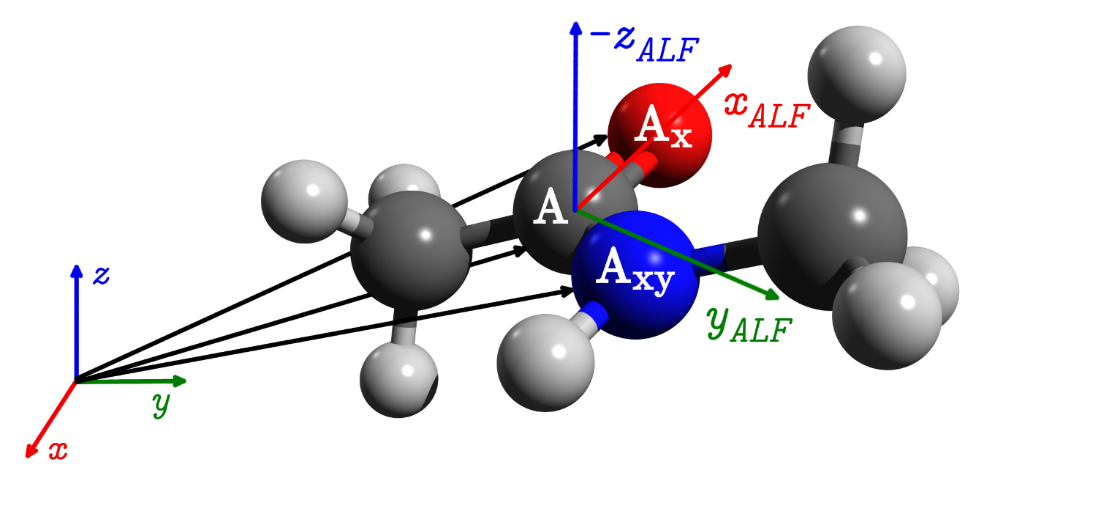


Figure S2. The definition of an ALF for the central carbon atom in NMA.

Once the atomic local frame is defined, the geometric features for each atom relative to the ALF can be calculated using equations S1-S6:

|  | $A_{x}=\sqrt{\left( \boldsymbol{A}_{x}-\boldsymbol{A} \right)^{2}}$ | (S1) |
| --- | --- | --- |
|  | $A_{xy}=\sqrt{\left( \boldsymbol{A}_{xy}-\boldsymbol{A} \right)^{2}}$ | (S2) |
|  | $\chi^{A}=\cos^{-1}\left( \frac{\boldsymbol{A}_{x}.\boldsymbol{A}_{xy}}{A_{x}A_{xy}} \right)$ | (S3) |
|  | $A_{n}=\sqrt{\left( \boldsymbol{A}_{n}-\boldsymbol{A} \right)^{2}}$ | (S4) |
|  | $\theta^{An}=\cos^{-1}\left( \frac{\zeta_{3}^{\boldsymbol{A}_{n}}}{A_{n}} \right)$ | (S5) |
|  | $\phi^{An}=\tan^{-1}\left( \frac{\zeta_{2}^{\boldsymbol{A}_{n}}}{\zeta_{1}^{\boldsymbol{A}_{n}}} \right)$ | (S6) |
|  | $n=\left( 4,\ldots,N \right)$ |  |

The first three features are those related to the atoms in the ALF: (i) the distance from the origin atom to the atom defining the x-axis, (ii) the distance from the origin atom to the atom defining the xy-plane, and (iii) the angle between these two vectors. Beyond these first three features, each subsequent set of 3 features is the polar coordinates for atom *n* relative to the local axis system associated with the ALF. A full derivation^1^ of the feature calculation is given by Mills and Popelier.

# Atom Numbering

## Water


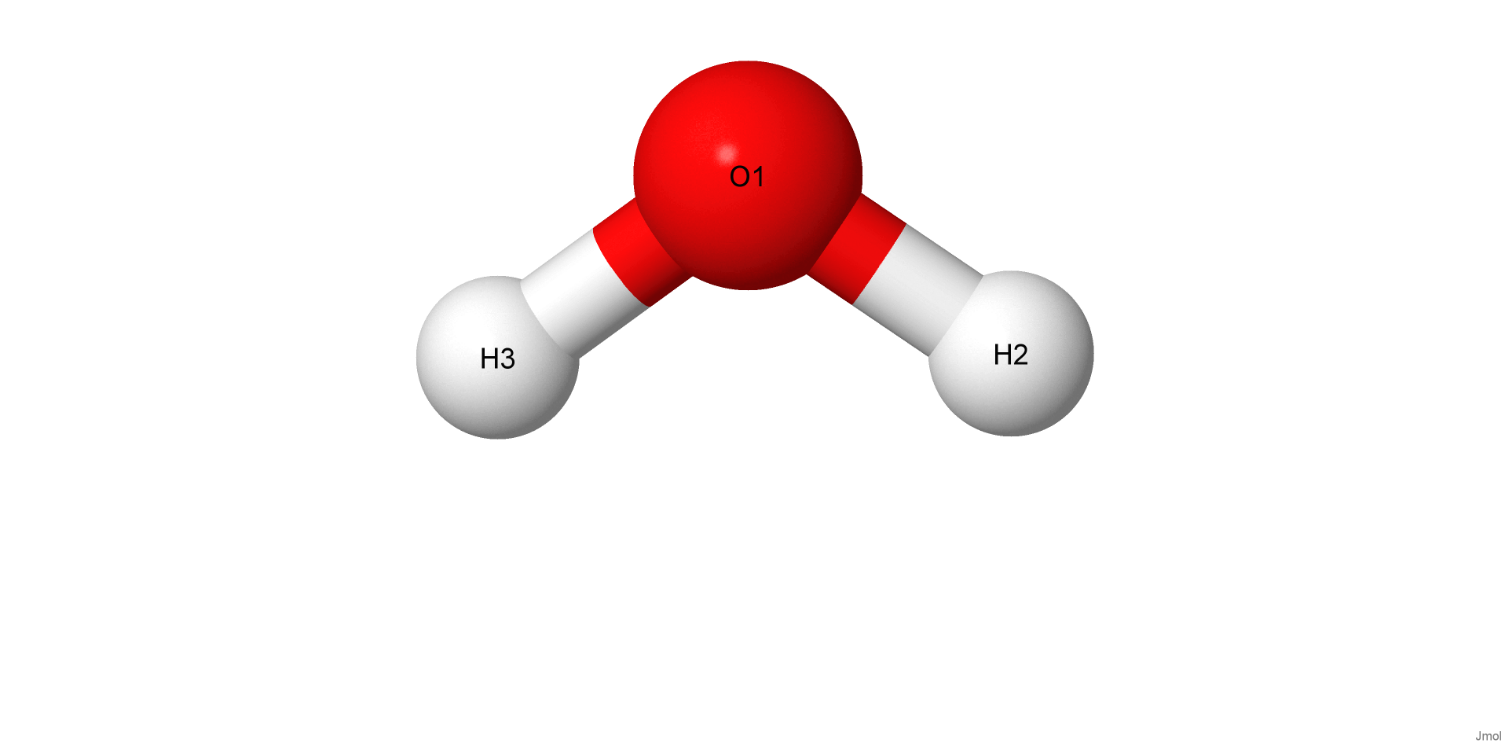


Figure S3. Atom labelling used for water models

## Ammonia


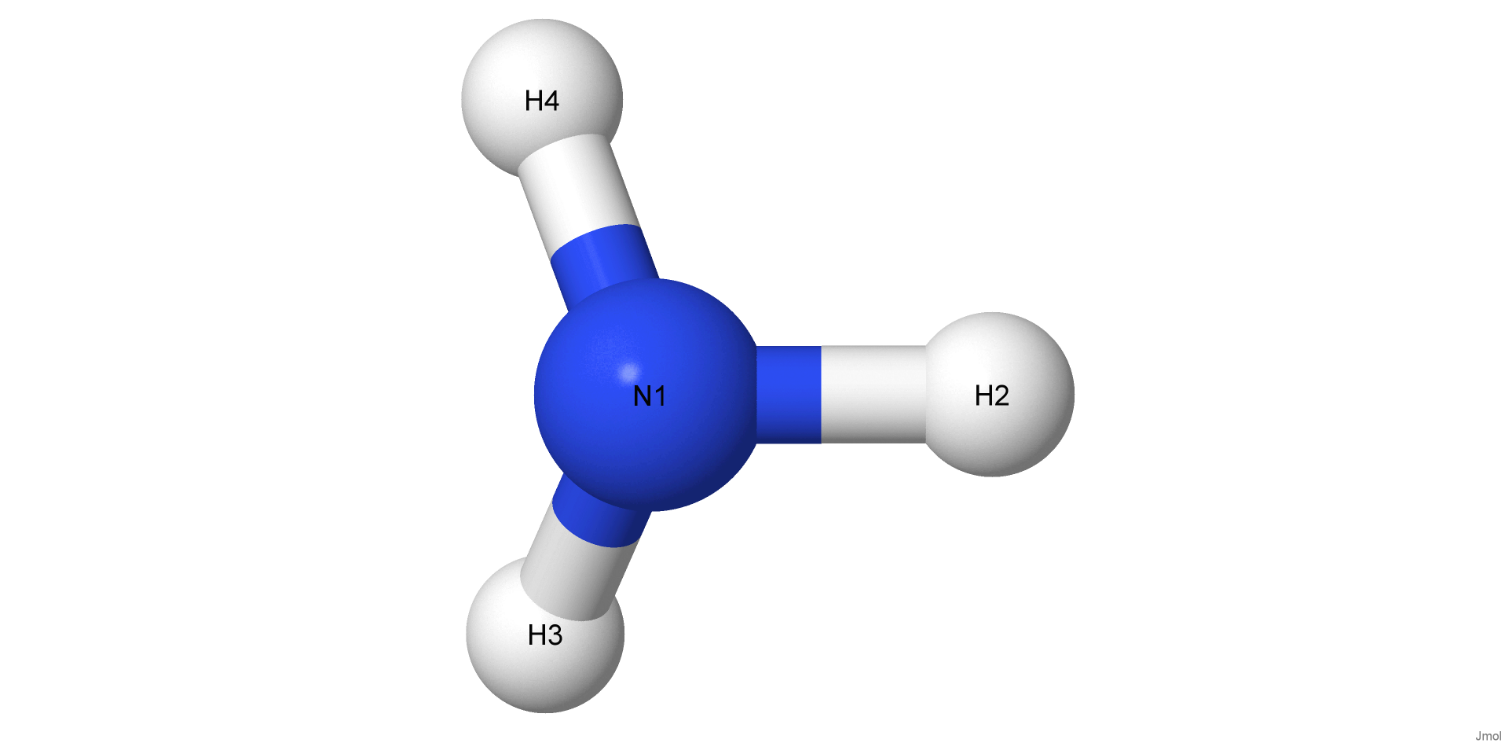


Figure S4. Atom labelling used for ammonia models

## Methanol


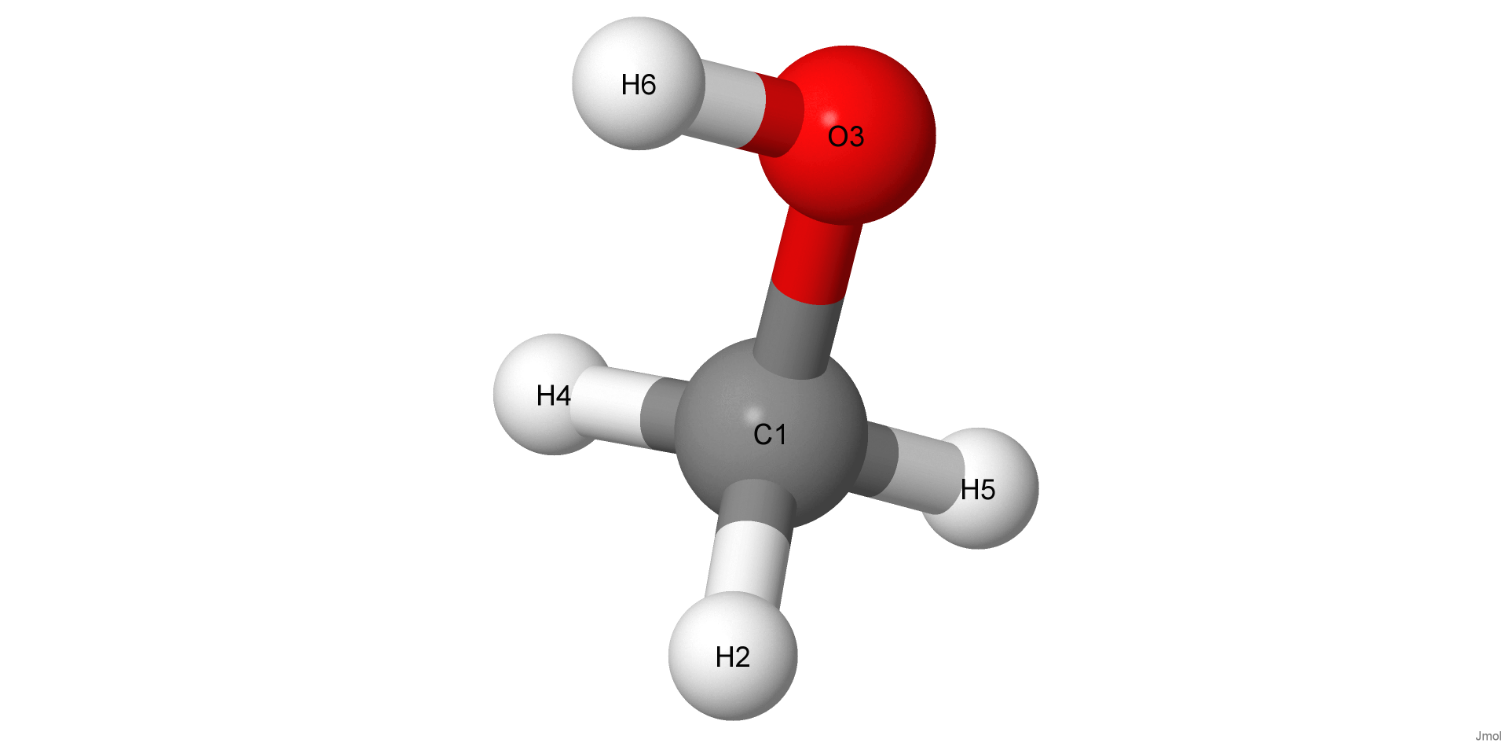


Figure S5. Atom labelling used for methanol models

## Formamide


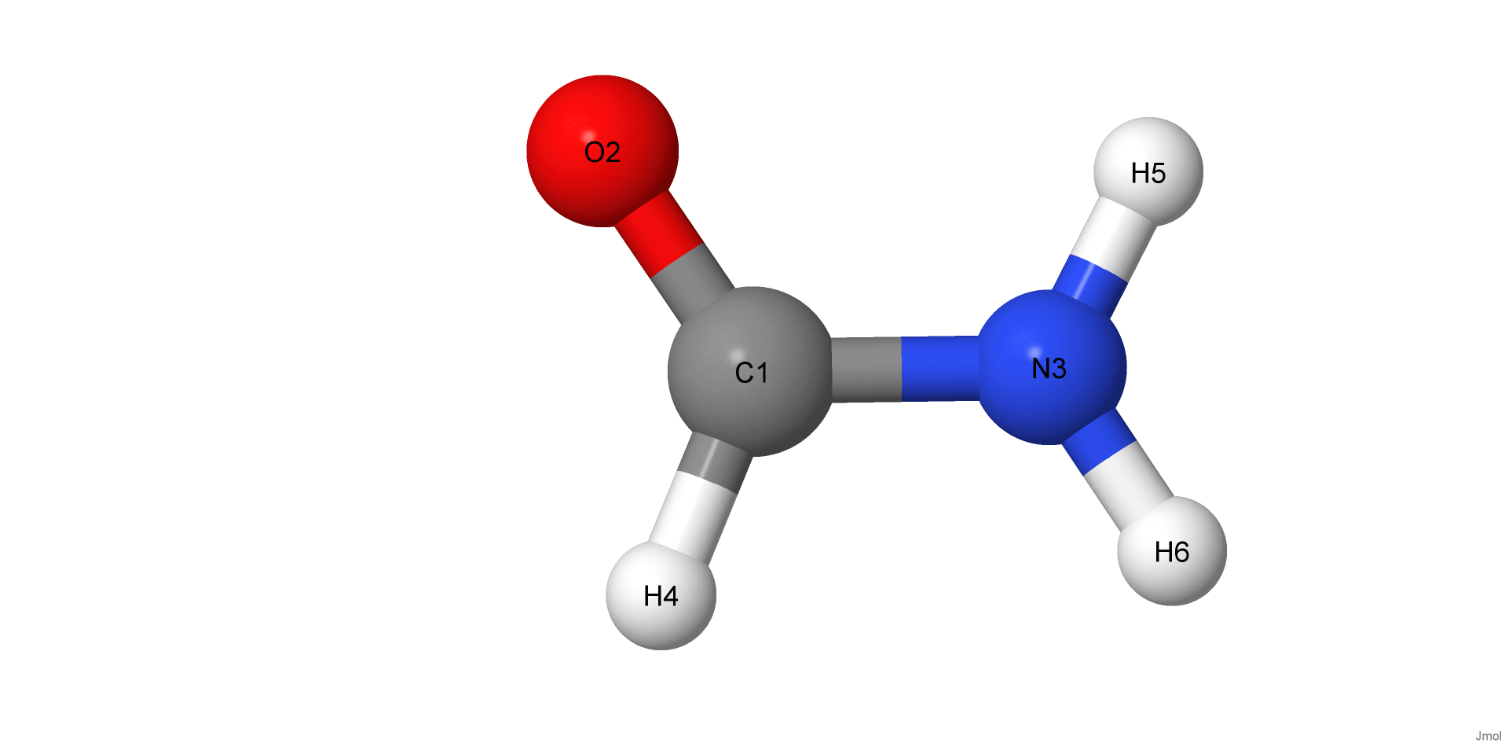


Figure S6. Atom labelling used for formamide models

## Urea


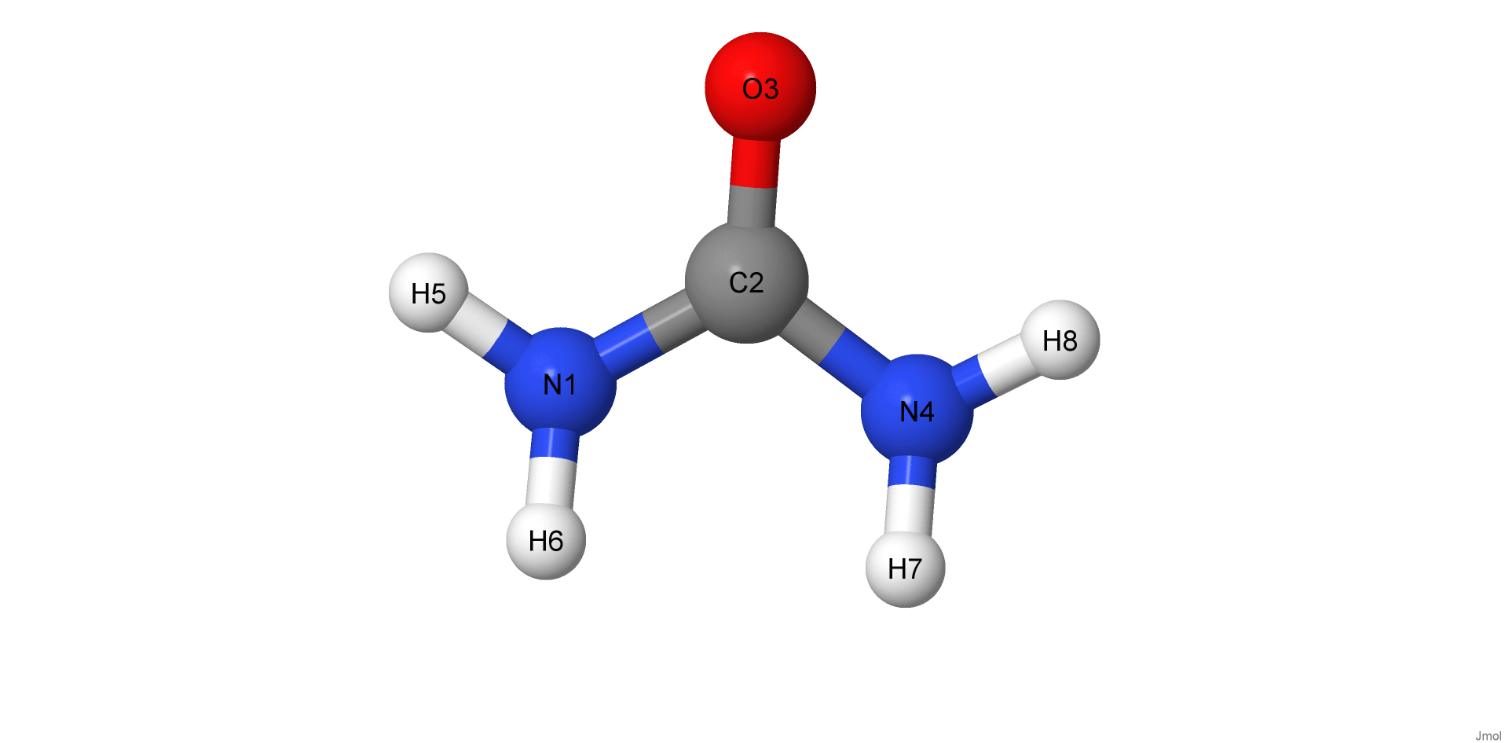


Figure S7. Atom labelling used for urea models

## Imidazole


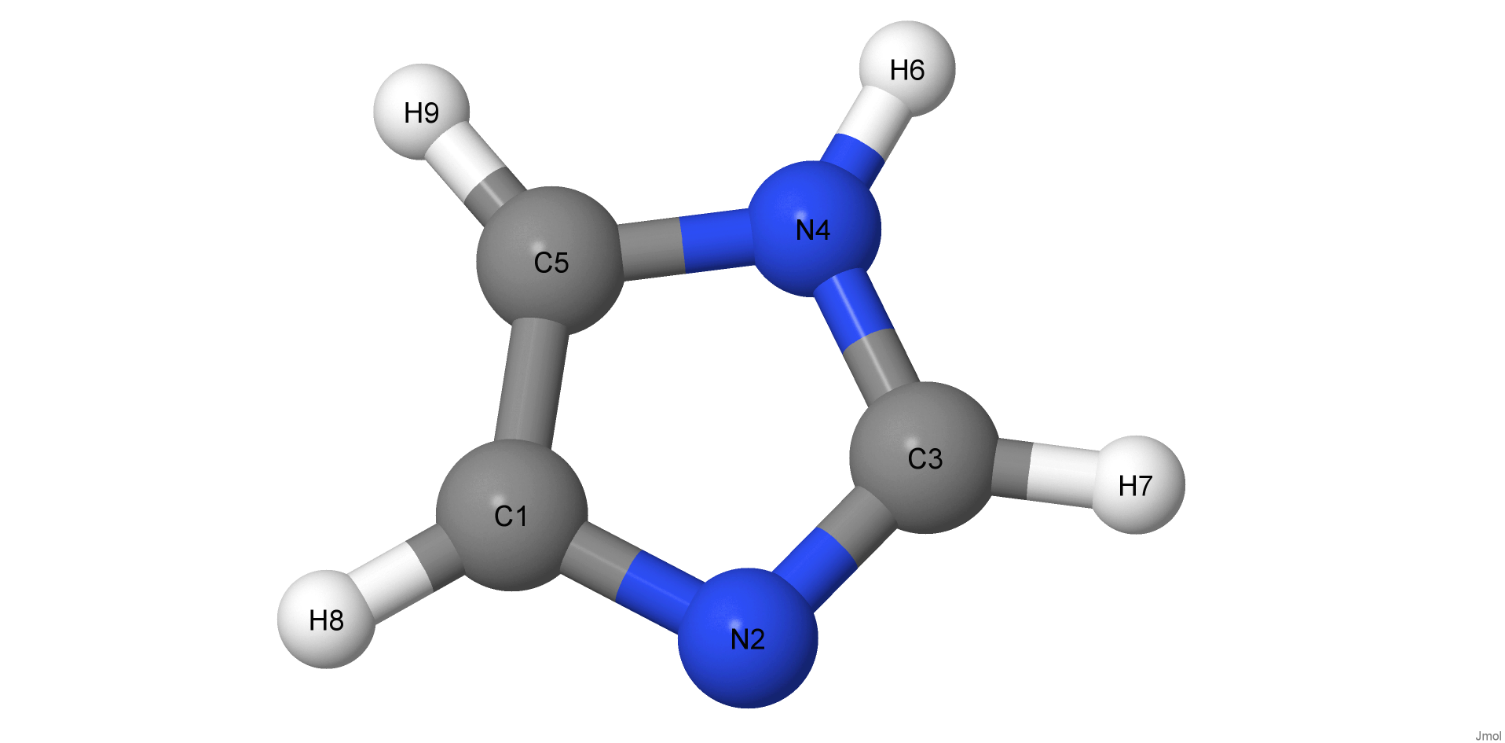


Figure S8. Atom labelling used for imidazole models

## NMA


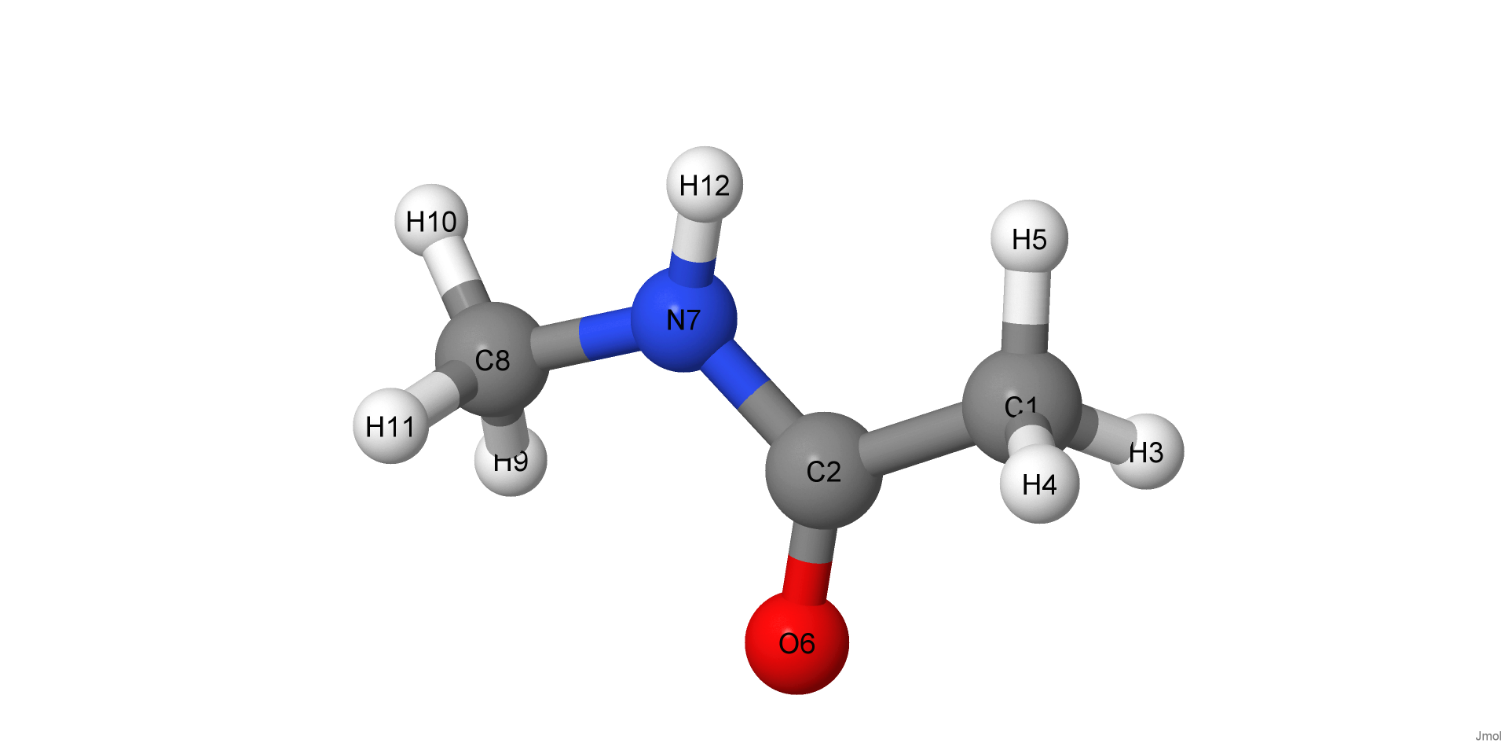


Figure S9. Atom labelling used for NMA models

## Glycine


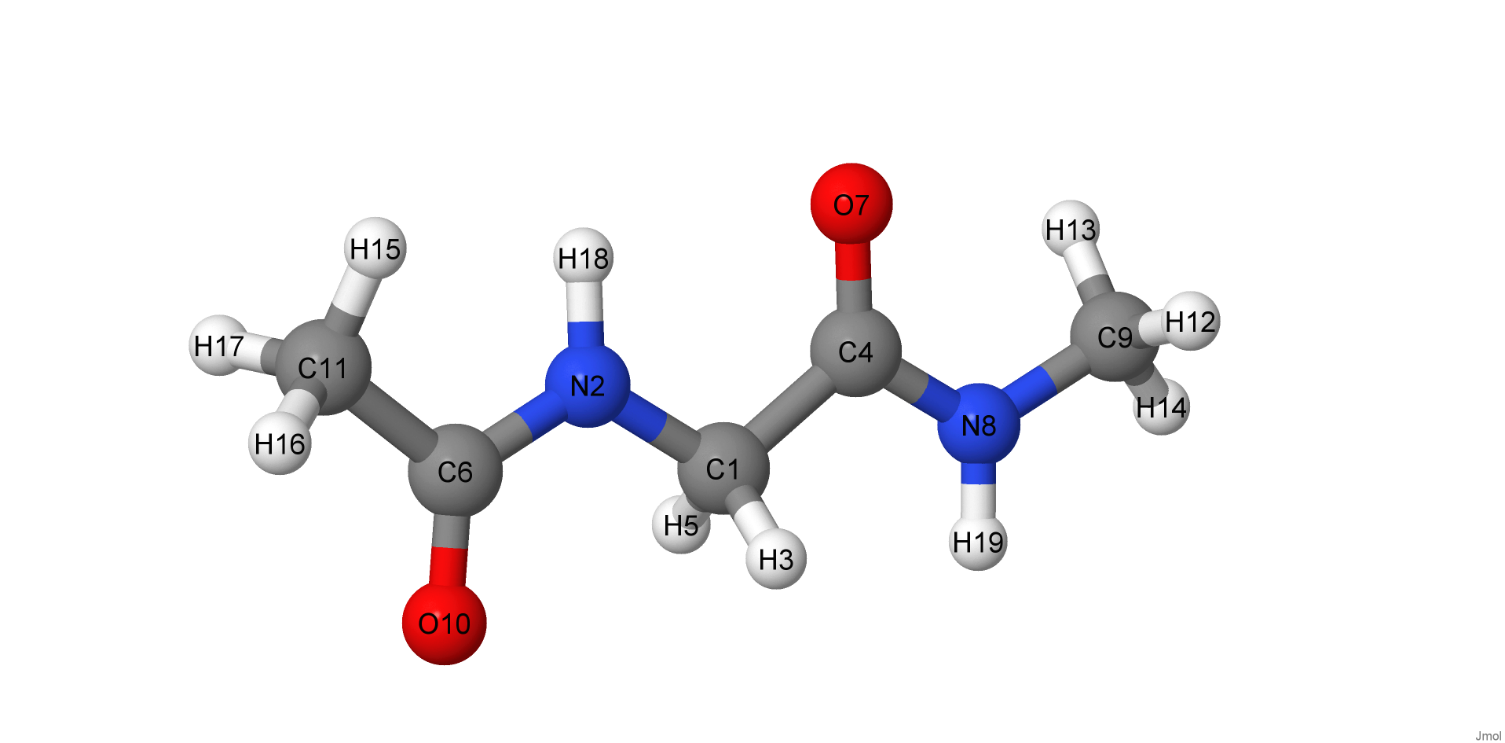


Figure S10. Atom labelling used for glycine models

# Mist Plots

## Kabsch Algorithm

To generate the mist plots, the configuration of each timestep in a trajectory needs to be overlapped as best as possible with the first time step of the trajectory. As a result, when plotting all timesteps as points, the ‘cloud’ of points show how the molecule has been distorted over the sampling run. To achieve this goal, the Kabsch algorithm is employed, which calculates the rotation matrix that minimises the RMSD between two sets of coordinates, each set describing a configuration.

The Kabsch algorithm requires that both sets of coordinates are centred on the origin, which is achieved by subtracting the centroid (the average of all atomic coordinates) from each coordinate. Given two sets of points $P$ and $Q$, each of which an $N\times3$ matrix of coordinate positions, the cross covariance matrix, $H$, is calculated using the following equation,

|  | $H_{ij}=\sum_{k=1}^{N} P_{ki}Q_{kj}$ | (S7) |
| --- | --- | --- |

The cross covariance matrix can then be factorised using singular value decomposition,

|  | $H=U\Sigma V^{T}$ | (S8) |
| --- | --- | --- |

The factorised matrix can then be used to determine the “handedness” of the rotation ($d$) and to calculate the rotation matrix $R$ itself,

|  | $d=\mathrm{sign}\left( \det\left( VU^{T} \right) \right)$ | (S9) |
| --- | --- | --- |
|  | $R=V\left( \begin{matrix} 1 & 0 & 0 \\ 0 & 1 & 0 \\ 0 & 0 & d \end{matrix} \right)U^{T}$ | (S10) |

Finally, points $P$ are rotated onto $Q$ using the previously calculated rotation matrix,

|  | $P_{rot}=\left( RP^{T} \right)^{T}$ | (S11) |
| --- | --- | --- |

The new points $P_{rot}$ have been rotated such that they are as overlapped as possible onto points $Q$ and are ready to be used in the mist plot.

Unfortunately, when visualising molecular movements, it may not be desirable to minimise the RMSD between two whole molecules as there may be molecular distortions such as methyl rotations that are less important than others. In such cases, a subsystem of the original system may be used to calculate the optimal rotation matrix allowing for good overlap of the atoms that need to be inspected whilst disregarding those atoms not of chemical interest. Note that the subsystem can be smaller than the whole system (see Table S1).

Once the geometries have been rotated, the system is then centred on, or between, a set of atoms so that the distortions can be viewed more easily. It is the user’s choice which subsystem to use during the rotation and which atoms to centre on. It may be preferrable to produce multiple plots for a single system in order to view different distortions.

Table S1. Subsystem and atom centering used whilst generating the mist plots. Atom numbering corresponds to those shown in Section 3 of the Supporting Information.

| **System** | **Subsystem** | **Atom Centring** |
| --- | --- | --- |
| **Water** | O1, H2, H3 | O1 |
| **Ammonia** | N1, H2, H3, H4 | N1 |
| **Methanol** | C1, H2, O3, H4, H5 | C1 |
| **Formamide** | C1, O2, N3, H4, H5, H6 | C1, N3 |
| **Urea** | N1, C2, O3, N4 | C2 |
| **Imidazole** | C1, N2, C3, N4, C5 | C1, N2, C3, N4, C5 |
| **NMA** | C1, C2, O6, N7, C8, H12 | C2, N7 |
| **Glycine** | C1, N2, C4, C6, O7, N8, C9, O10, C11, H18, H19 | C1 |

## Water


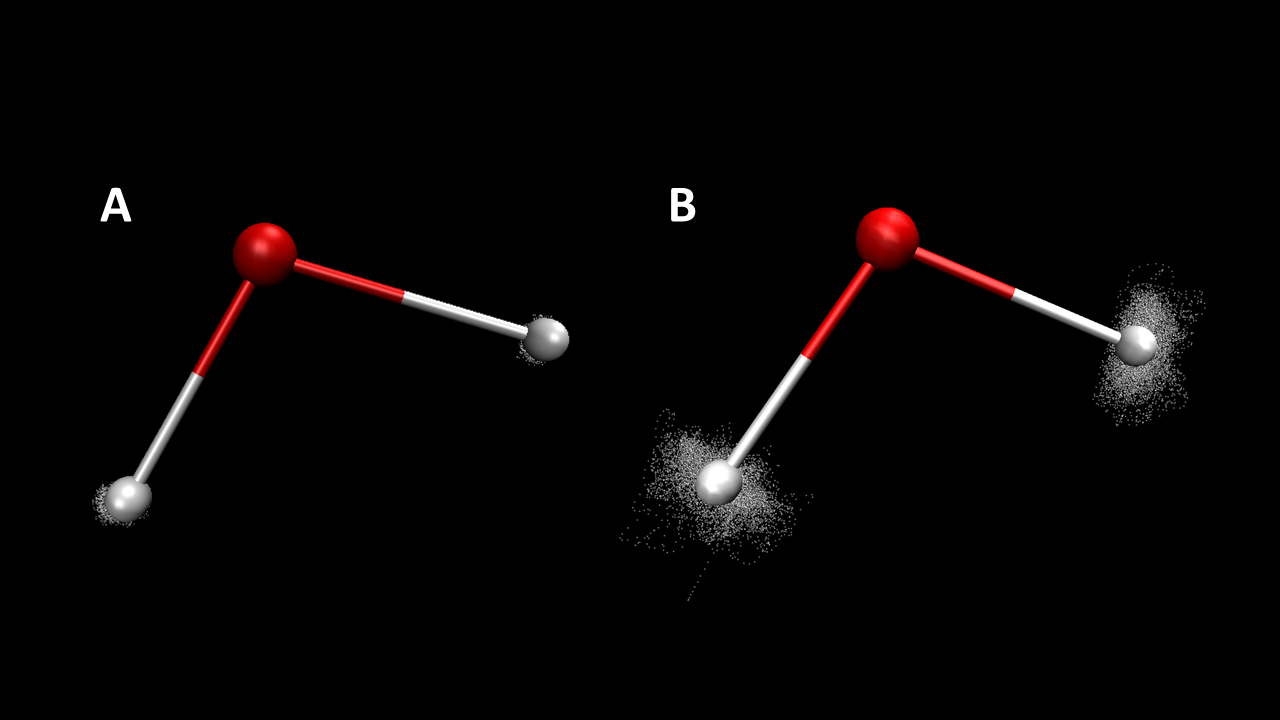


Figure S11. Mist plot for a) water 300 K b) water 3000 K

## Ammonia


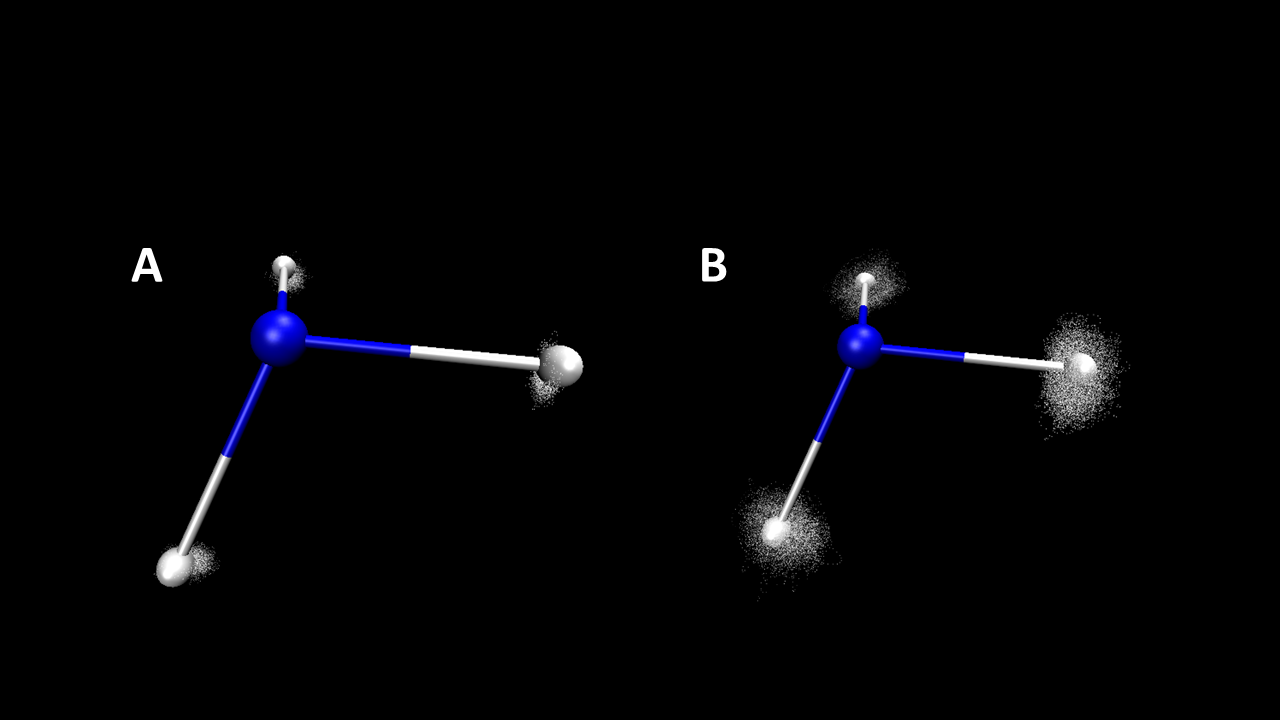


Figure S12. Mist plot for a) ammonia 300 K b) ammonia 1000 K

## Methanol


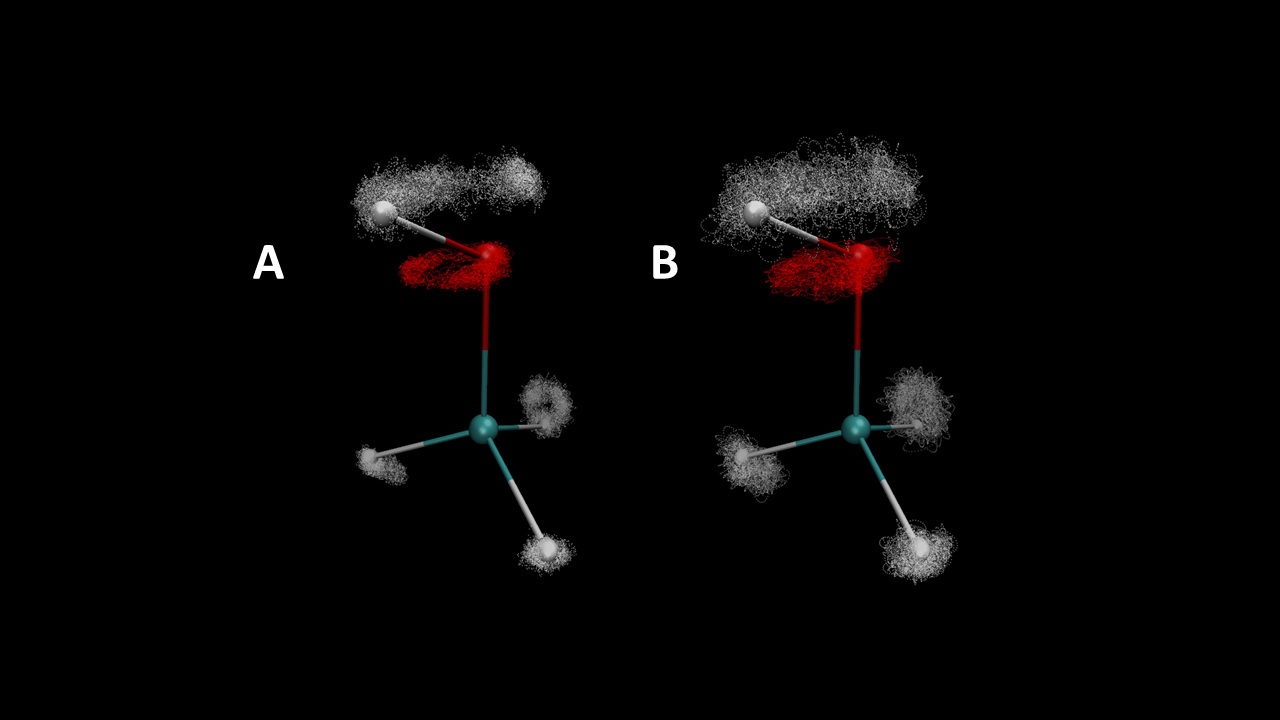


Figure S13. Mist plot for a) methanol 300 K b) methanol 1000 K

## Formamide


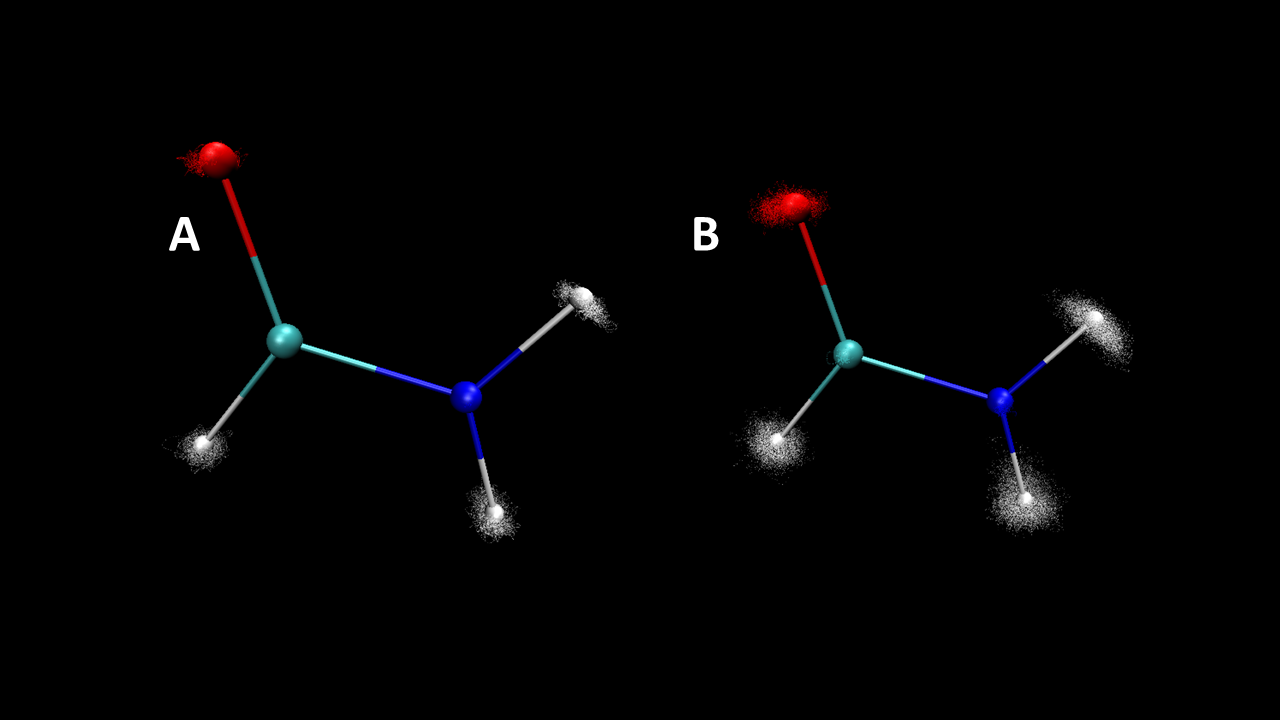


Figure S14. Mist plot for a) formamide 300 K b) formamide 1000 K

## Urea


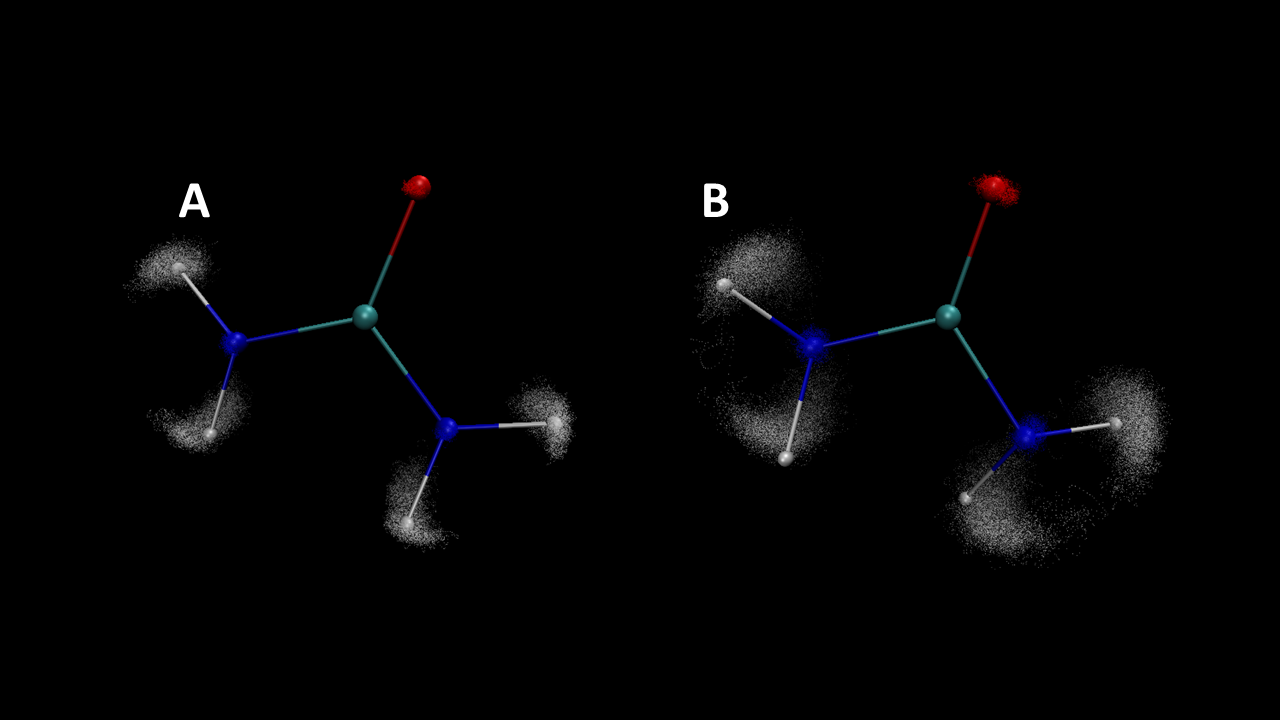


Figure S15. Mist plot for a) urea 300 K b) urea 1000 K

## Imidazole


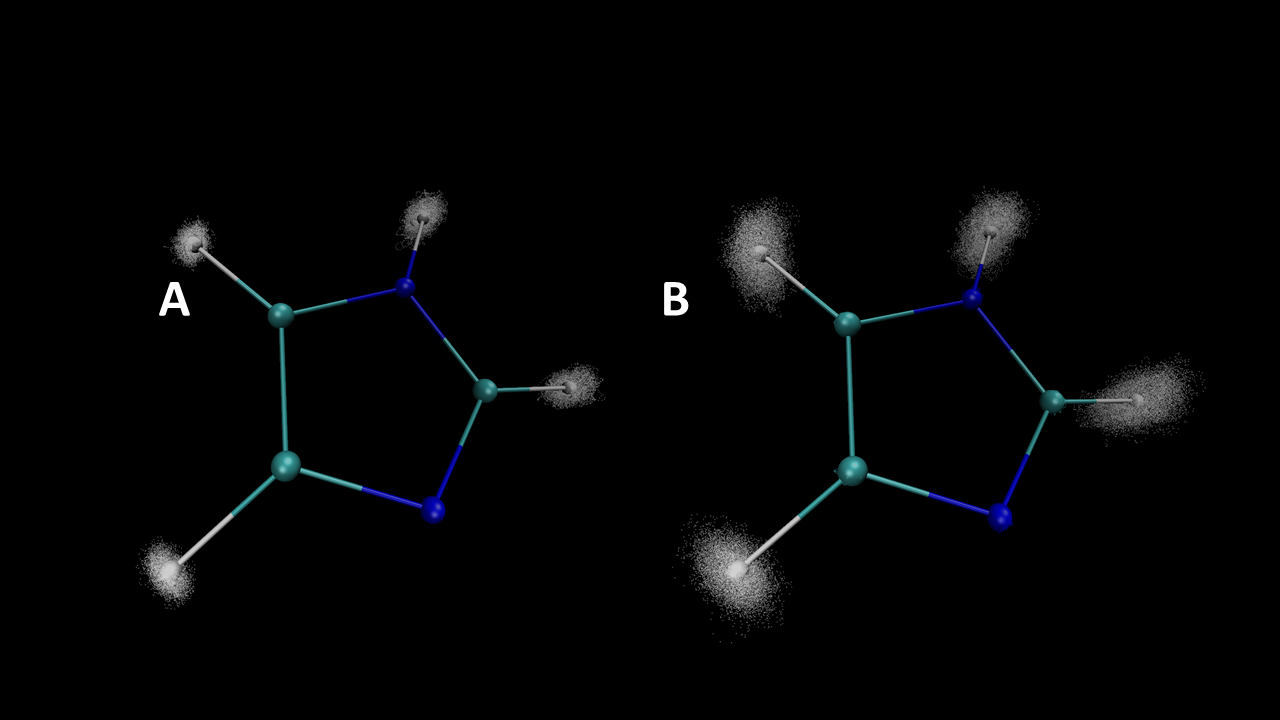


Figure S16. Mist plot for a) imidazole 300 K b) imidazole 1000 K

## NMA


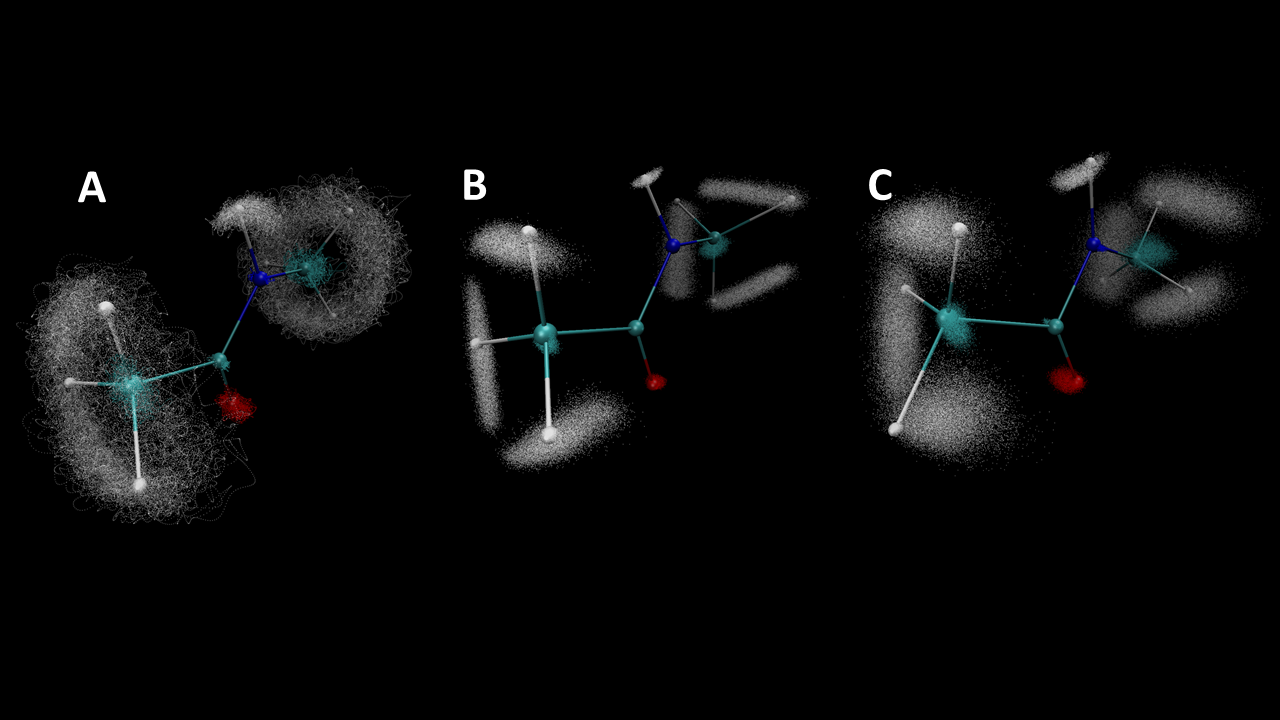


Figure S17. Mist plot for a) NMA 1000 K b) NMA Tyche 450 K c) NMA Tyche 1750 K

## Glycine


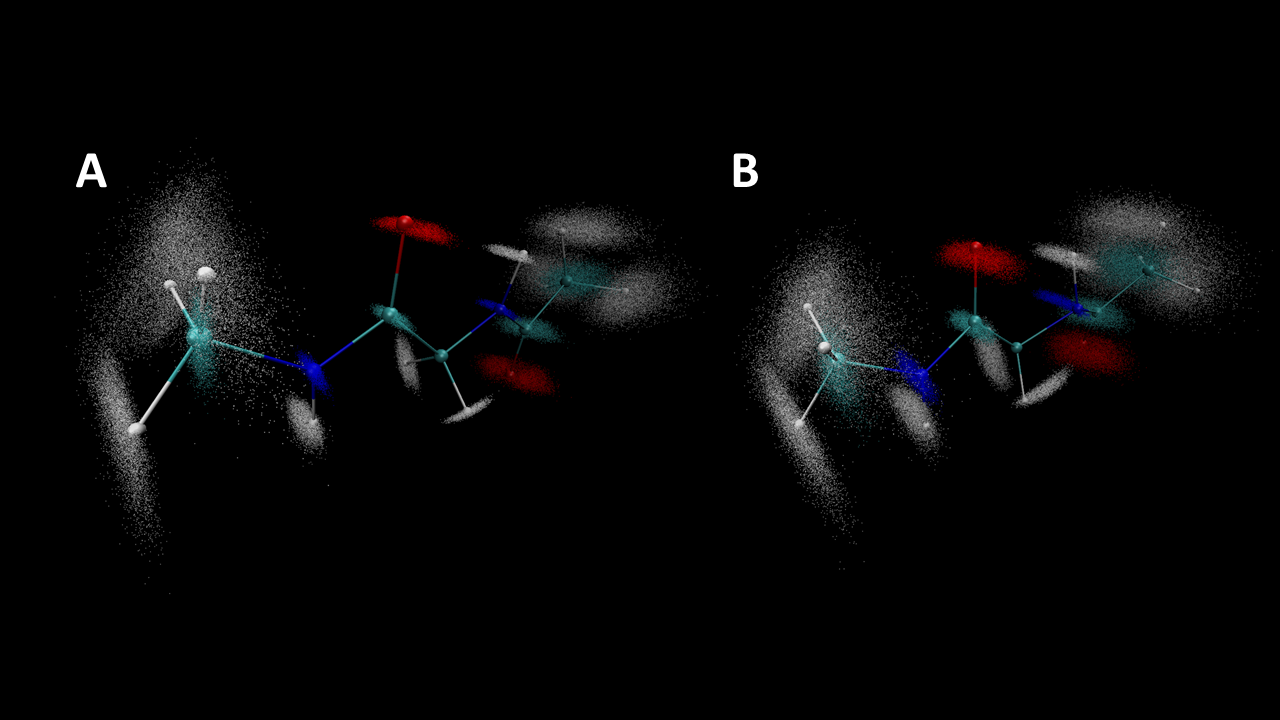


Figure S18. Mist plot for a) glycine Tyche 450 K b) glycine Tyche 1750 K

# S-Curves

## Water


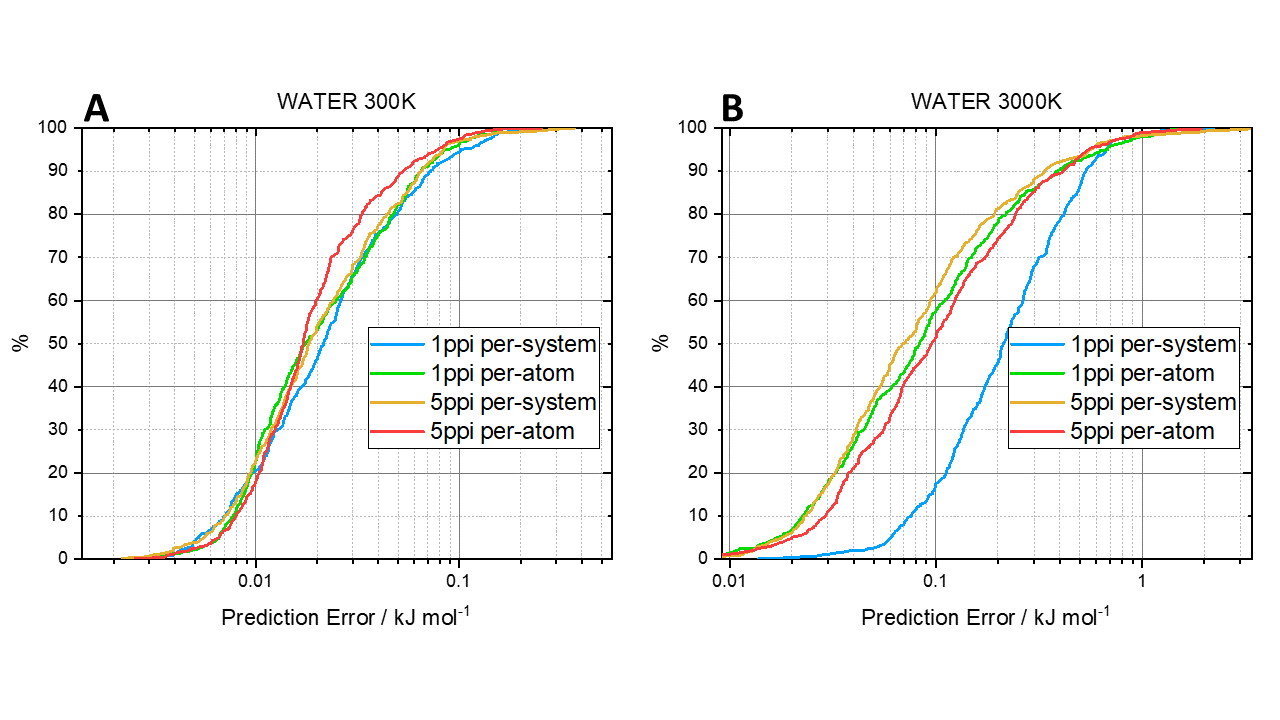


Figure S19. S-Curves for a) water 300 K b) water 3000 K

## Ammonia


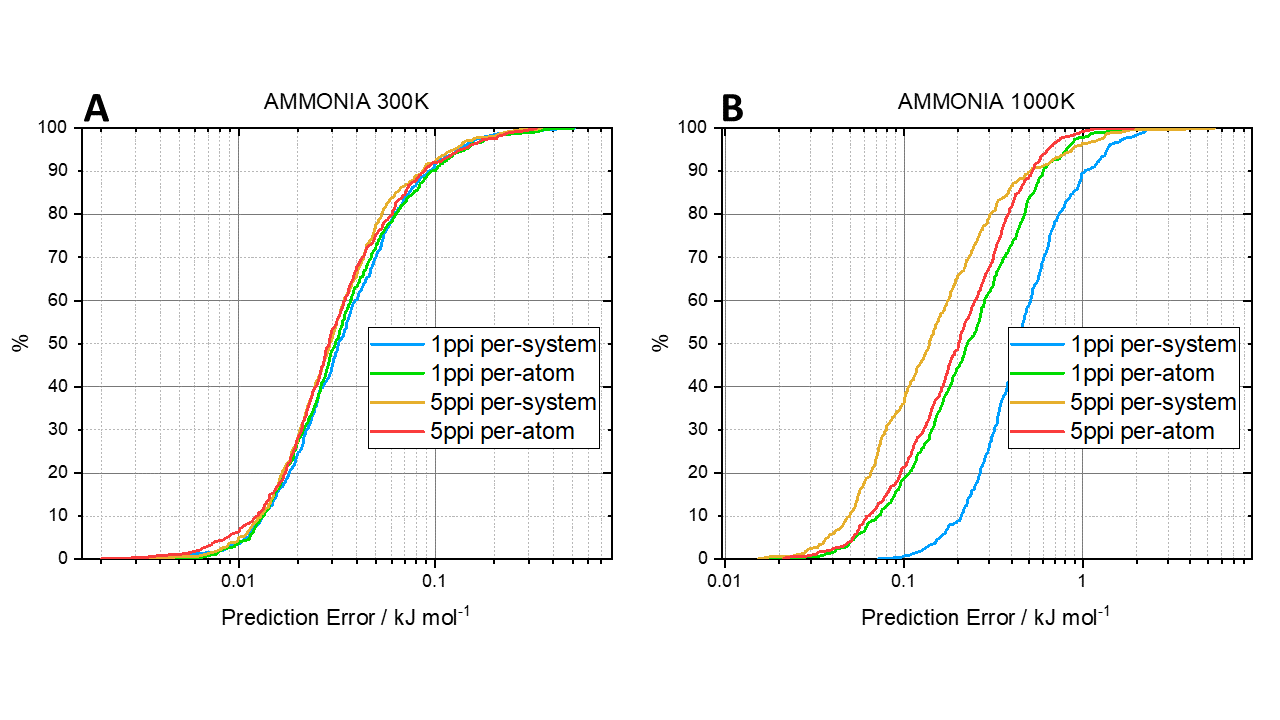


Figure S20. S-Curves for a) ammonia 300 K b) ammonia 1000 K

## Methanol


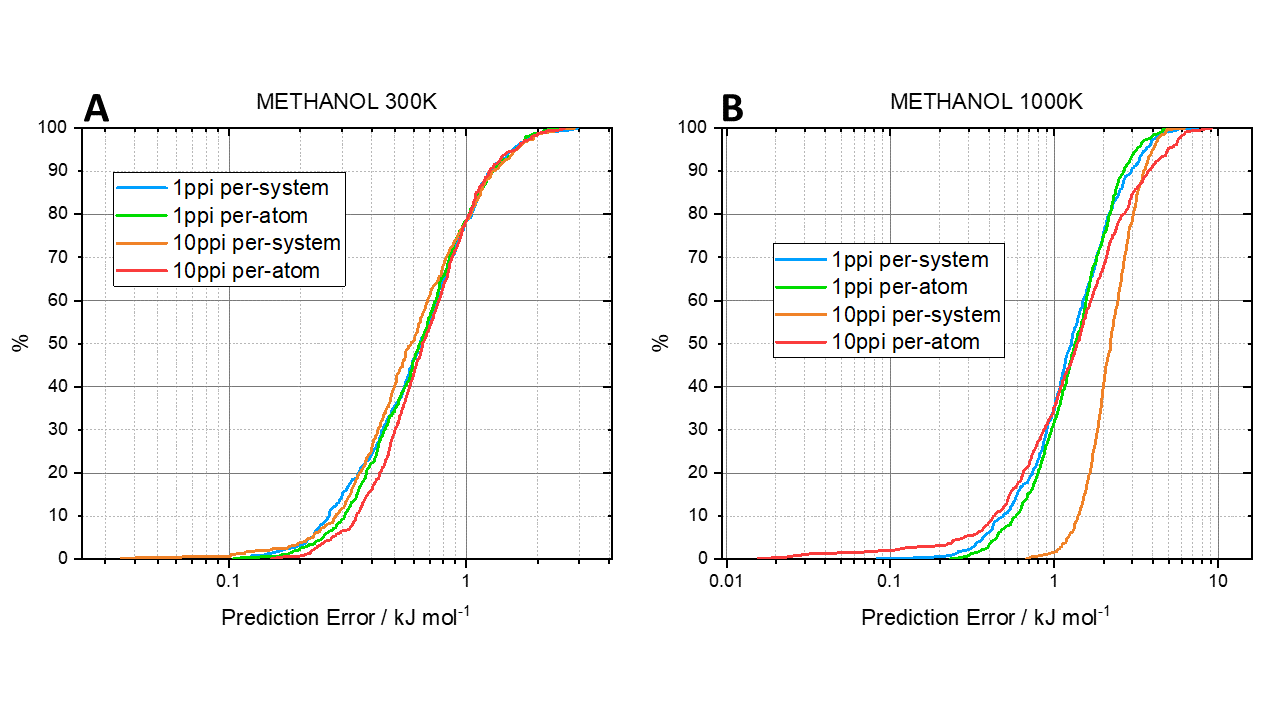


Figure S21. S-Curves for a) ammonia 300 K b) ammonia 1000 K

## Formamide


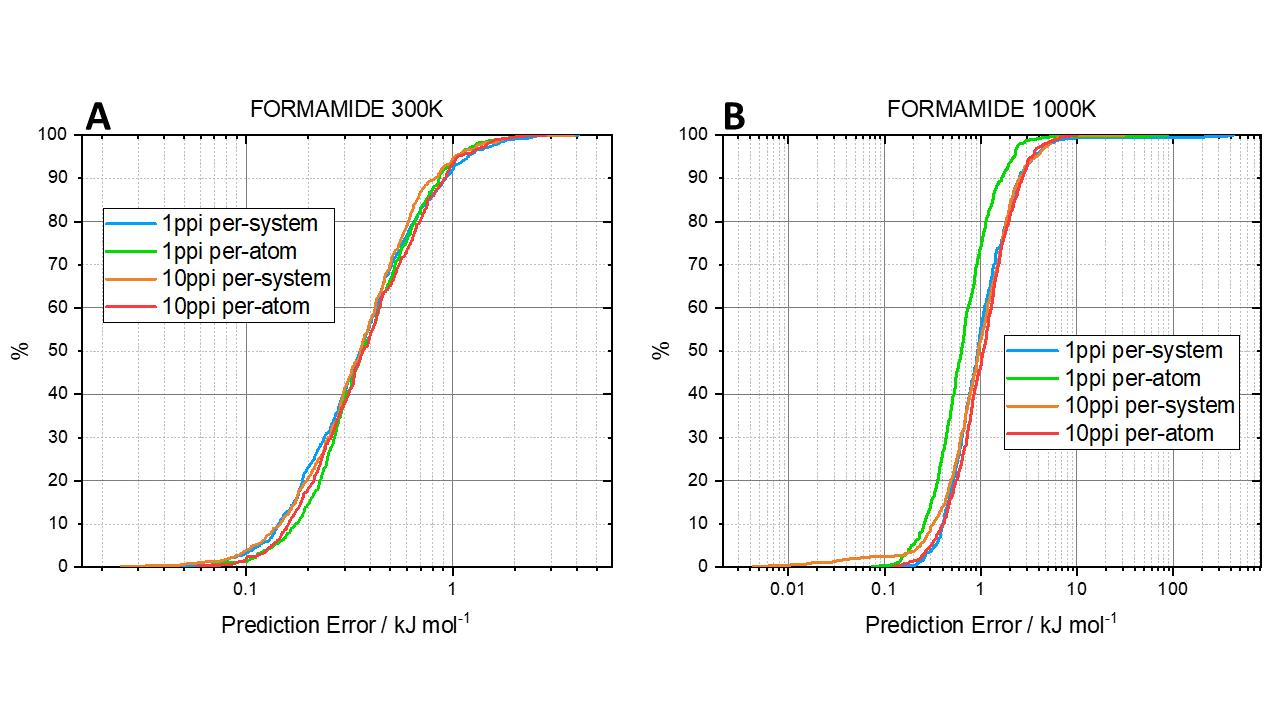


Figure S22. S-Curves for a) formamide 300 K b) formamide 1000 K

## Urea


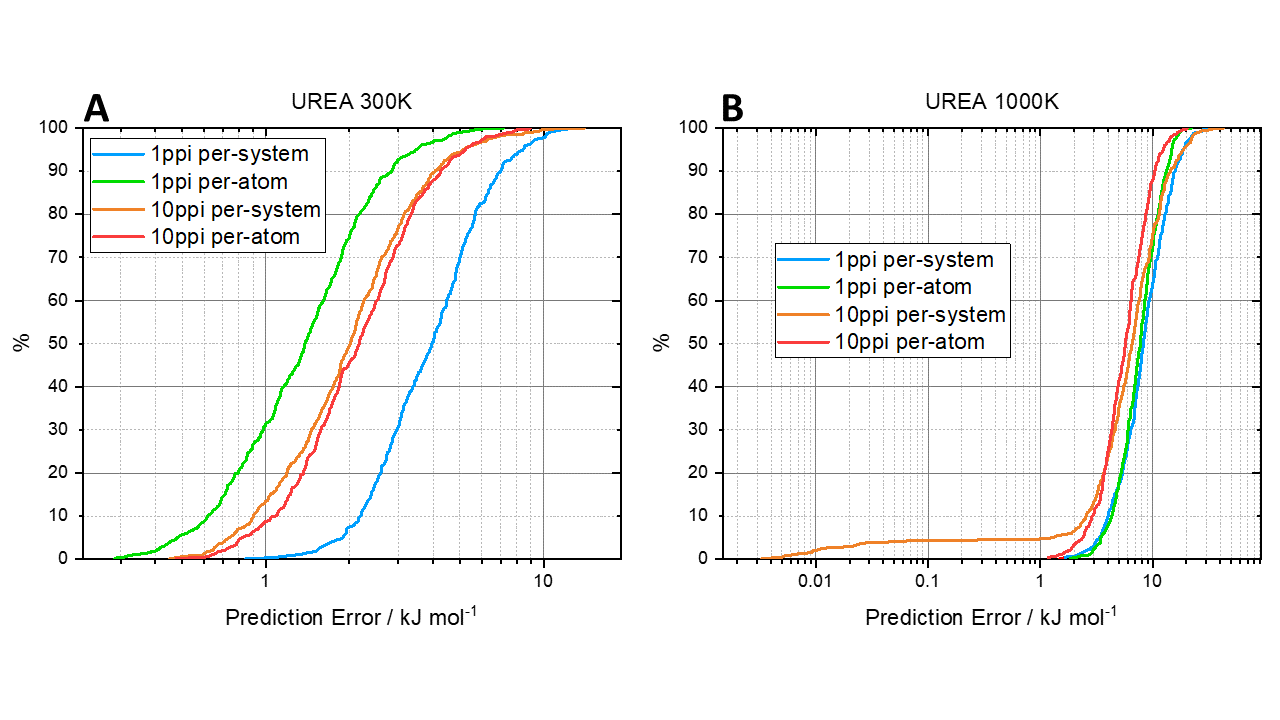


Figure S23. S-Curves for a) urea 300 K b) urea 1000 K

## Imidazole


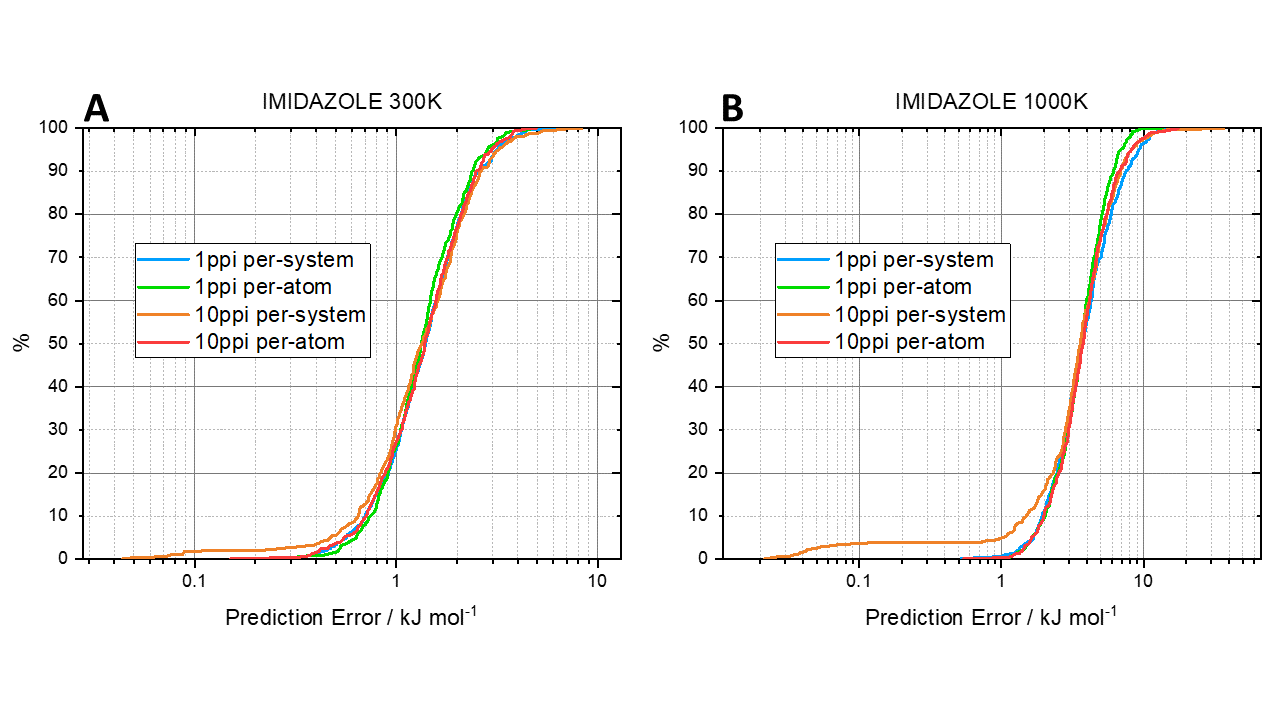


Figure S24. S-Curves for a) imidazole 300 K b) imidazole 1000 K

## NMA


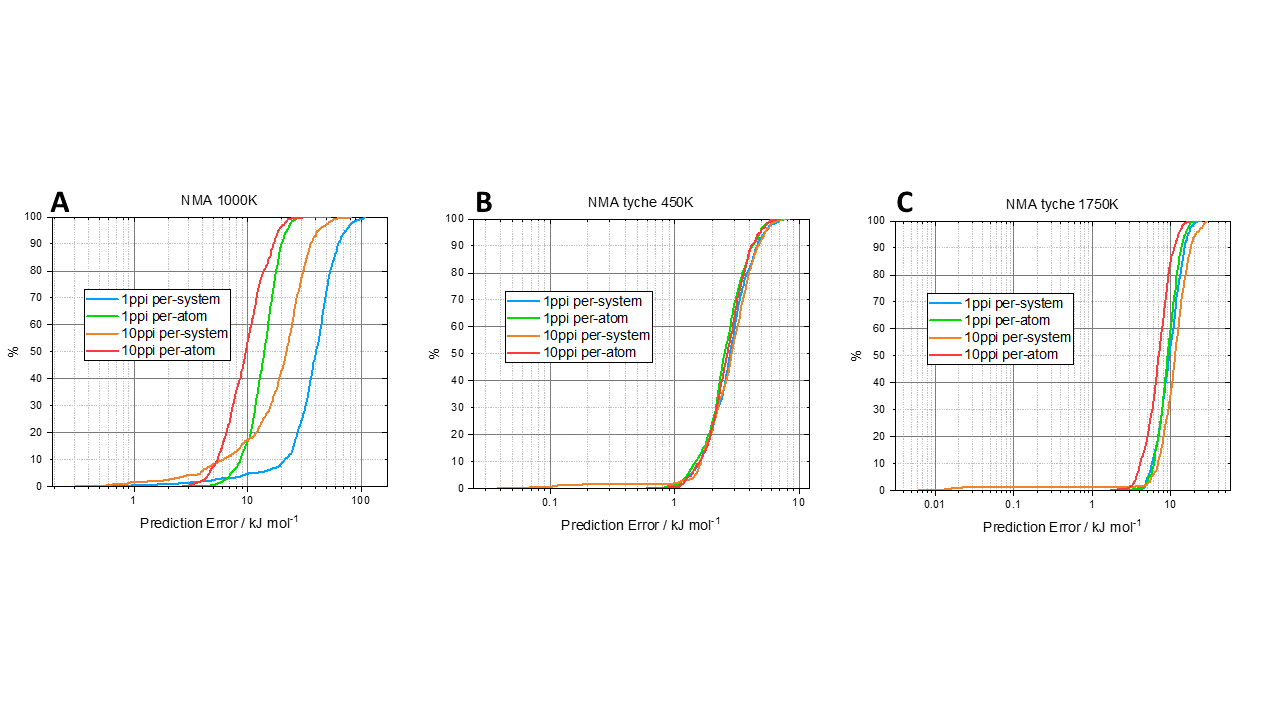


Figure S25. S-Curves for a) NMA 1000 K b) NMA Tyche 450 K c) NMA Tyche 1750 K

## Glycine


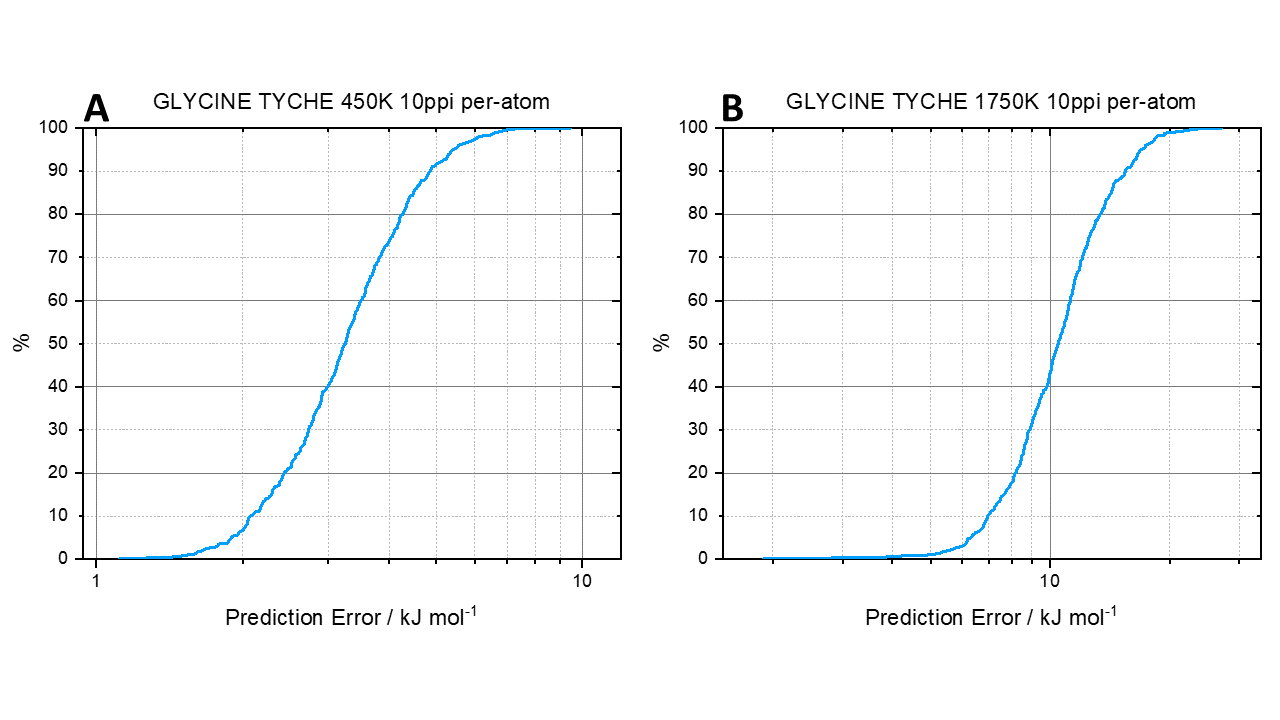


Figure S26. S-Curves for a) glycine Tyche 450 K b) glycine Tyche 1750 K

# Prediction Error Progression

## Water


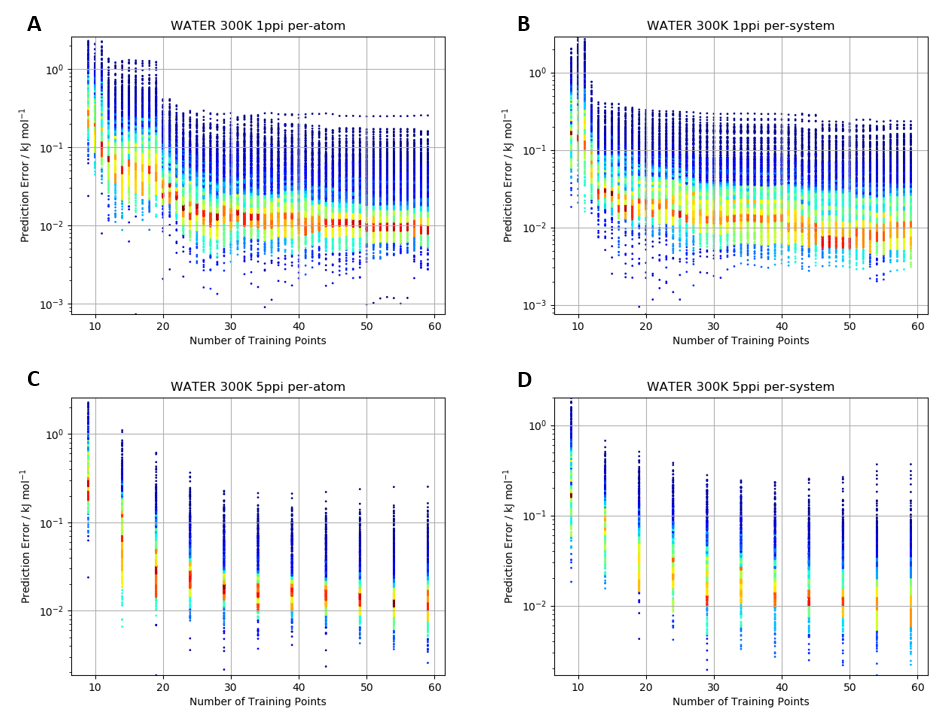


Figure S27. Prediction errors for the active learning run of water 300 K a) 1 point-per-iteration per-atom b) 1 point-per-iteration per-system c) 5 points-per-iteration per-atom d) 5 points-per-iteration per-system


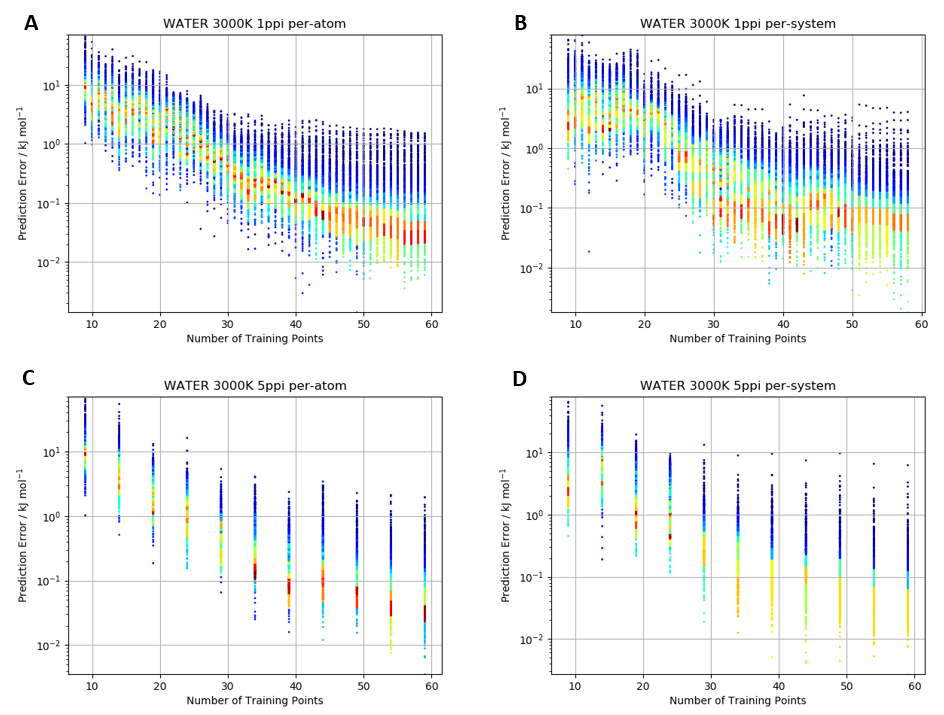


Figure S28. Prediction errors for the active learning run of water 3000 K a) 1 point-per-iteration per-atom b) 1 point-per-iteration per-system c) 5 points-per-iteration per-atom d) 5 points-per-iteration per-system

## Ammonia


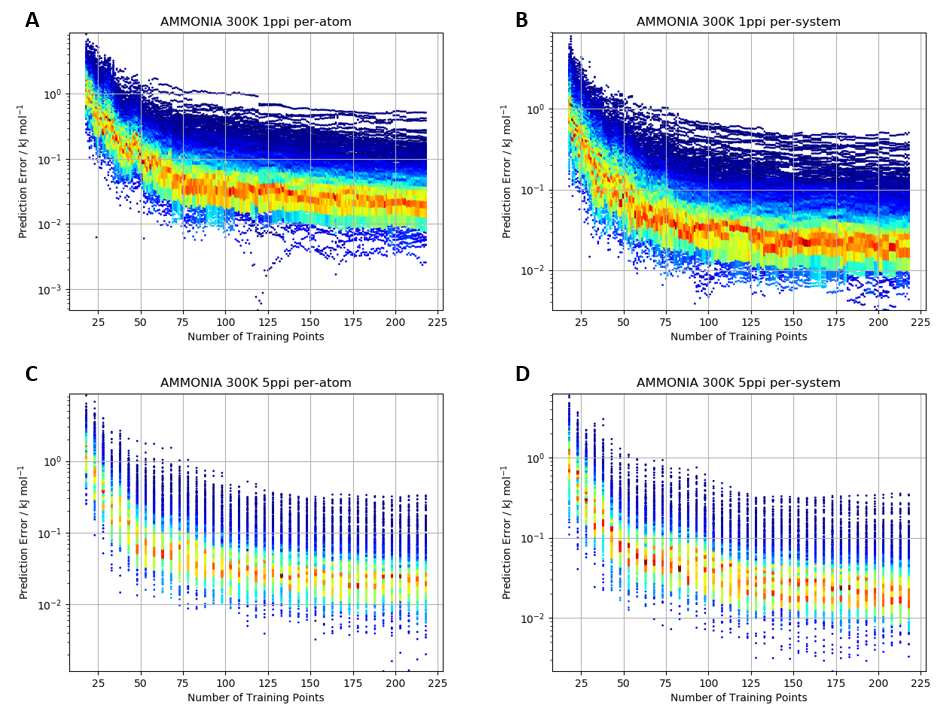


Figure S29. Prediction errors for the active learning run of ammonia 300 K a) 1 point-per-iteration per-atom b) 1 point-per-iteration per-system c) 5 points-per-iteration per-atom d) 5 points-per-iteration per-system


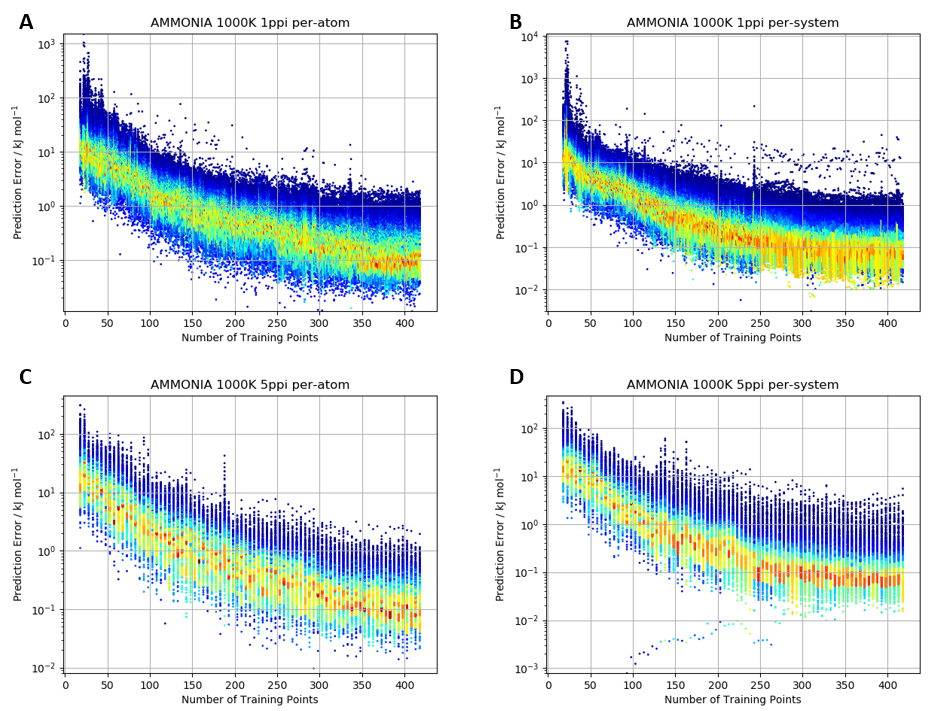


Figure S30. Prediction errors for the active learning run of ammonia 1000 K a) 1 point-per-iteration per-atom b) 1 point-per-iteration per-system c) 5 points-per-iteration per-atom d) 5 points-per-iteration per-system

## Methanol


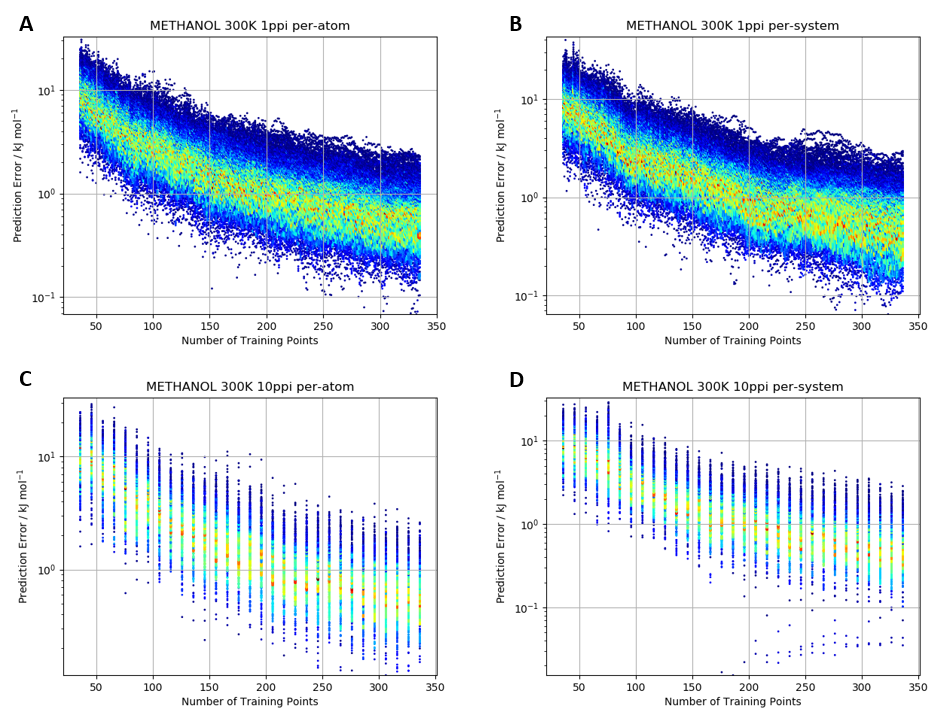


Figure S31. Prediction errors for the active learning run of methanol 300 K a) 1 point-per-iteration per-atom b) 1 point-per-iteration per-system c) 10 points-per-iteration per-atom d) 10 points-per-iteration per-system


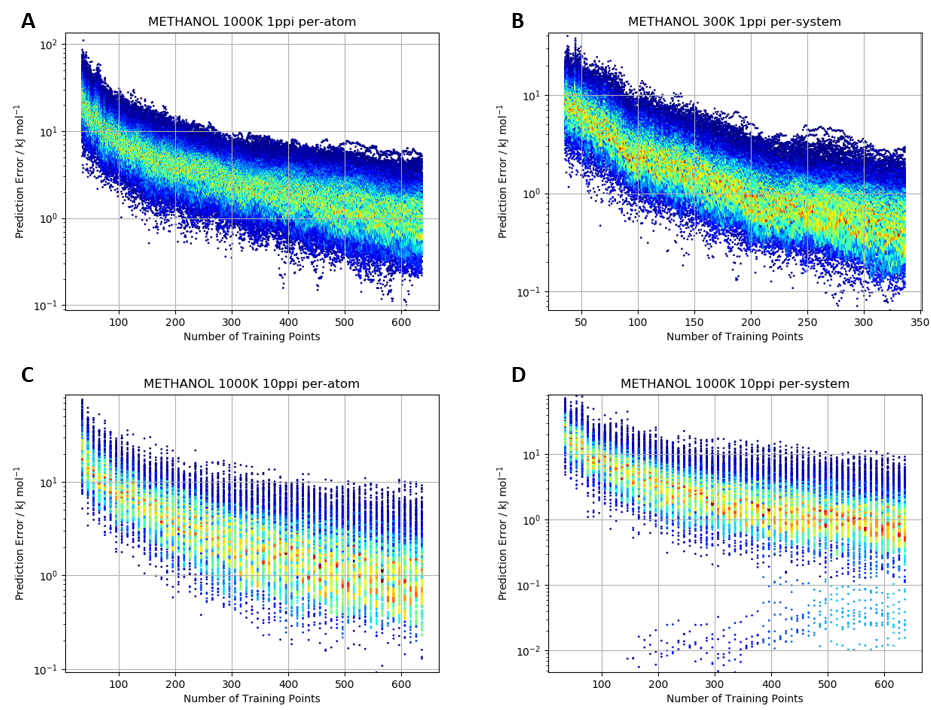


Figure S32. Prediction errors for the active learning run of methanol 1000 K a) 1 point-per-iteration per-atom b) 1 point-per-iteration per-system c) 10 points-per-iteration per-atom d) 10 points-per-iteration per-system

## Formamide


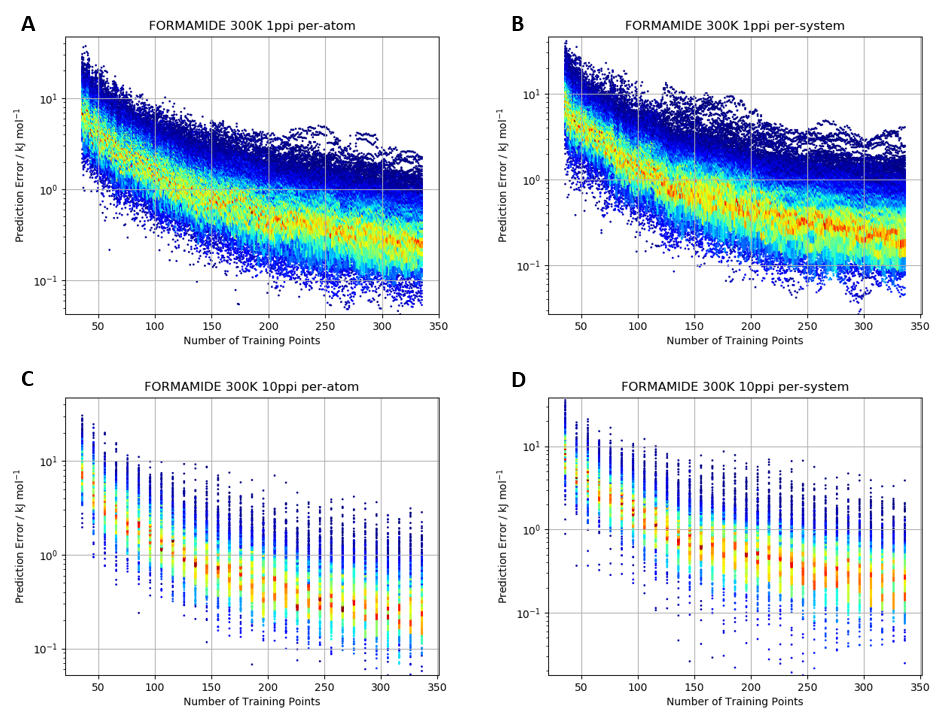


Figure S33. Prediction errors for the active learning run of formamide 300 K a) 1 point-per-iteration per-atom b) 1 point-per-iteration per-system c) 10 points-per-iteration per-atom d) 10 points-per-iteration per-system


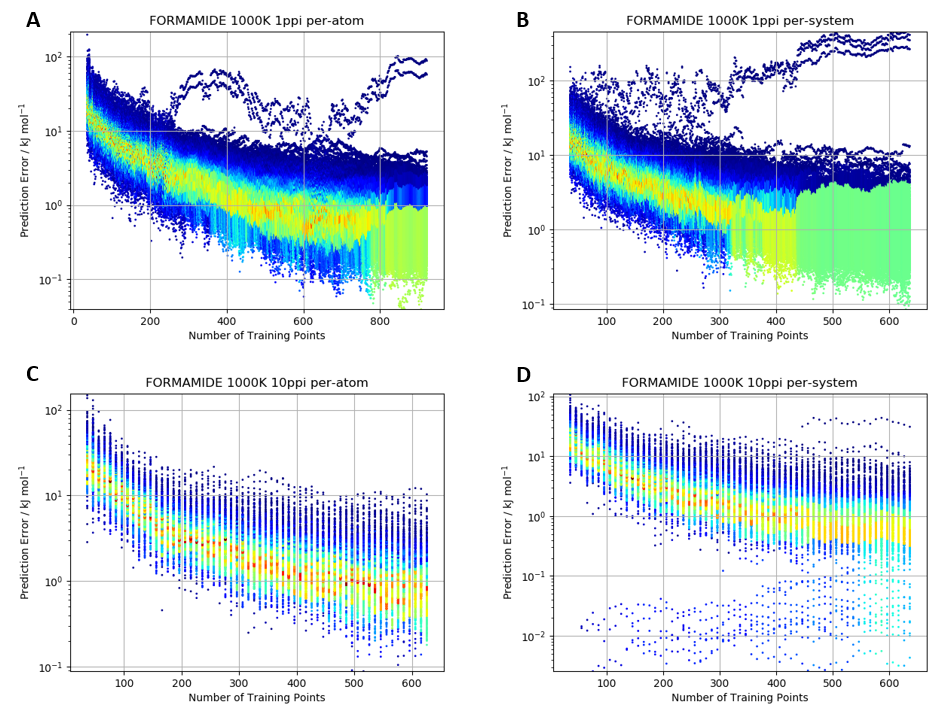


Figure S34. Prediction errors for the active learning run of formamide 1000 K a) 1 point-per-iteration per-atom b) 1 point-per-iteration per-system c) 10 points-per-iteration per-atom d) 10 points-per-iteration per-system

## Urea


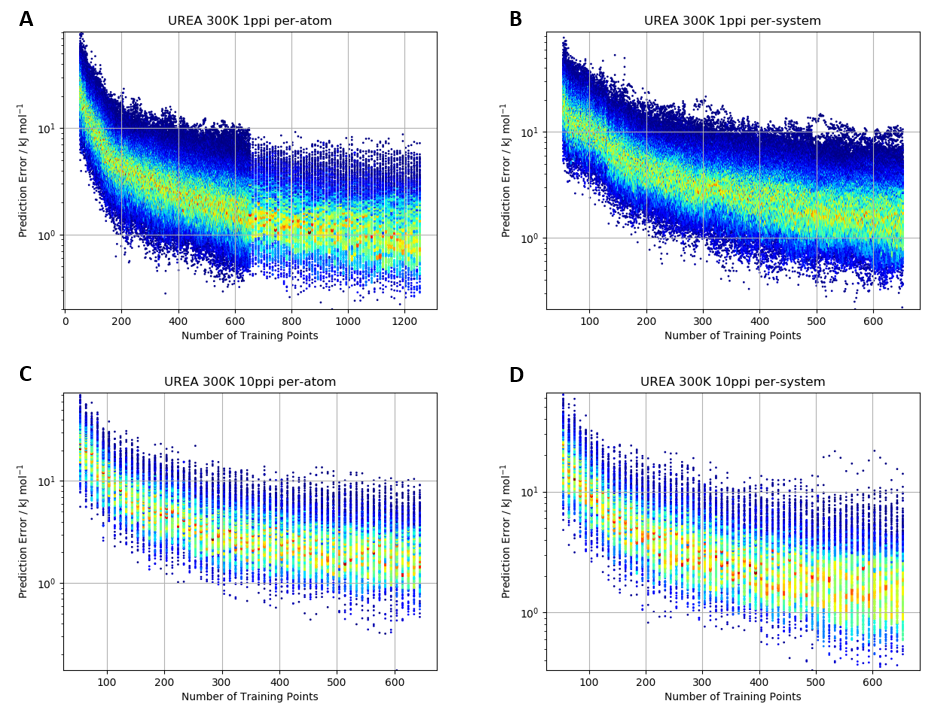


Figure S35. Prediction errors for the active learning run of urea 300 K a) 1 point-per-iteration per-atom b) 1 point-per-iteration per-system c) 10 points-per-iteration per-atom d) 10 points-per-iteration per-system


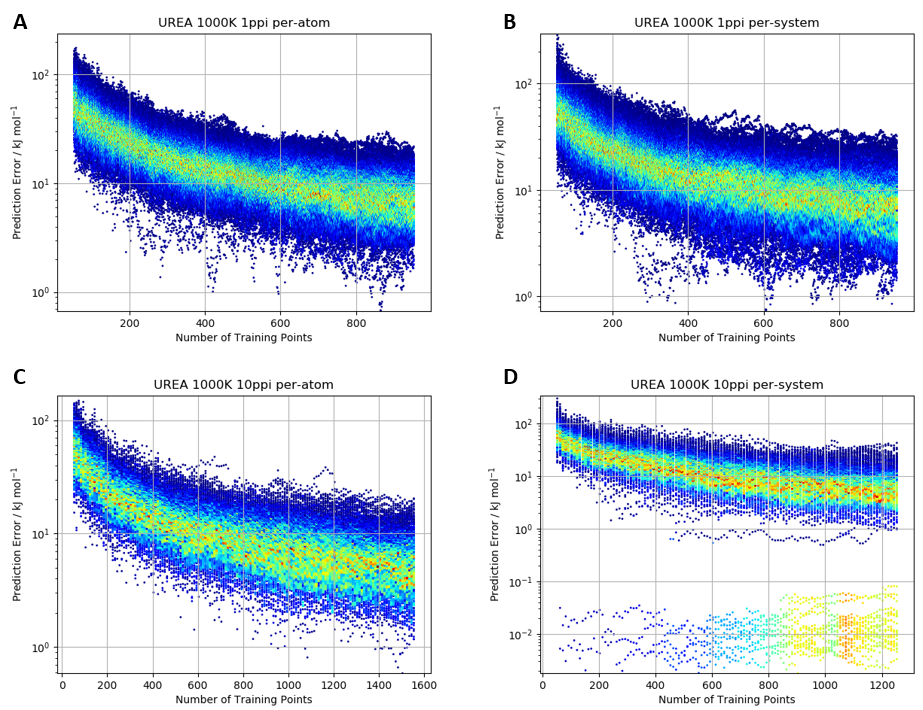


Figure S36. Prediction errors for the active learning run of urea 1000 K a) 1 point-per-iteration per-atom b) 1 point-per-iteration per-system c) 10 points-per-iteration per-atom d) 10 points-per-iteration per-system

## Imidazole


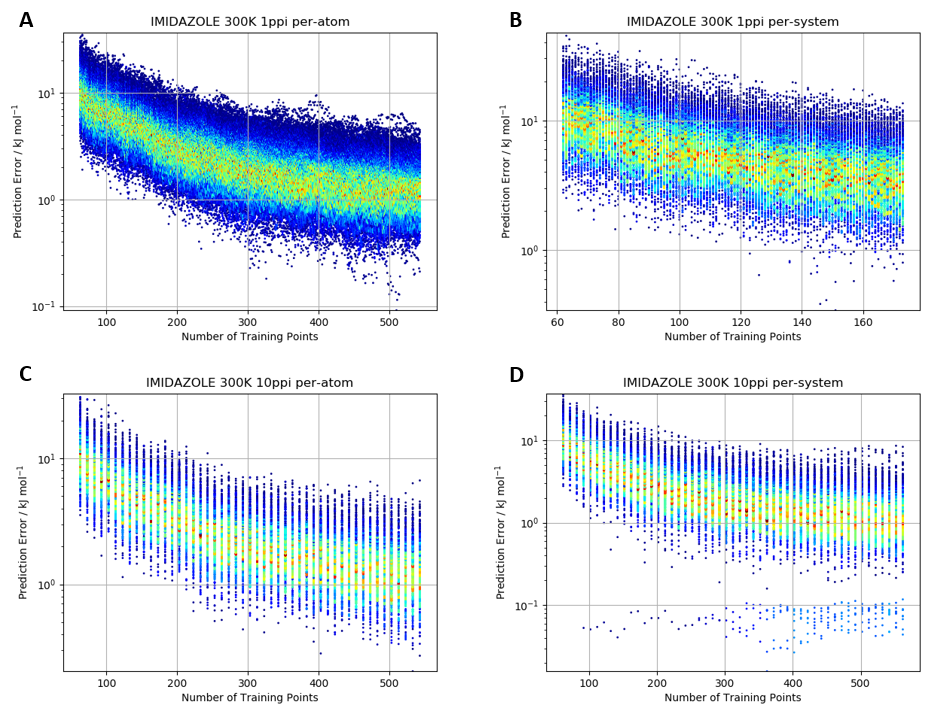


Figure S37. Prediction errors for the active learning run of imidazole 300 K a) 1 point-per-iteration per-atom b) 1 point-per-iteration per-system c) 10 points-per-iteration per-atom d) 10 points-per-iteration per-system


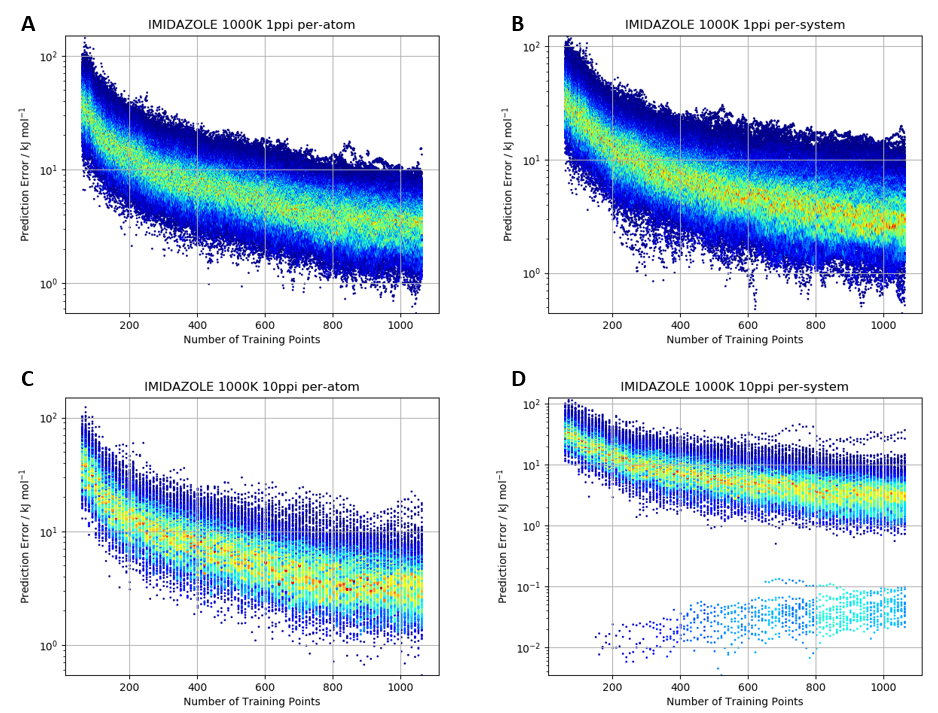


Figure S38. Prediction errors for the active learning run of imidazole 1000 K a) 1 point-per-iteration per-atom b) 1 point-per-iteration per-system c) 10 points-per-iteration per-atom d) 10 points-per-iteration per-system

## NMA


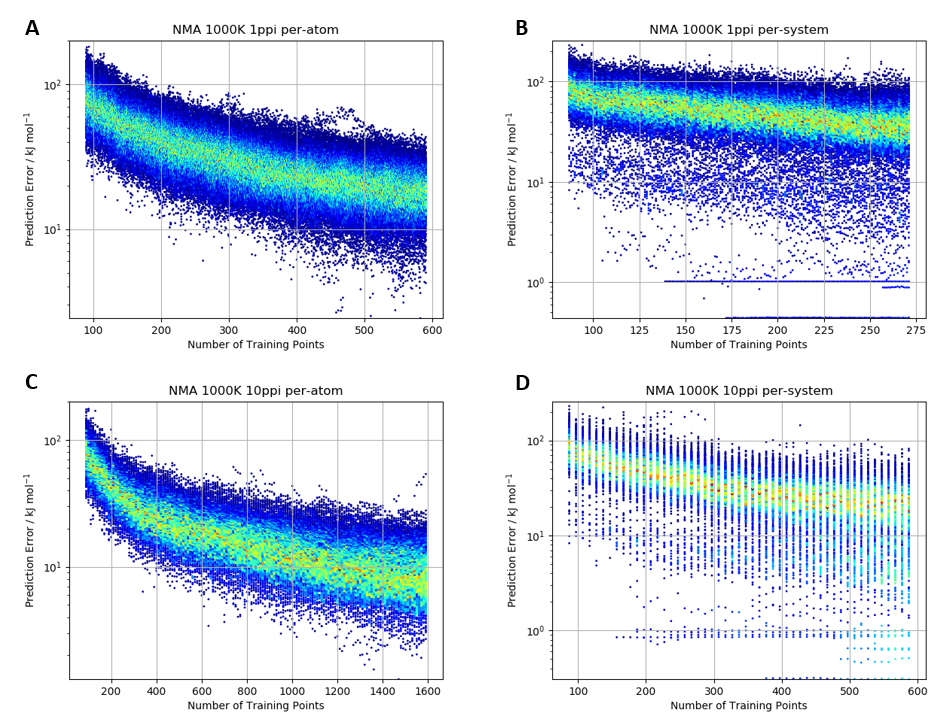


Figure S39. Prediction errors for the active learning run of NMA 1000 K a) 1 point-per-iteration per-atom b) 1 point-per-iteration per-system c) 10 points-per-iteration per-atom d) 10 points-per-iteration per-system


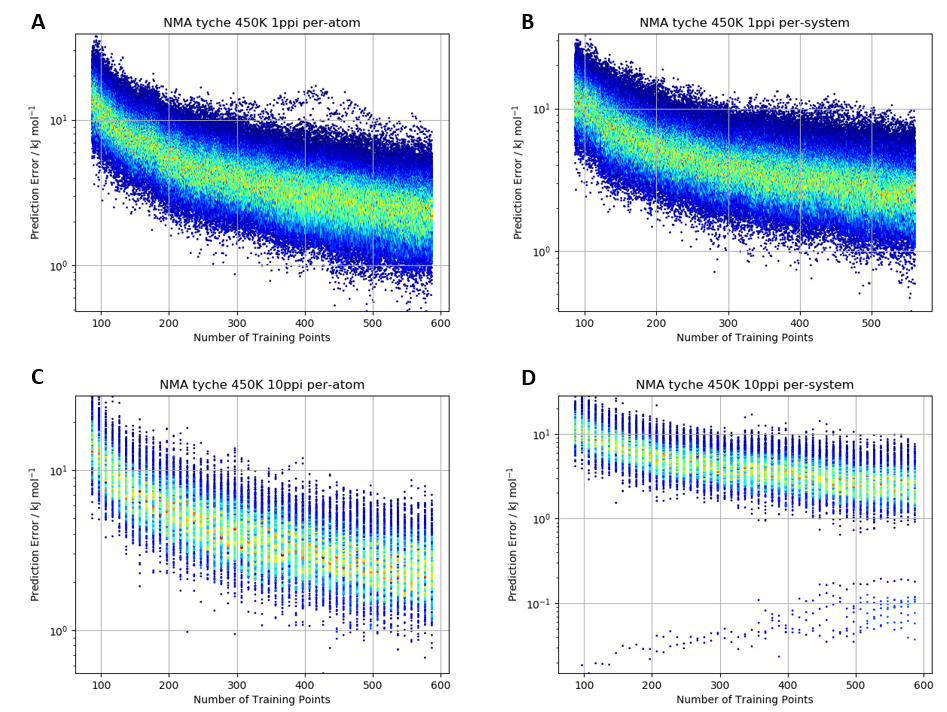


Figure S40. Prediction errors for the active learning run of NMA Tyche 450 K a) 1 point-per-iteration per-atom b) 1 point-per-iteration per-system c) 10 points-per-iteration per-atom d) 10 points-per-iteration per-system


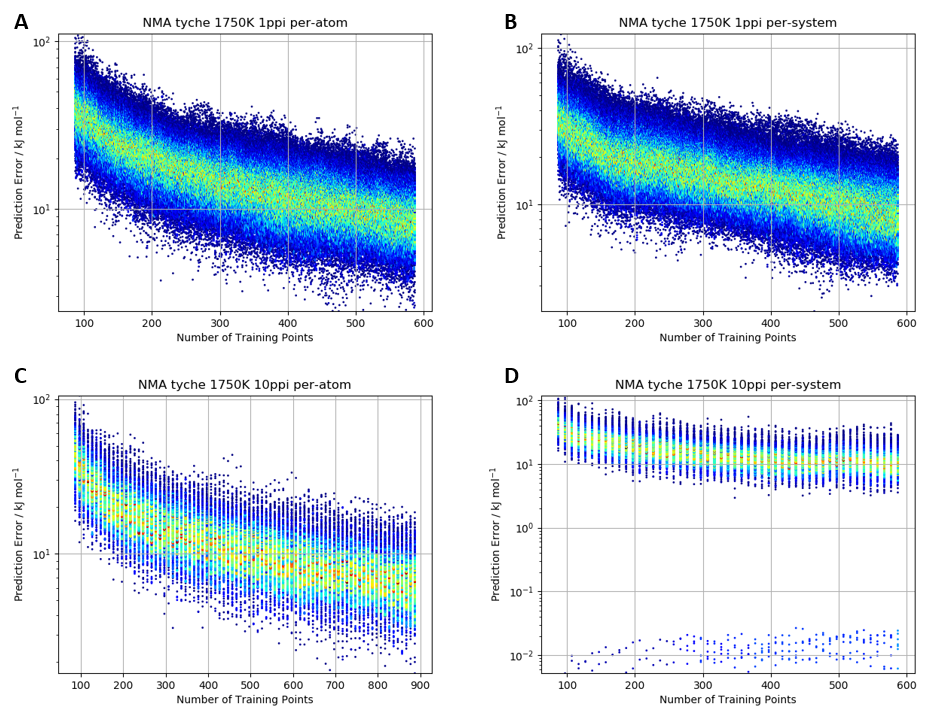


Figure S41. Prediction errors for the active learning run of NMA Tyche 1750 K a) 1 point-per-iteration per-atom b) 1 point-per-iteration per-system c) 10 points-per-iteration per-atom d) 10 points-per-iteration per-system

## Glycine


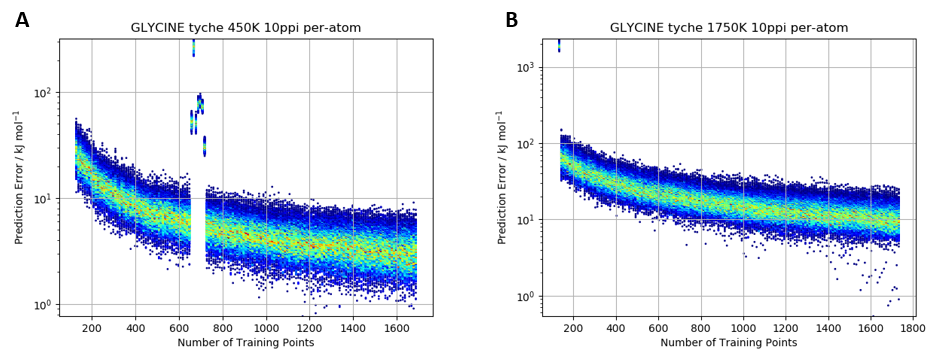


Figure S42. Prediction errors for the active learning run of glycine b) Tyche 450 K 10 points-per-iteration per-atom a) Tyche 1750 K 10 points-per-iteration per-atom

# True vs Predicted

## Water


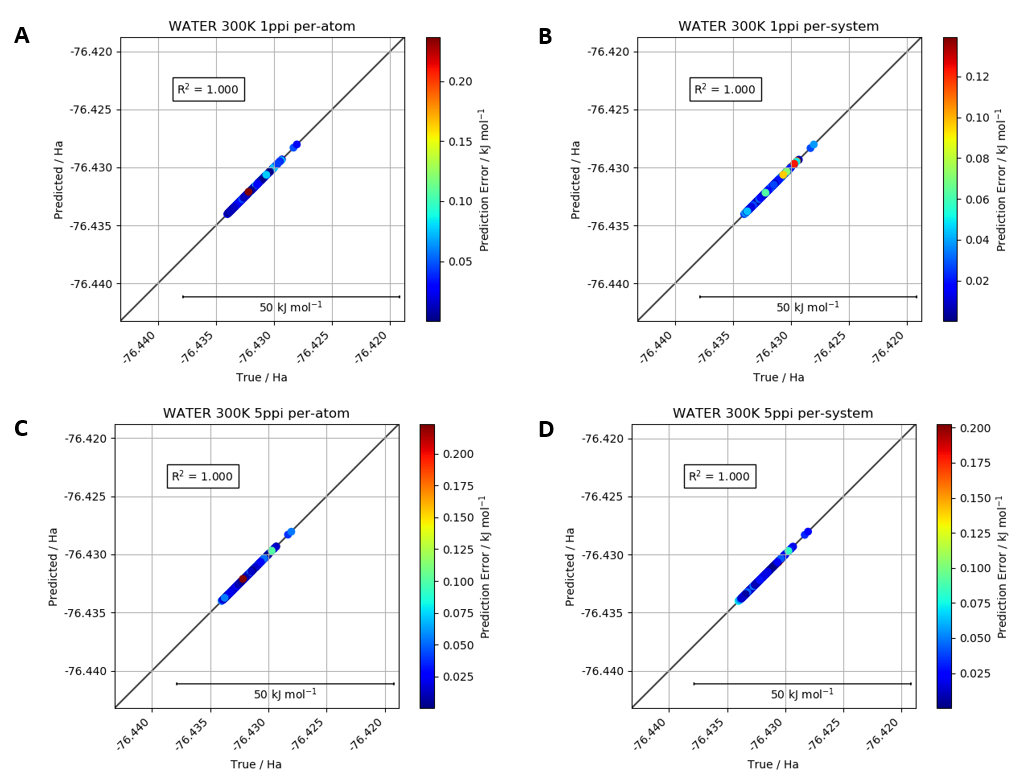


Figure S43. Prediction errors for the active learning run of water 300 K a) 1 point-per-iteration per-atom b) 1 point-per-iteration per-system c) 5 points-per-iteration per-atom d) 5 points-per-iteration per-system


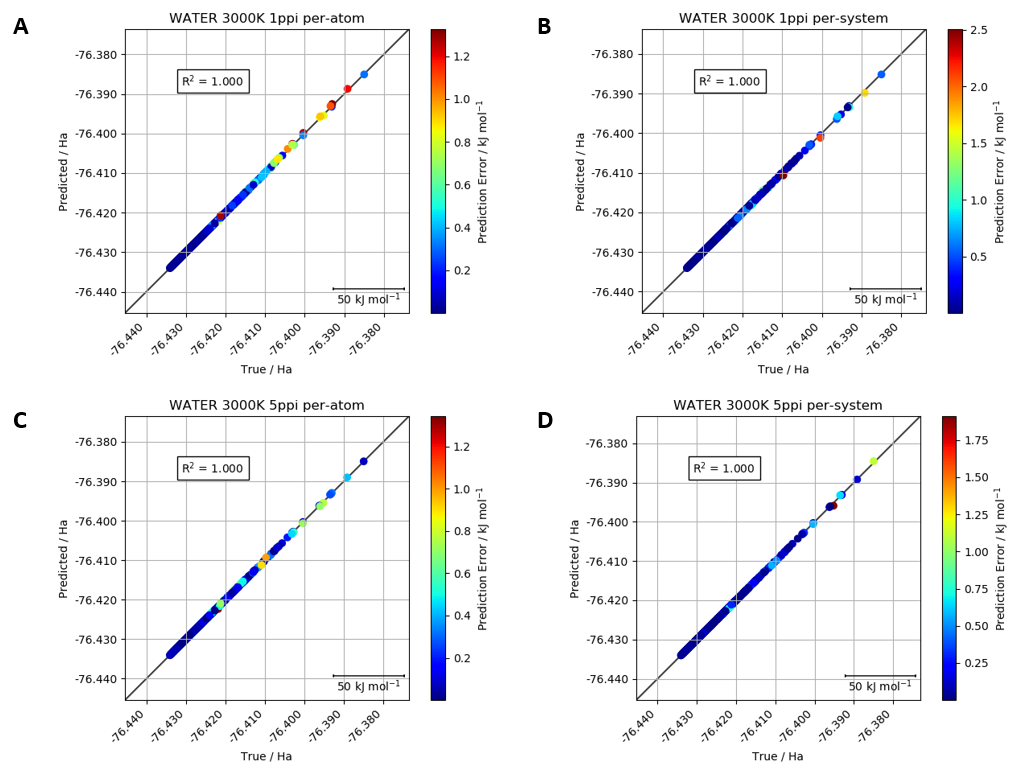


Figure S44. Prediction errors for the active learning run of water 3000 K a) 1 point-per-iteration per-atom b) 1 point-per-iteration per-system c) 5 points-per-iteration per-atom d) 5 points-per-iteration per-system

## Ammonia


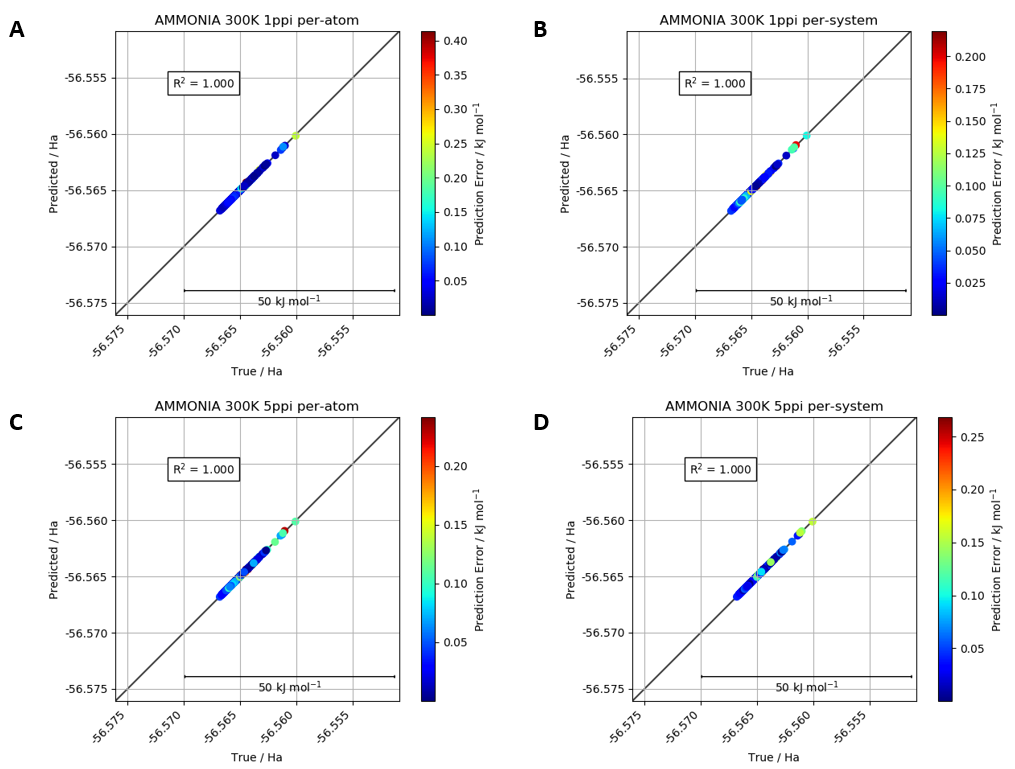


Figure S45. Prediction errors for the active learning run of ammonia 300 K a) 1 point-per-iteration per-atom b) 1 point-per-iteration per-system c) 5 points-per-iteration per-atom d) 5 points-per-iteration per-system


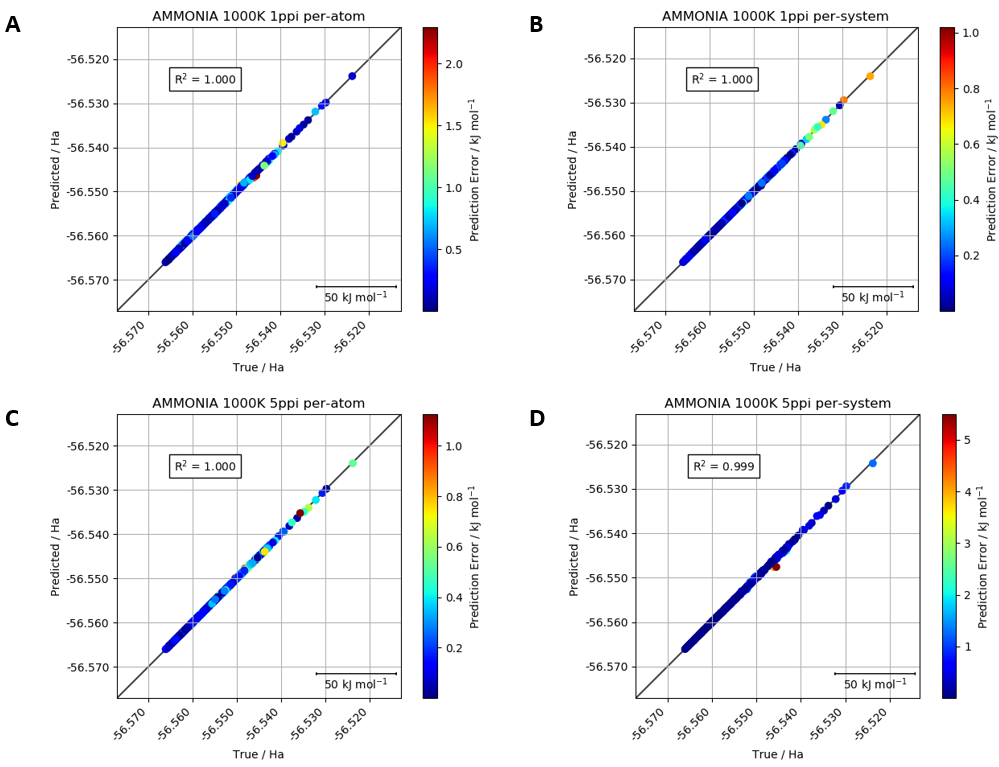


Figure S46. Prediction errors for the active learning run of ammonia 1000 K a) 1 point-per-iteration per-atom b) 1 point-per-iteration per-system c) 5 points-per-iteration per-atom d) 5 points-per-iteration per-system

## Methanol


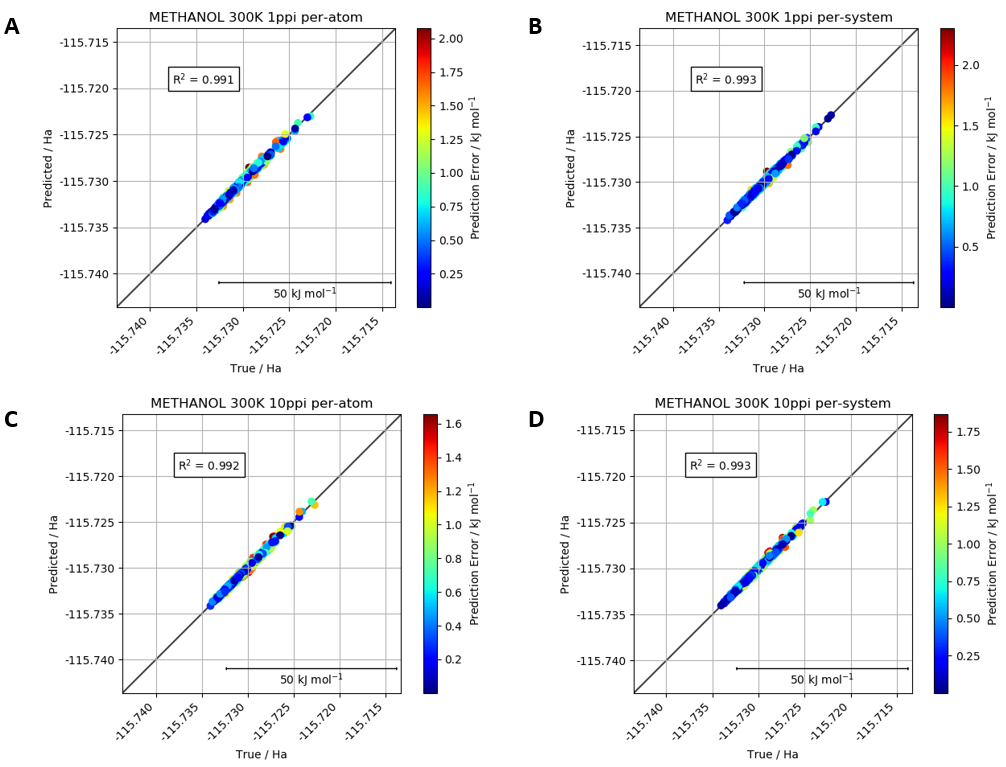


Figure S47. Prediction errors for the active learning run of methanol 300 K a) 1 point-per-iteration per-atom b) 1 point-per-iteration per-system c) 10 points-per-iteration per-atom d) 10 points-per-iteration per-system


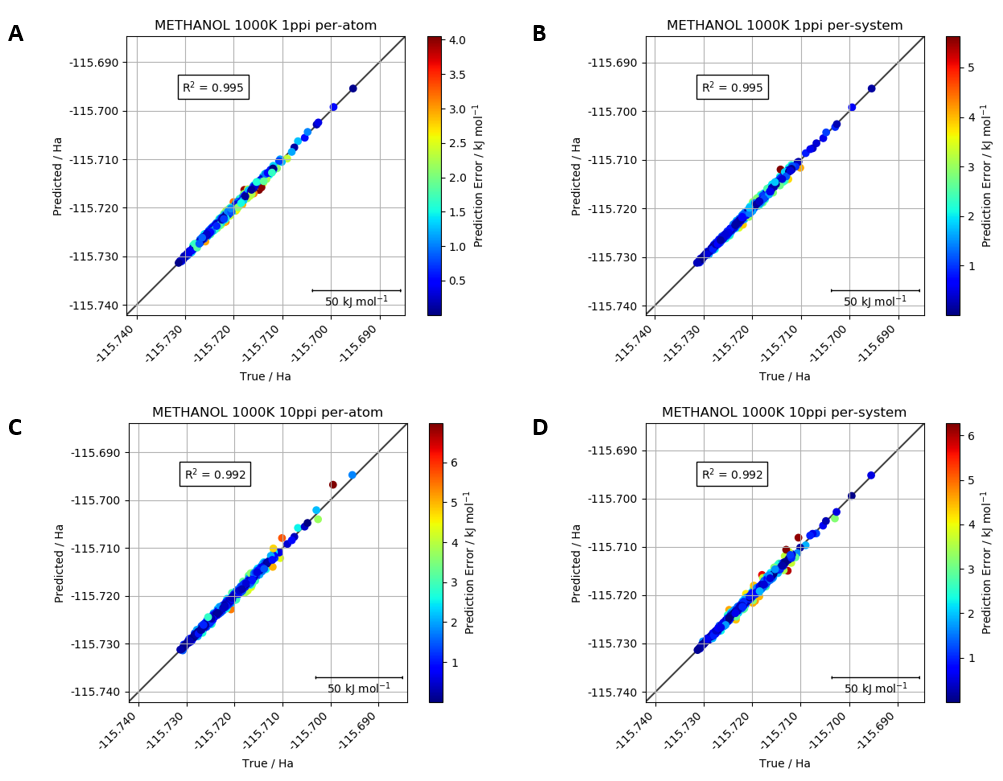


Figure S48. Prediction errors for the active learning run of methanol 1000 K a) 1 point-per-iteration per-atom b) 1 point-per-iteration per-system c) 10 points-per-iteration per-atom d) 10 points-per-iteration per-system

## Formamide


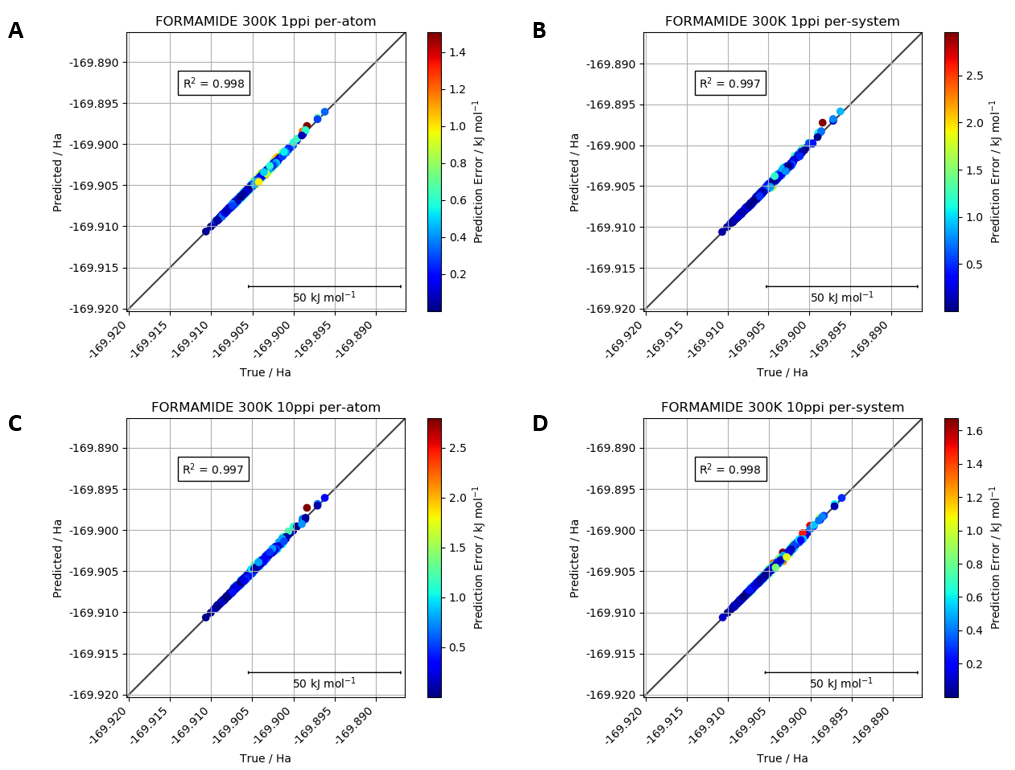


Figure S49. Prediction errors for the active learning run of formamide 300 K a) 1 point-per-iteration per-atom b) 1 point-per-iteration per-system c) 10 points-per-iteration per-atom d) 10 points-per-iteration per-system


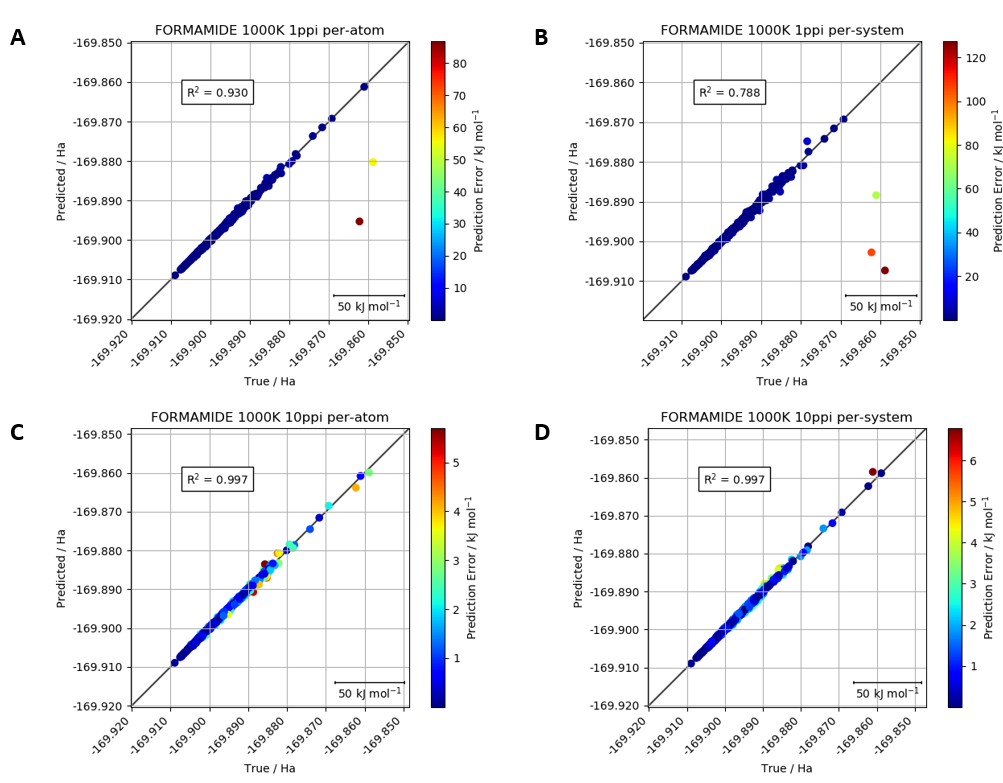


Figure S50. Prediction errors for the active learning run of formamide 1000 K a) 1 point-per-iteration per-atom b) 1 point-per-iteration per-system c) 10 points-per-iteration per-atom d) 10 points-per-iteration per-system

## Urea


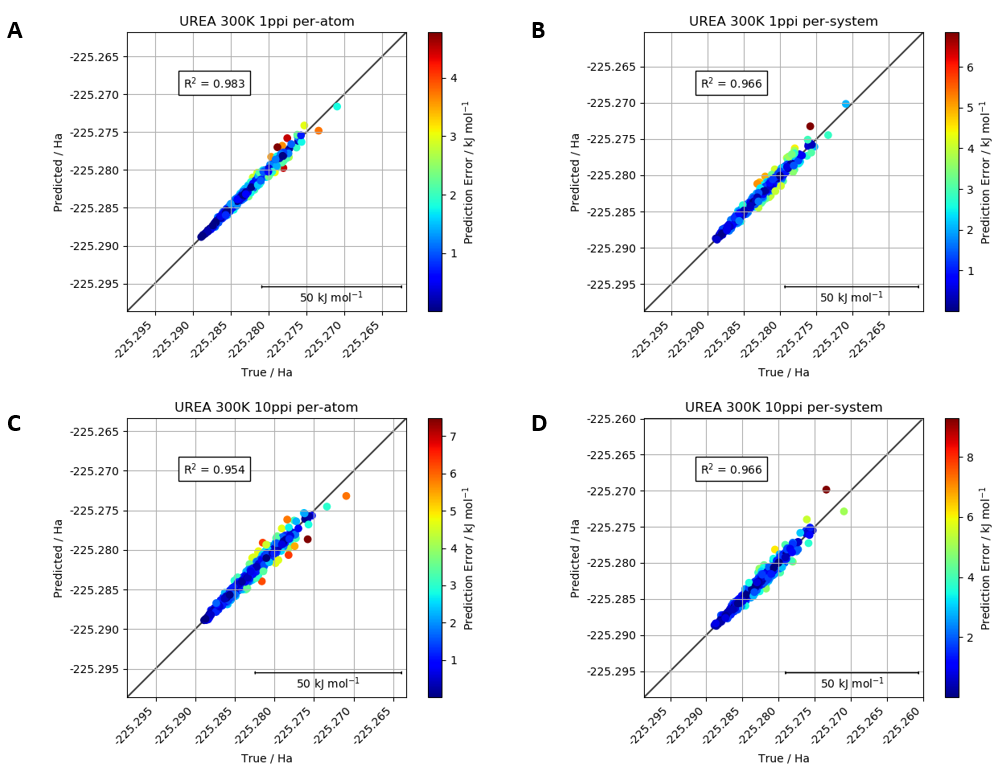


Figure S51. Prediction errors for the active learning run of urea 300 K a) 1 point-per-iteration per-atom b) 1 point-per-iteration per-system c) 10 points-per-iteration per-atom d) 10 points-per-iteration per-system


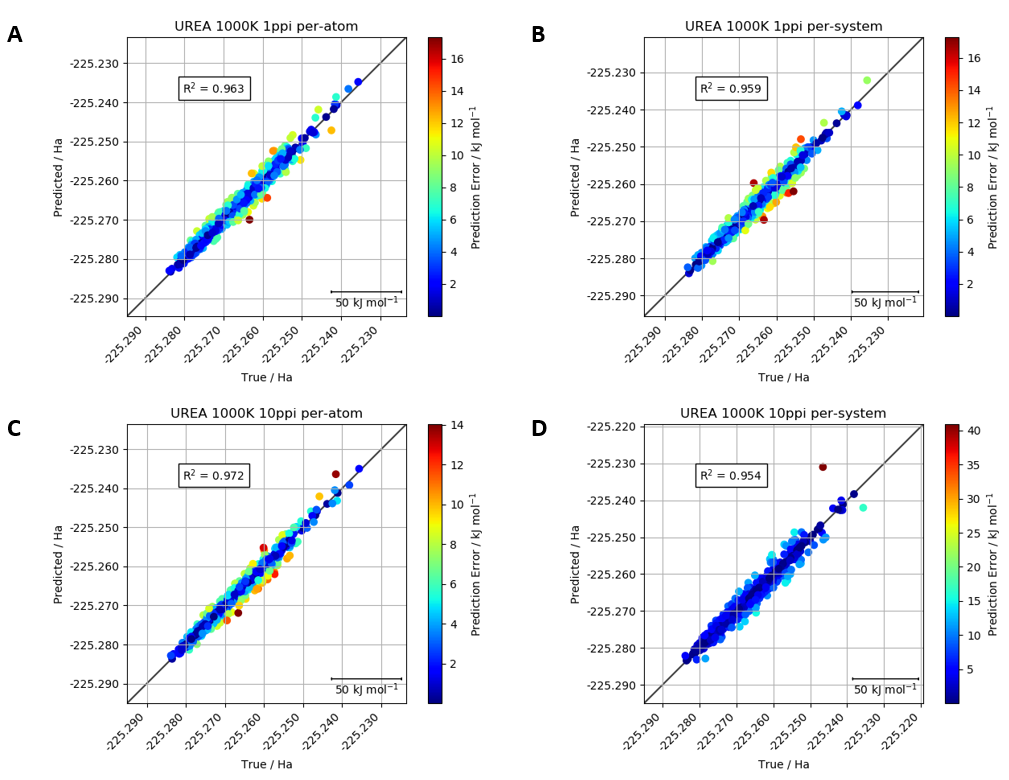


Figure S52. Prediction errors for the active learning run of urea 1000 K a) 1 point-per-iteration per-atom b) 1 point-per-iteration per-system c) 10 points-per-iteration per-atom d) 10 points-per-iteration per-system

## Imidazole


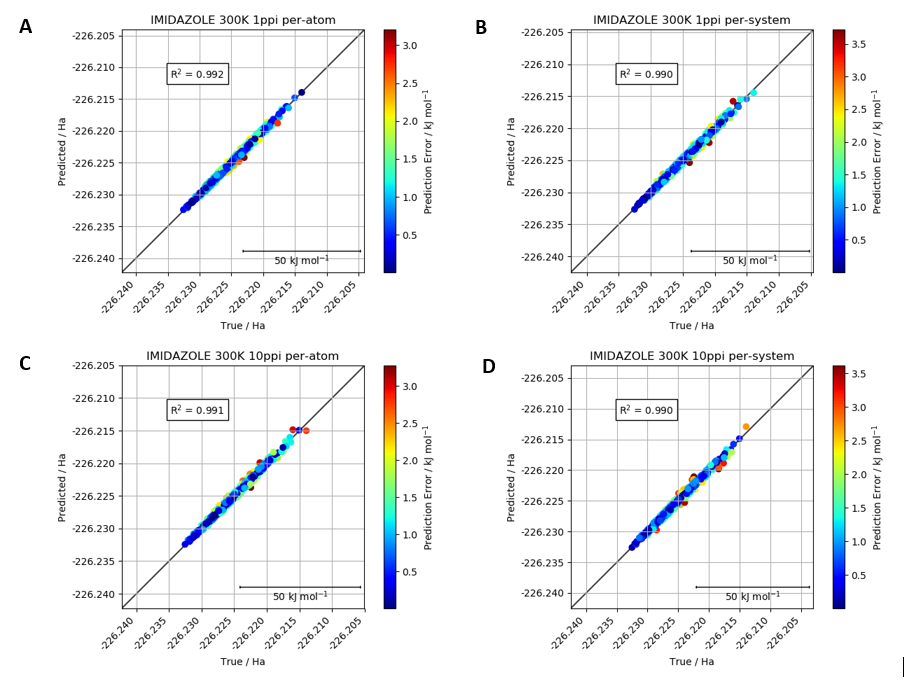


Figure S53. Prediction errors for the active learning run of imidazole 300 K a) 1 point-per-iteration per-atom b) 1 point-per-iteration per-system c) 10 points-per-iteration per-atom d) 10 points-per-iteration per-system


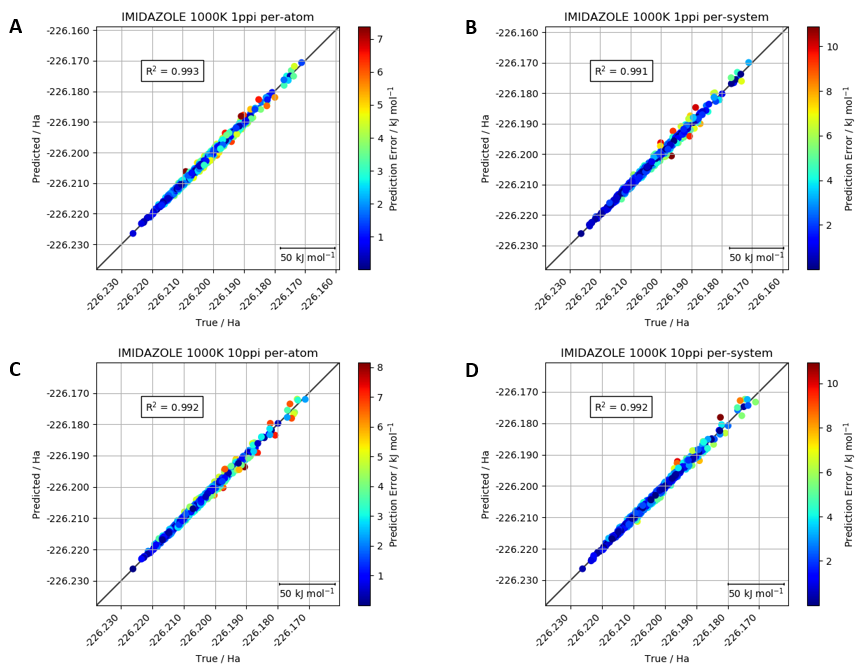


Figure S54. Prediction errors for the active learning run of imidazole 1000 K a) 1 point-per-iteration per-atom b) 1 point-per-iteration per-system c) 10 points-per-iteration per-atom d) 10 points-per-iteration per-system

## NMA


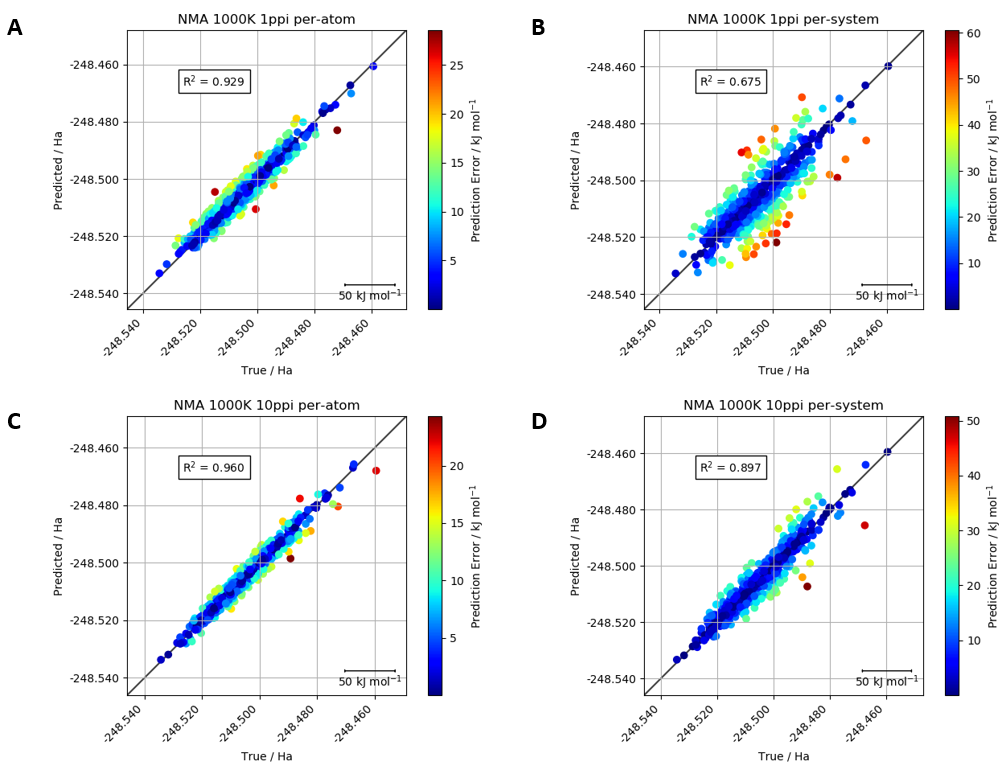


Figure S55. Prediction errors for the active learning run of NMA 1000 K a) 1 point-per-iteration per-atom b) 1 point-per-iteration per-system c) 10 points-per-iteration per-atom d) 10 points-per-iteration per-system


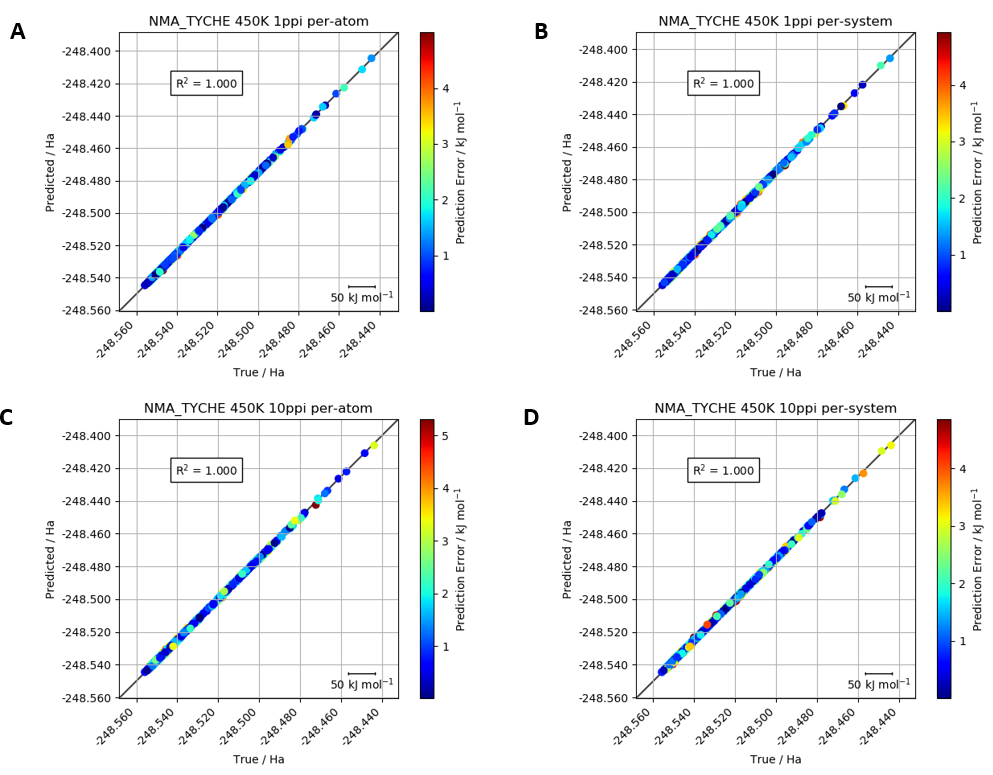


Figure S56. Prediction errors for the active learning run of NMA Tyche 450 K a) 1 point-per-iteration per-atom b) 1 point-per-iteration per-system c) 10 points-per-iteration per-atom d) 10 points-per-iteration per-system


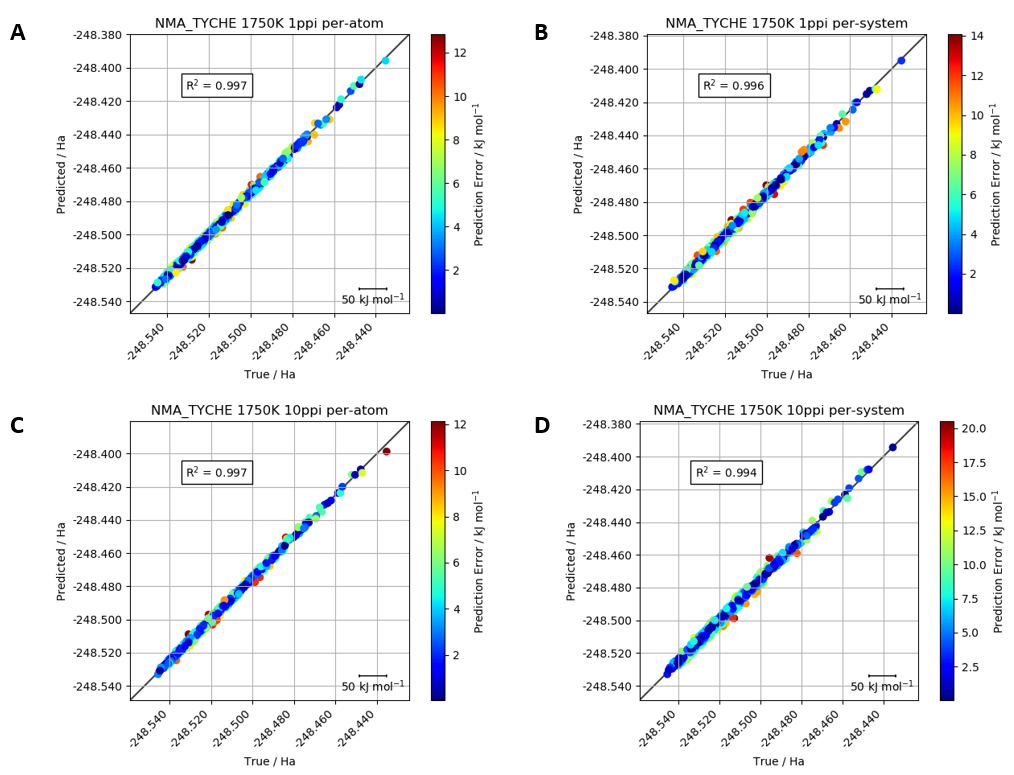


Figure S57. Prediction errors for the active learning run of NMA Tyche 1750 K a) 1 point-per-iteration per-atom b) 1 point-per-iteration per-system c) 10 points-per-iteration per-atom d) 10 points-per-iteration per-system

## Glycine


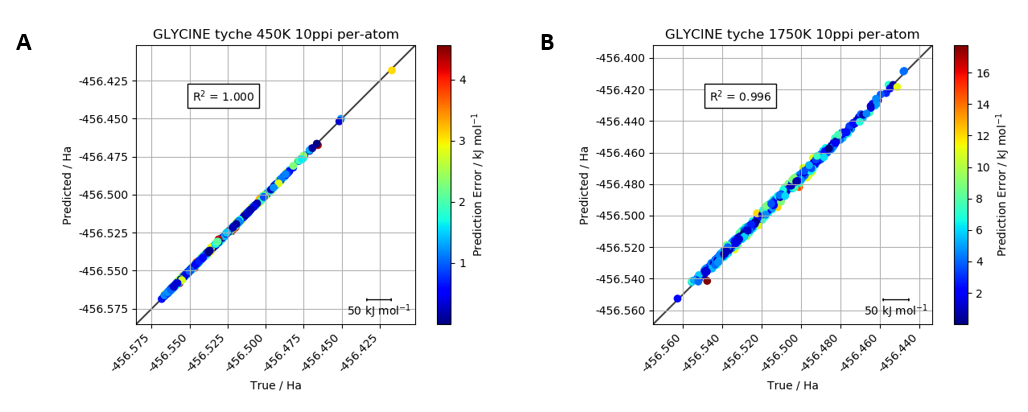


Figure S58. Prediction errors for the active learning run of glycine a) Tyche 450 K 10 points-per-iteration per-atom b) Tyche 1750 K 10 points-per-iteration per-atom

1. Mills, M. J. L.; Popelier, P. L. A., Electrostatic Forces: formulae for the first derivatives of a polarisable, anisotropic electrostatic potential energy function based on machine learning. *J.Chem.Theory Comput.* **2014,** *10*, 3840−3856.
